# The Skin Microbiome in Healthy and Allergic Dogs

**DOI:** 10.1371/journal.pone.0083197

**Published:** 2014-01-08

**Authors:** Aline Rodrigues Hoffmann, Adam P. Patterson, Alison Diesel, Sara D. Lawhon, Hoai Jaclyn Ly, Christine Elkins Stephenson, Joanne Mansell, Jörg M. Steiner, Scot E. Dowd, Thierry Olivry, Jan S. Suchodolski

**Affiliations:** 1 Dermatopathology Specialty Service, Department of Veterinary Pathobiology, College of Veterinary Medicine & Biomedical Sciences, Texas A&M University, College Station, Texas, United States of America; 2 Clinical Dermatology Service, College of Veterinary Medicine & Biomedical Sciences, Department of Small Animal Clinical Sciences, Texas A&M University, College Station, Texas, United States of America; 3 Gastrointestinal Laboratory, Department of Small Animal Clinical Sciences, College of Veterinary Medicine & Biomedical Sciences, Texas A&M University, College Station, Texas, United States of America; 4 Clinical Microbiology Laboratory, Department of Veterinary Pathobiology, College of Veterinary Medicine & Biomedical Sciences, Texas A&M University, College Station, Texas, United States of America; 5 MR DNA Laboratory, Shallowater, Texas, United States of America; 6 Department of Clinical Sciences, College of Veterinary Medicine, and Center for Comparative Medicine and Translational Research, North Carolina State University, Raleigh, North Carolina, United States of America; Catalan Institute for Water Research (ICRA), Spain

## Abstract

**Background:**

Changes in the microbial populations on the skin of animals have traditionally been evaluated using conventional microbiology techniques. The sequencing of bacterial 16S rRNA genes has revealed that the human skin is inhabited by a highly diverse and variable microbiome that had previously not been demonstrated by culture-based methods. The goals of this study were to describe the microbiome inhabiting different areas of the canine skin, and to compare the skin microbiome of healthy and allergic dogs.

**Methodology/Principal Findings:**

DNA extracted from superficial skin swabs from healthy (n = 12) and allergic dogs (n = 6) from different regions of haired skin and mucosal surfaces were used for 454-pyrosequencing of the 16S rRNA gene. Principal coordinates analysis revealed clustering for the different skin sites across all dogs, with some mucosal sites and the perianal regions clustering separately from the haired skin sites. The rarefaction analysis revealed high individual variability between samples collected from healthy dogs and between the different skin sites. Higher species richness and microbial diversity were observed in the samples from haired skin when compared to mucosal surfaces or mucocutaneous junctions. In all examined regions, the most abundant phylum and family identified in the different regions of skin and mucosal surfaces were Proteobacteria and *Oxalobacteriaceae*. The skin of allergic dogs had lower species richness when compared to the healthy dogs. The allergic dogs had lower proportions of the Betaproteobacteria *Ralstonia* spp. when compared to the healthy dogs.

**Conclusions/Significance:**

The study demonstrates that the skin of dogs is inhabited by much more rich and diverse microbial communities than previously thought using culture-based methods. Our sequence data reveal high individual variability between samples collected from different patients. Differences in species richness was also seen between healthy and allergic dogs, with allergic dogs having lower species richness when compared to healthy dogs.

## Introduction

The human body is colonized by a wide variety of microorganisms, including bacteria, fungi, and viruses [1]. These resident microorganisms live in a symbiotic relationship with their host [2]. However, an imbalance of this microbiome may result in damage to its host. Most of the microorganisms that make up the human skin microbiome have not been cultured or isolated to date. Recent molecular-based methods, most commonly targeting the 16S rRNA gene, have now enabled to characterize these highly complex microbial communities at different sites of the human body. In veterinary medicine, most knowledge on small animal microbiome that is based on 16S rRNA is on the microbial communities present in the gastrointestinal tract [3]. Changes in the microbial populations in the skin of animals have traditionally been evaluated using conventional microbiology techniques such as culture and biochemical methods [4]. The sequencing of bacterial 16S rRNA genes has revealed that the skin surface of humans is inhabited by a highly diverse and variable microbiota that has previously not been demonstrated by culture-based methods [5], [6]. These studies have described the microbial composition in different skin regions, with *Propionibacterium* spp. predominating in sebaceous areas, *Staphylococcus* and *Corynebacterium* spp. predominating in moist areas, and gram-negative organisms (e.g., Betaproteobacteria) colonizing the dry skin areas such as forearm or leg [7]. Furthermore, age was shown to influence the skin microbiome, with infants having a different skin flora than adults. Similar studies in dogs and other animal species are needed to better investigate the role of the skin microbiome in health and disease. There are rare reports on the skin microbiome in dogs. These studies, however, only investigated a few skin sites in a small number of dogs [8], or they were mainly focused on human and dog relationships [9].

The normal skin microbiota is necessary for optimal skin function, modulating the innate immune response and preventing colonization with potentially pathogenic microorganisms [10]. In many skin conditions, it remains unclear if some skin conditions are caused by alterations in the cutaneous microbiome or whether these alterations are a result of the skin disease itself [11]. In humans with atopic dermatitis (AD) and psoriasis, the changes in the cutaneous microbiota have been proposed to be due to different mechanisms, such as an altered epidermal barrier function, Toll-like receptor 2 mutations, reduced levels of antimicrobial peptides, and/or an increased expression of extracellular matrix proteins [12]. These proposed mechanisms are thought to be responsible for an increased prevalence of *Staphylococcus* spp. and susceptibility to staphylococcal infections in human patients with AD [13]. It has also been shown that in humans with AD, infection with *Staphylococcus aureus* correlates with clinical severity of the disease [14]. Furthermore, metagenomics studies have shown that *S. aureus* dominates skin lesions in human patients with AD, although no changes in relative abundance of *S. aureus* are identified in nasal samples [15].

Similar to humans, dogs develop AD with hypersensitivity to environmental allergens such as house dust mites and/or food allergens [16], [17]. AD is considered one of the most common chronic skin diseases in dogs [18], affecting approximately 10% of dogs [19]. In most dogs with AD, primary skin lesions are characterized by intensely pruritic erythematous macules and patches and the most common sites of lesions are the front and hind paws, axilla, and abdomen (inguinal region) [20]. Dogs with AD often suffer from secondary bacterial and/or fungal infections, most commonly due to *Staphylococcus pseudintermedius* [21]; these infections result in an exacerbation of skin lesions with development of papules, pustules, crusts, and alopecia [16].

The primary goal of this study was to evaluate and describe the diversity of the microbiome inhabiting different areas of the canine skin, including mucosal surfaces, mucocutaneous junctions, and haired skin sites. A secondary goal of this study was to compare the skin microbiome of healthy dogs with that of dogs with AD. Similar to studies described in people, we demonstrate that the skin microbiome in dogs is highly variable in the different skin sites evaluated, and that the diversity of the skin microbiome in atopic dogs is reduced when compared to healthy dogs.

## Materials and Methods

### Ethics statement

This study had been approved by the Texas A&M University University (TAMU) Institutional Animal Care and Use Committee. Informed consent to enroll clinical cases into the study was obtained from each client.

### Study subjects (Table 1)

#### Healthy dogs

Twelve healthy dogs were enrolled into this study; their age ranged from 8 months to 13 years old (average 7.5 years old) (Table 1). There were 6 male dogs (1 intact and 5 castrated; 3 Labrador, 1 Boston Terrier, 1 Pug and 1 Blue Heeler) and 6 female dogs (5 spayed and 1 intact; 3 Labrador, 1 Mixed breed, 1 Pitbull and 1 Terrier). Nine dogs were primarily kept indoors, two dogs were kept both indoors and outdoors, and one dog was kept solely outdoors. All dogs co-inhabited with other animals (dogs and/or cats). The healthy study animals had no historical or clinical findings suggestive of allergic skin disease, nor were they treated with antibiotics, anti-inflammatory, or immunosuppressive drugs for at least 6 months prior to sample collection.

**Table 1:** Physical and environmental characteristics of healthy and allergic dogs enrolled in this study.

Dog	Health status	Breed	Age	Sex	Allergy Pruritus*	Ear problems*	Fleas	Time indoors	Outdoor environment	Indoor environment	Allergy treatments
**Dog1**	Healthy	Lab	NA	M	N	N	N	>90%	G	CTL	NA
**Dog2**	Healthy	Lab	6Y	CM	N	N	N	0%	GW	NA	NA
**Dog3**	Healthy	Lab	8mo	CM	N	N	N	70%	TGW	CTFB	NA
**Dog4**	Healthy	Lab	8Y	F	N	N	N	>90%	TGW	NA	NA
**Dog5**	Healthy	Lab	4Y	SF	N	N	N	>90%	TG	TFB	NA
**Dog6**	Healthy	Lab	13Y	SF	N	Y	Y	>90%	TGW	NA	NA
**Dog7**	Healthy	Bos	5Y	CM	N	N	N	80%	TGW	T	NA
**Dog8**	Healthy	Pug	5Y	CM	N	N	N	>90%	TGW	CF	NA
**Dog9**	Healthy	Hee	13Y	CM	N	N	N	40%	TGW	CFB	NA
**Dog10**	Healthy	Mix	11Y	SF	Y	N	Y	85%	TGW	CTFB	NA
**Dog11**	Healthy	Pit	7Y	SF	Y	N	NA	>90%	TGW	NA	NA
**Dog12**	Healthy	Ter	9Y	SF	N	N	Y	>90%	TG	CB	NA
**Dog13**	Allergic	Bos	6Y	CM	Y	N	N	>90%	TG	CTBF	N
**Dog14**	Allergic	Poo	14Y	SF	Y	Y	N	80%	G	CTFB	ASIT
**Dog15**	Allergic	She	4Y	CM	N	N	N	>90%	GW	CTFB	CsA, ASIT
**Dog16**	Allergic	Pit	8Y	CM	Y	Y	N	>90%	G	CTFB	GL
**Dog17**	Allergic	Aus	2Y	CM	Y	N	N	>90%	TGW	C	GL, ASIT
**Dog18**	Allergic	GR	5Y	SF	Y	Y	Y	>90%	NA	CF	N

Pruritus associated with allergy, ear problems and presence of fleas were part of the clinical history of these canine patients, and not necessarily the clinical presentation at the time of sample collection. Lab: Labrador Retriver, Bos: Boston Terrier, Hee: Blue Heeler, Mix: Mixed breed, Pit: Pitbull, Ter: Terrier, Poo: Poodle, She: Shetland Sheepdog, Aus: Australian Shepherd, GR: Golden Retriever, NA: Not available, M: male, CM: castrated male, F: female, SF: spayed female, N: no, Y: yes, T: trees, G: grass, W: weeds, C: carpet, T: tile, L: leather, B: bedding, F: furniture, G: glucocorticoid, CsA: cyclosporine, ASIT: allergen-specific immunotherapy.

#### Allergic dogs

Six dogs ranging from 2 to 14 years (average 6.5 years old) with allergic skin diseases were also enrolled into this study (Table 1). They were all purebred (1 Boston Terrier, 1 Poodle, 1 Shetland Sheepdog, 1 Pitbull, 1 Australian Shepherd and 1 Golden Retriever), 4 were castrated males and 2 were spayed females. Five dogs were diagnosed with AD using standard diagnostic and therapeutic methods including fulfillment of at least five of Favrot's criteria and exclusion of other pruritic dermatoses (e.g., sarcoptic acariasis, flea allergy dermatitis, and cutaneous adverse food reactions) [22]. One dog was diagnosed with atopic-like dermatitis, it exhibited signs of AD but IgE-mediated hypersensitivity to environmental allergens could not be demonstrated by either allergen-specific intradermal or serological testing. Allergic dogs were primarily kept indoors, but did receive monthly adulticidal flea prevention. All dogs co-inhabited with other animals (dogs and/or cats). To be included, dogs could not display overt clinical signs of bacterial skin infection or *Malassezia* dermatitis, nor could they have received systemic antibiotics for at least 30 days or have a bath for at least 7 days prior to sample collection. Three dogs were receiving anti-inflammatory doses of glucocorticoids (alternate day administration) or the immunomodulatory drug cyclosporine (modified), and three were being treated with allergen-specific immunotherapy (ASIT). Two of the allergic dogs (dogs 13 and 18) had not received glucocorticoids within 6 months prior to sample collection, cyclosporine (dog 13 received cyclosporine 4 years prior to the study), or ASIT. Although dogs had no skin lesions, most exhibited mild to moderate pruritus at the time of the study.

### Sample collection

Samples were collected from 12 skin sites from 12 healthy dogs, for a total of 144 samples. The skin sites included the right nasal mucosa, right dorsal nose, right lip commissure, right conjunctiva, right periocular area, right ear canal, right concave pinna, dorsal lumbar area, right axilla, right groin, right dorsal interdigital skin between digits 4 and 5 from the right front paw, and dorsal perianal area. Samples were collected from 4 skin sites from 6 allergic dogs for a total of 24 samples. Sites included the right axilla, right groin, right nasal mucosa, and right dorsal interdigital skin between digits 4 and 5 from the right front paw.

For each skin site, two sterile culture swab applicators (BD Biosciences, NJ) were used. Each swab applicator was rubbed on the skin 40 times, while rotating each swab by one quarter for every 10 strokes. The two swabs were stored in the same properly labeled tube and refrigerated at 4°C until further analysis.

### DNA extraction and pyrosequencing

Genomic DNA was extracted from each set of sterile swabs collected from each skin site using the Mobio Power soil DNA isolation Kit (MoBio Laboratories), as recommended by the manufacturer. Bacterial tag-encoded FLX-titanium amplicon pyrosequencing (bTEFAP) based upon the V1–V3 region (*E. coli* position 27–519) of the 16S rRNA gene was performed at the MR DNA Laboratory, Shallowater, TX, USA, as described previously, with primers forward 28F: GAGTTTGATCNTGGCTCAG and reverse 519R: GTNTTACNGCGGCKGCTG [23]. Raw sequence data were screened, trimmed, filtered, denoised, and chimera depleted with default settings using the softwares QIIME pipeline version 1.6 (http://qiime.sourceforge.net) [24], and UCHIME (http://www.drive5.com/uchime/) [25]. Operational taxonomic units were defined as bacterial sequences with at least 97% similarity using QIIME. The sequences obtained in this study have been deposited in the NCBI Short Read Archive accession number SRP028524.

### Data analysis

A total of 779,812 sequences were amplified from all skin samples from the healthy and allergic dogs. A mean of 4,754 sequences (median 3,450 sequences) were obtained per sample from each skin site, with a minimum of 195 sequences and a maximum of 55,956 sequences per site. Due to unequal sequencing depth between the different sites and samples, and to standardize sequence counts across samples, data analysis was performed on a randomly selected subset of 1,000 sequences per sample. One hundred and thirty samples from the healthy dogs and 17 samples from the allergic dogs had more than 1,000 sequences, and were considered for data analysis. All samples with less than 1,000 sequences per sample were removed from further analysis. Alpha diversity [i.e., rarefaction; the number of different species (species richness) per sample], and beta diversity (i.e. microbial communities similarity) measures were calculated and plotted using the software QIIME v1.6. On samples from the healthy dogs that had higher numbers of sequences, rarefaction was also performed on a randomly selected subset of 3,000 sequences, to evaluate species richness at higher sequencing depth (number of times a specific genomic site is sequenced in a sequencing run), and a total of 89 samples were evaluated. The phylogeny-based UniFrac distance metric analysis was used to investigate differences in microbial communities between skin sites, as well as between groups (healthy vs. allergic) [26]. Both the weighted, which accounts for relative abundance of sequences in different environments, and unweighted, which does not account for relative abundance, UniFrac analysis were performed.

The analysis of similarities (ANOSIM) function in the statistical software package PRIMER 6 (PRIMER-E Ltd., Luton, UK) was used on the weighted and unweighted UniFrac distance matrix to determine if any groups of samples contained significantly different bacterial communities. Because of adjustment for multiple comparisons, p-values equal or above 0.001 were considered for significance. The R values of the statistical test ANOSIM provide an estimate of the effect size and range from 1 to −1. R values closer to 0 indicate that no differences exist between the different skin sites, whereas values closer to 1 indicate that differences between skin sites exist. Differences in the proportions of bacterial taxa (percentage of total sequences) between the different sites, and between healthy and allergic dogs were tested for normality, and since data was not normally distributed, a non-parametric Kruskal-Wallis test was performed, using the statistical package JMP10 (SAS, Marlow, Buckinghamshire). Resulting *p*-values were corrected for multiple comparisons using the Benjamini & Hochberg's False Discovery Rate [27]. An adjusted *p*<0.05 was considered for statistical significance.

## Results

### Skin microbiome of healthy dogs

#### Skin microbial composition of healthy dogs

Similarities in microbial community composition between samples were evaluated using the unweighted and weighted UniFrac distance metrics. The unweighted UniFrac metric was significantly different using ANOSIM analysis, when mucosal surfaces and mucocutaneous zones where compared to haired skin sites (Table 2). However, when the weighted UniFrac metric was considered, which gives emphasis to abundance of operational taxonomic units (bacterial species), fewer sites were considered to be significantly different.

**Table 2:** ANOSIM analysis of unweighted and weighted Unifrac distances.

Skin sites	Conjunctiva		Dorsal perianal		Lip commissure		Nostril	
	R (unwtd)	R (wtd)	R (unwtd)	R (wtd)	R (unwtd)	R (wtd)	R (unwtd)	R (wtd)
**Axilla**	0.364*	0.302*	0.672	0.389*	0.618*	0.367	0.63*	0.503*
**Conjunctiva**	-	-	0.465*	0.305*	0.491*	0.302	0.146	0.071
**Concave Pinna**	0.283	0.227	0.684*	0.284	0.49*	0.252	0.524	0.383
**Dorsal Lumbar**	−0.003	−0.047	0.353	0.272	0.471*	0.306	0.256	0.126
**Dorsal nose**	0.391*	0.269	0.69*	0.333*	0.507*	0.288*	0.592*	0.43*
**Dorsal perianal**	0.465*	0.305*	-	-	0.564*	0.097	0.48*	0.285
**Ear**	0.118	0.069	0.518*	0.355*	0.526*	0.384*	0.418*	0.301
**Groin**	0.187	0.073	0.332	0.135	0.248*	0.098	0.332	0.183
**Interdigital**	0.136	0.091	0.564*	0.259	0.549*	0.309	0.419	0.217
**Lip commissure**	0.491*	0.302	0.564*	0.097	-	-	0.366*	0.224
**Nostril**	0.146	0.071	0.48*	0.285	0.366*	0.224	-	-
**Periocular**	−0.002	0.14	0.503*	0.217	0.376	0.262	0.233	0.234

R-values are shown for the healthy skin sites that showed significant differences. R- values closer zero to represent no difference between different sites, whereas values closer to 1 indicate that the most similar samples are within the same group.

Significance level p = 0.001.

**Figure 1:**
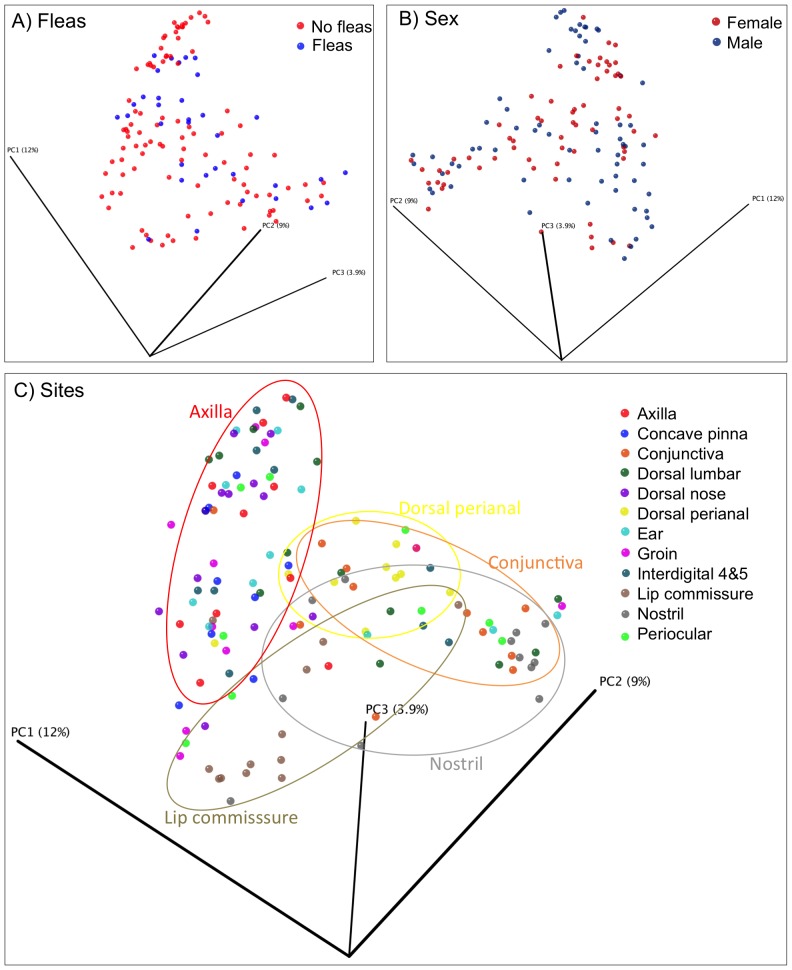
Principal coordinates analysis for healthy dogs. Principal coordinates analysis of unweighted Unifrac distances of 16S rRNA genes clusters samples based on similarities of bacterial molecular phylogenetic trees. (A) No clustering differences are observed in 3 healthy dogs with fleas compared to 9 healthy dogs without fleas, demonstrating that the presence of fleas does not appear to influence the microbial diversity. (B) Similarly, there were no clustering differences between male and female dogs. (C) Clustering differences were seen in the samples collected from mucosal surfaces or mucocutaneous junctions.

Principal coordinates analysis plots were constructed using the unweighted UniFrac metric to evaluate similarities of microbial communities when considering individual (signalment, pruritus associated with allergic skin disease, ear problems, and presence of fleas) and environmental (time spent indoors and type of immediate environment) characteristics in the healthy dogs. A clustering, based on similarities of bacterial molecular phylogenetic trees, was not observed between healthy dogs when breed, age, sex, presence of fleas, housing habits, indoor and outdoor environments were compared between the different samples (Figures 1A and 1B). A large degree of variability was seen across all samples from the different skin sites from each dog, and from samples from the same site across all dogs. Clustering was only seen for the different skin sites across all dogs, with some mucosal sites and the perianal regions, clustering separately from the haired skin sites (Figure 1C; Table 2; ANOSIM p = 0.001).

#### Species richness and diversity within skin samples of healthy dogs

**Figure 2:**
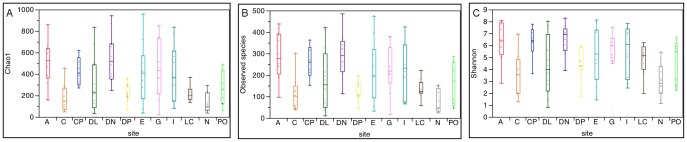
Alpha diversity in different skin sites for healthy dogs. Alpha diversity measures at 1000 sequences per sample in the different sites of canine skin (*x* axis). The *y* axis represent the data points for the Chao1 index (species predictor estimator) (A), number of observed species (B) and Shannon diversity index (diversity index that accounts for species abundance and evenness) (C) data points (*y* axis) for each skin site. Error bars represent the standard deviations. A: Axilla; C: Conjunctiva; CP: Concave pinna, DL: Dorsal lumbar; DN: Dorsal Nose; DP: Dorsal Perianal; E: Ear; G: Groin; I: Interdigital 4&5; LP: Lip commissure; N: Nostril; PO: Periocular.

**Figure 3:**
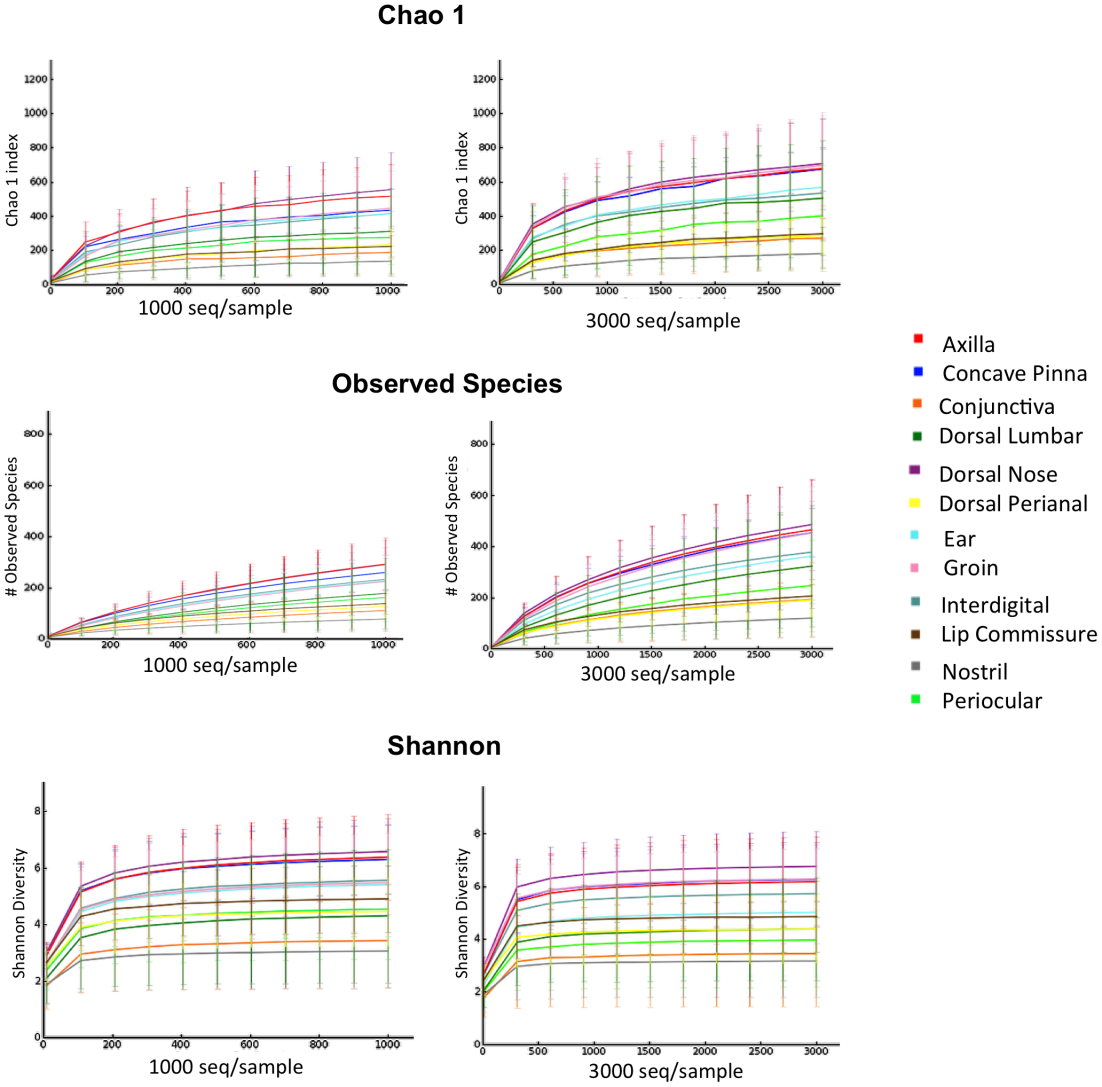
Rarefaction curves from different skin sites from healthy dogs. Rarefaction curves of 16S rRNA gene sequences obtained from different skin sites from healthy dogs. The analysis was performed on a randomly selected subset of 1000 and 3000 sequences per sample. Haired skin shows higher Chao1 metric, more observed species, and higher Shannon index compared to the samples from mucosal surfaces, e.g. nostril and conjunctiva. Lines represent average of each skin site, whereas the error bars represent the standard deviations.

The rarefaction analysis, which evaluates species richness in the samples, revealed high individual variability between samples collected from healthy dogs and between the different skin sites. Higher species richness, evaluated using the number of observed species, was observed in the samples from haired skin (i.e., axilla, concave pinna, dorsal nose, and groin) when compared to mucosal surfaces or mucocutaneous junctions (i.e., nostril and conjunctiva; Figure 2; Table 3). The samples from the nostril and conjunctiva had the lowest species richness; whereas the samples collected from the axilla and the haired skin from the dorsal aspect of the nose had higher species abundance. When 1,000 and 3,000 sequences per sample were analyzed, the number of observed species ranged from 25 and 41 in the nostril, to 486 and 833 in the dorsal nose, respectively (Figure 3). One sample from the ear had 866 observed species, and was the sample with the largest number of observed species in the analysis performed on 3,000 sequences per sample. The Chao 1 index, which is an estimator for species richness at higher sequencing depth, gave similar results for the different skin sites evaluated, with most mucosal surfaces having lower Chao 1 index and the haired skin sites having higher Chao 1 index (Figures 2 and 3; Table 3).

**Table 3:** Alpha diversity measures at 1000 sequences per sample in the different sites of healthy skin.

Skin site	Chao 1	Observed species	Shannon
	Median (Min-Max)	Median (Min-Max)	Median (Min-Max)
**Axilla**	530 (163–859)	277 (97–440)	6 (3–8)
**Conjunctiva**	151^A,N^ (51–457)	104^A,N^ (38–302)	4^A,N^ (1–7)
**Concave pinna**	412 (272–620)	260 (153–364)	6 (4–8)
**Dorsal lumbar**	225 (33–836)	156 (12–423)	4 (1–8)
**Dorsal nose**	525 (249–945)	291 (115–486)	7 (4–8)
**Dorsal perianal**	218 (64–359)	109 (45–196)	4 (2–6)
**Ear**	409 (41–961)	196 (32–476)	5 (1–8)
**Groin**	433 (21–851)	219 (16–380)	6 (1–8)
**Interdigital**	364 (85–839)	234 (66–425)	6 (2–8)
**Lip comissure**	205 (140–371)	126 (57–223)	5 (2–6)
**Nostril**	101^A,CP,DN,G^(39–296)	47^A,CP,DN^ (25–154)	3^A,CP,DN^ (1–5)
**Periocular**	258 (60–492)	172 (45–287)	6 (2–7)

Significant differences between skin sites were mainly observed when comparing mucosal surfaces with haired skin sites, e.g. conjunctiva versus axilla. The Chao 1 index estimates species richness at higher sequencing depth; the observed species represents the number of observed species in 1,000 sequences; the Shannon is a diversity index that takes into account abundance and evenness of species.

Superscripts represent sites that were significantly different when compared to the skin sites in the first column. A: Axilla; N: Nostril; CP: Concave pinna; DN: Dorsal nose; G: Groin.

The Shannon diversity index (Figures 2 and 3; Table 3), which takes into account abundance and evenness of species, showed similar results to those observed with the Chao 1 index and the number of observed species. Similarly, mucosal sites, including the nostril and conjunctiva, were less diverse, with lower Shannon index, when compared to haired skin sites, e.g., axilla, concave pinna, and dorsal nose, which presented with higher Shannon index.

#### Most common taxa colonizing the skin of healthy dogs

**Figure 4:**
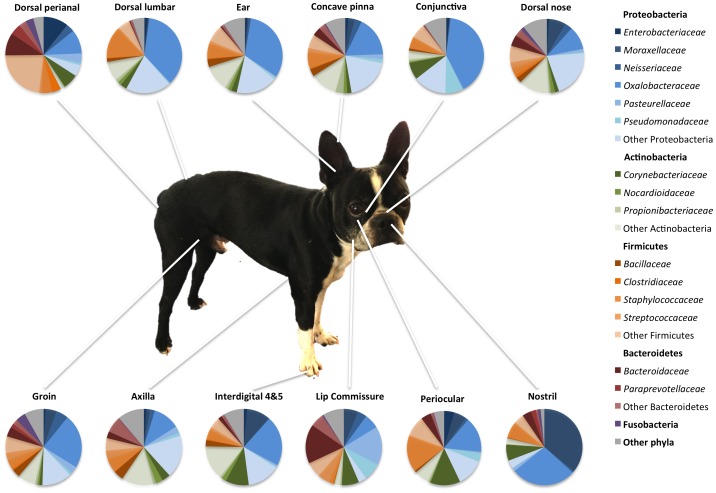
Bacterial phyla and families in healthy dogs. Average of most common bacterial phyla and families identified in different sites in the skin of healthy dogs.

A total of seventeen phyla were identified in the samples from skin and mucosal surfaces (Table S1). In all examined regions, the most abundant phylum identified in the different regions of skin and mucosal surfaces was Proteobacteria (Figure 4). This was followed by Firmicutes, Actinobacteria, Bacteroidetes, and Cyanobacteria. However, in the samples collected from axilla, concave pinna, dorsal nose, and interdigital skin, Proteobacteria were followed by Bacteroidetes and Actinobacteria. The samples from the perianal skin were slightly different, with Proteobacteria being followed by Firmicutes, Bacteroidetes, Fusobacteria, and Actinobacteria.

At the class level, more variability was observed between the different sites, with Betaproteobacteria being the most common class identified in the concave pinna, conjunctiva, dorsal lumbar, ear, and groin; whereas Actinobacteria were most common in the axilla and interdigital skin; Gammaproteobacteria in the lip commissure and nostril; Alphaproteobacteria in the dorsal nose, and Bacilli in the periocular region. The Clostridia and Bacteroidia were the most common classes in the perianal region, as would be expected due to the close proximity to the rectum.

The family *Oxalobacteriaceae* (phylum Proteobacteria; class Betaproteobacteria; Order Burkholderiales) was the most abundant group in most samples. The genus *Ralstonia* spp. was the most abundant genus identified in most samples, ranging from an average of 5% of the total taxa identified in the lip commissure to 35% of the taxa identified in the conjunctiva. The family *Moraxellaceae* was significantly more abundant in the nostril compared to other sites (median 33.1%; *p*-value<0.0001; q-value = 0.0001). The lip commissure was predominantly colonized by the family *Porphyromonadaceae* and genus *Porphyromonas* spp. (median 7.95%; *p*-value = 0.0006; q-value<0.001). Other genera that were commonly present in most samples of skin and mucocutanous junctions included *Bacillus* spp., *Corynebacterium* spp., *Macrococcus spp*., and *Pseudomonas* spp.

### Skin microbiome of healthy versus allergic dogs

#### Microbial community composition in allergic versus healthy dogs

To compare microbial communities between samples from allergic versus healthy dogs, the statistical analysis ANOSIM was performed on the unweighted and weighted UniFrac distances. Significant differences were not noted in microbial community composition between allergic and healthy dogs.

**Figure 5:**
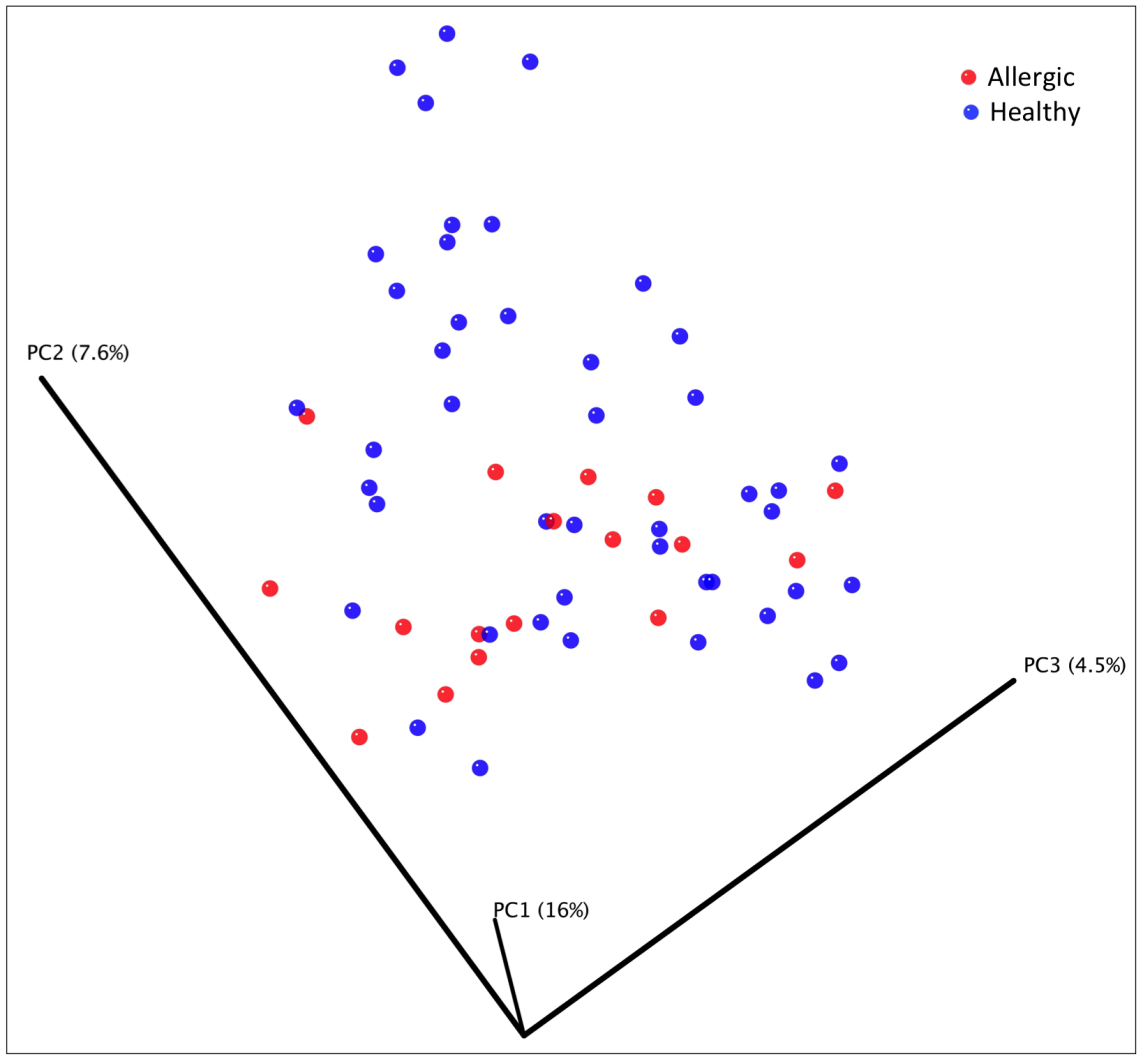
Principal coordinates analysis for allergic versus healthy dogs. Principal coordinates analysis plot of unweighted Unifrac distances of 16S rRNA genes. No clustering differences are observed between allergic versus healthy dogs in the samples from the nostril, axilla, groin and interdigital skin.

Principal coordinate analysis plots from unweighted UniFrac metric were constructed to evaluate similarities between individual (breed, age, sex, pruritus associated with allergic skin disease, ear problems, and presence of fleas) and environmental (time spent indoors and type of immediate environment) factors. Principal coordinate analysis plots were also constructed to evaluate similarities between the different samples from allergic and healthy dogs. No significant clustering was noted in the PCoA plots between allergic and healthy dogs (Figure 5).

#### Species richness and diversity in allergic versus healthy dogs

**Figure 6:**
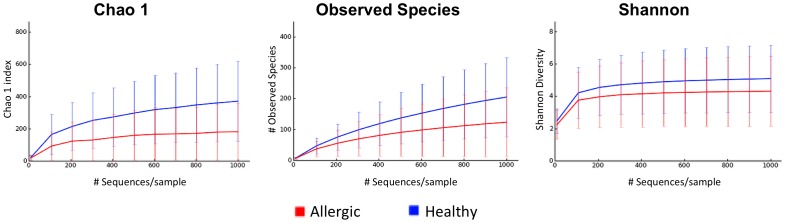
Rarefaction curves of 16S rRNA gene from allergic versus healthy dogs. Rarefaction curve of 16S rRNA gene sequences obtained from axilla, groin, interdigital skin and nostril mucosa from allergic and healthy dogs. Lines represent average of each group, whereas the error bars represent the standard deviations. The analysis was performed on a randomly selected subset of 1000 sequences per sample.

Diversity analysis performed in a randomly selected 1,000 sequences per sample showed that the samples from the haired skin of dogs with allergic skin disease (median 125) showed a lower number of observed bacterial species when compared to the same skin sites (axilla, groin, and interdigital skin) of healthy dogs (median 239; *p*<0.016; Table 4). Significant differences in the haired skin and nostril mucosa of healthy versus allergic dogs were also identified for the Chao1 metric (species richness estimator at higher sequencing depth; *p*<0.005; Table 4, Figure 6). Although the median for the Shannon diversity index, which considers abundance and evenness of species, was lower for the allergic dogs when compared to the healthy dogs, the difference was not significant (p = 0.24).

**Table 4:** Alpha diversity analysis of the nostril mucosa and haired skin including axilla, groin and interdigital area of healthy vs allergic dogs.

Skin site	Healthy status	Chao 1	Observed species	Shannon
		Median (Min–Max)	Median (Min–Max)	Median (Min–Max)
Nostril mucosa	Healthy	100 (39–296)	47 (25–154)	2.85 (1.14–5.44)
	Allergic	40* (26–45)	31 (21–39)	1.54 (1.04–3.89)
Haired skin	Healthy	432 (21–858)	239 (16–440)	6.01 (0.88–8.09)
	Allergic	168* (27–585)	125* (23–371)	5.40 (1.14–7.82)

Significant differences between healthy versus allergic (*p*<0.05).

#### Most common taxa colonizing the skin of allergic versus healthy dogs

**Figure 7:**
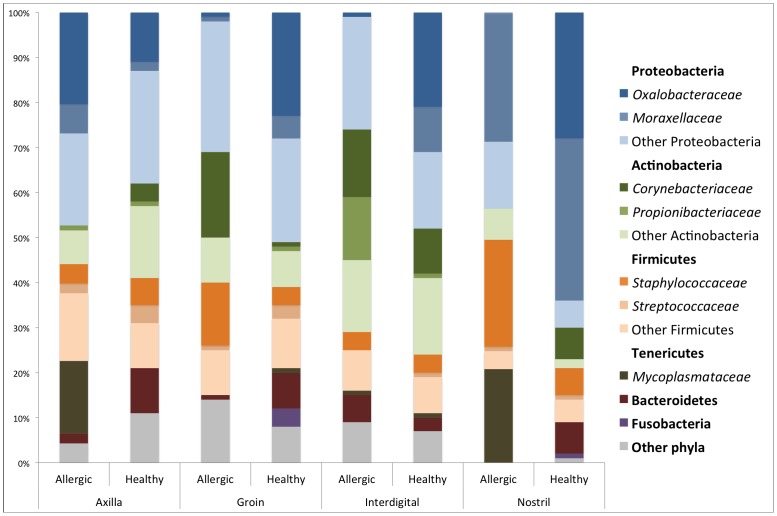
Bacterial phyla and families in allergic versus healthy dogs Average of most common bacterial phyla and families identified in axilla, groin, interdigital skin and nostril in allergic and healthy dogs.

Similar abundances of the most common bacterial taxa observed in the healthy dogs were also identified in the samples from allergic dogs (Figure 7). However, taxa that were minimally represented in the healthy dogs (<1%) were often absent in allergic dogs (Table S2). Significant differences between allergic and healthy dogs were identified for a few taxa. One major difference between allergic and healthy dogs was the proportions of the Beta Proteobacteria *Ralstonia* spp., which were significantly lower in the samples from the allergic dogs (p-value = 0.0001; q-value = 0.0001). In fact, *Ralstonia* spp. accounted for less than 0.02% of the total taxa identified in the samples from the allergic dogs, with the exception of one sample from the axilla, where it accounted for 45% of the total taxa identified.

In the samples from the axilla from the allergic dogs, some of the most predominant genera were *Bacillus* spp. (median 3.8%), *Sphingomonas* spp. (median 3.1%), *Mycoplasma* spp. (median 2.4%), *Rubellimicrobium* spp. (median 1.4%) and *Propionibacterium* spp. (median 1.3%). The samples from the groin were predominantly colonized by *Staphylococcus* spp. (median 2.6%), *Sphingomonas* spp. (median 2.4%), *Bacillus* spp. (median 1.8%), and *Roseomonas* spp. (mediam 1.4%). The interdigital skin was predominantly colonized by *Alicyclobacillus* spp. (median 1.6%), *Staphylococcus* spp. (median 1%), *Pseudomonas* spp. (median 1%) and *Corynebacterium* spp. (median 0.7%). The samples from the nostril were predominantly colonized by *Streptococcus* spp. (median 0.5%), *Diaphrobacter* spp. (median 0.25%) and *Sphingomonas* spp. (median 0.1%). At the family level, similar to the samples from the healthy dogs, the nostril was predominantly colonized by *Moraxellaceae* (median 8%) (Table S2).

## Discussion

In this study, we demonstrate that the skin microbiome in dogs is much more diverse than has been previously reported based on culture-based methods. Our sequence data reveal a high individual variability between samples collected from different patients. High variability was also observed between different skin regions within the same dogs, with a higher number of bacterial species being observed on the haired skin (i.e., axilla, groin, periocular, pinna, dorsal nose, interdigital, lumbar) when compared to poorly haired skin, mucocutaneous junctions, or mucosal surfaces (i.e., lips, nose, and conjunctiva). Although this is still a preliminary study and additional samples from dogs are needed in order to make any further conclusions, the results suggest that the composition of the bacterial community in the dogs evaluated were not influenced by the individual factors tested (e.g., age, sex, breed, pruritus, ear problems) or by environmental factors (e.g., fleas, indoor/outdoor environment, time spent outdoors) in the skin samples of healthy and allergic dogs. However, we did not evaluate if antipruritic pharmacotherapy or allergen specific immunotherapy could have had an effect on the composition of the microbiota because of the small number of test subjects.

In previous studies using culture-based methods, some of the most common commensal bacteria in the skin of healthy dogs were shown to include *Micrococcus* spp., coagulase-negative staphylococci – mainly *Staphylococcus epidermidis* and *S. xylosus*–, α-hemolytic streptococci, *Clostridium* spp., *Propionibacterium acnes*, *Acinetobacter* spp., and various Gram-negative aerobes [28]–[30]. *Escherichia coli, Proteus mirabilis, Corynebacterium* spp., *Bacillus* spp., and *Pseudomonas* spp. were considered to be transient microbes in the skin of dogs [30]. In contrast, *Micrococcus* spp., Gram-negative aerobes, *Bacillus* spp., and *Staphylococcus pseudintermedius* (formerly known as *Staphylococcus intermedius*) [31] were reported to be the most common microbes isolated from canine hair and hair follicles by other authors [28]. *S. pseudintermedius* is frequently cultured from the nostril, oropharynx, and perianal region, and is being considered part of the resident flora in these regions, representing the main source of coagulase-positive *Staphylococcus* in the mucous membranes [32], [33]. In this study, we identified microbes similar to those described with prior culture-based methods. In addition to these microbes, a large number of previously uncultured or rarely isolated bacteria were identified in this study, thereby demonstrating that the skin of dogs is colonized by much richer and diverse microbial communities than was previously thought.

Members of the genus *Ralstonia* are Gram-negative bacteria considered to be primarily environmental organisms found in water, soil, and plants, with only a few members being considered pathogenic [34]. Different species of *Ralstonia* have been occasionally isolated from the airways of human patients with cystic fibrosis, although the significance of this observation remains unclear [35]. The significance of the abundance of *Ralstonia* spp. in the samples from healthy dogs and its absence in the samples from the dogs with allergic skin disease is unknown at this point. It is very likely that the *Ralstonia* spp. identified in these samples were obtained from the environment, given a dog's frequent interactions with its outdoor's environment. Previous reports have found *Ralstonia* spp., along with other bacterial genera, to be water contaminants in reagents used for qPCR [36]. Although we cannot completely exclude this possibility, *Ralstonia* spp. were not isolated from negative controls run along the tested samples. Moreover, the DNA from all samples from healthy and allergic dogs were extracted using the same reagents, and it would be unlikely that a significantly lower abundance of this genus would be found only in the samples from allergic dogs.

The family *Moraxellaceae* was frequently identified in the samples from the nostril from the healthy dogs in this study. Using the QIIME database, most sequences in this family were not assigned to any specific genus, however when these were compared to sequences in the NCBI Basic Local Alignment Search Tool database [37], they usually exhibited a 97–100% identity to sequences of *Moraxella catarrhalis*. The genus *Moraxella* has been previously isolated from oral swabs from healthy dogs [38] and from bronchial samples from dogs with tracheal collapse [39]. A recent metagenomics study evaluating the oral cavity of healthy dogs also frequently identified the genus *Moraxella* in evaluated samples [40]. In cattle, *Moraxella bovis*, the cause of bovine keratoconjunctivitis, is frequently isolated from nasal and ocular secretions [41].

A previous study using samples from children with AD reported a lower microbial diversity during flares of AD compared to baseline and post-flare samples.[15] In our study, the skin samples from allergic dogs in this study similarly showed a lower diversity when compared to the samples from healthy dogs. Since the samples collected from the allergic dogs were “baseline” samples (non flares), we speculate that the lower diversity in the skin of allergic dogs is possibly a result of frequent antimicrobial treatments, although none of the allergic dogs in this study had been treated with antimicrobials for at least one month. Previous studies have also shown that the visibly normal (i.e. nonlesional) skin of dogs with AD is not normal (i.e. it is more inflamed than normal skin), and this inflammation could lead to skin surface changes leading to lower bacterial diversity [42], [43].

Using culture-based methods, the skin and nasal mucous membranes of atopic human patients [15], [44] and dogs [21], [45] are more often colonized with *S. aureus* and *S. pseudintermedius*, respectively, than healthy patients. Based on 16S rRNA pyrosequencing data, *S. aureus* markedly dominated affected skin regions, more commonly the antecubital and popliteal creases, in children with AD. Likewise, baseline and post flare samples from children with AD also had more abundance of *S. aureus* compared to the skin of healthy children [15]. *Staphylococcus* spp. was also frequently identified in the skin of allergic dogs in this study. Although not significantly different, the proportions of *Staphylococcus* spp. were higher in the skin of (post-flare) allergic than in healthy dogs.

## Conclusions

A large number of previously uncultured or rarely isolated microbes were identified in the skin of dogs evaluated in this study, demonstrating that the skin of dogs is inhabited by much more rich and diverse microbial communities than was previously thought, using culture-based methods. The study also shows that each skin site from each dog evaluated here was inhabited by a variable and unique microbiome, with significant individual variability between samples from different dogs and between different skin sites within the same dog. Differences in species richness were also seen between healthy and allergic dogs, with allergic dogs having significantly lower species richness when compared to healthy dogs. Since the number of allergic dogs enrolled into this study was small, and significant variability was observed between individuals and between different skin sites, a larger cohort of healthy and allergic dogs would have to be evaluated before drawing any further conclusions on the most important microbes inhabiting the skin of dogs, and the roles that these microbes play in health or disease states. A study of the skin microbiome of allergic dogs during acute flares and chronic skin lesions might also confirm a lowering of this bacterial diversity.

It is imperative for us to better understand the microbial populations inhabiting the skin of animals. Being able to describe the skin microbiome in healthy animals, and identify the changes that occur in the skin microbiome in disease states, could reveal the role of the microbiome in the pathogenesis of skin diseases, and possibly identify better measures to treat skin conditions, ultimately reducing usage and resistance to systemic antimicrobial treatment.

## Supporting Information

Table S1 **Relative percentages of the most abundant bacterial groups on the different skin sites in the healthy dogs at the various phylogenetic levels (phylum, class, order, family, genus) based on pyrosequencing.**
(PDF)

Table S2 **Relative percentages of the most abundant bacterial groups on the different skin sites in the allergic versus healthy dogs at the various phylogenetic levels (phylum, class, order, family, genus) based on pyrosequencing.**
(PDF)
