# Supplementary material for: The Skin Microbiome in Healthy and Allergic Dogs
Source: PLoS One. 2014 Jan 8;9(1):e83197. doi: 10.1371/journal.pone.0083197 (PMC3885435; doi:10.1371/journal.pone.0083197)
Supplement: Table S1 — Relative percentages of the most abundant bacterial groups on the different skin sites in the healthy dogs at the various phylogenetic levels (phylum, class, order, family, genus) based on pyrosequencing. (PDF) [file pone.0083197.s001.pdf]

Table S1. Relative percentages of the most abundant bacterial groups on the different skin sites in the healthy dogs at the various phylogenetic levels (phylum, class, order, family, genus) based on pyrosequencing.

Taxon	p-value	q-value*	Median % (Min-Max%)											
			Axilla	Concave Pinna	Conjuctiva	Dorsal Lumbar	Dorsal Nose	Dorsal Perianal	Ear	Groin	Interdigit45	Lip Commissure	Nostril	Periocular
Acidobacteria	0.0153	0.0357	0.45 (0 - 5.5)	0.1 (0 - 2.1)	0.1 (0 - 2.3)	0.25 (0 - 2.7)	0.55 (0 - 2.7)	0.05 (0 - 2.2)	0.2 (0 - 2.5)	0 (0 - 5.7)	0.45 (0 - 3.1)	0 (0 - 0.9)	0 (0 - 0.3)	0.1 (0 - 2)
Chloracidobacteria	0.0458	0.17011429	0.1 (0-2.4)	0.1 (0-0.8)	0 (0-1.6)	0.05 (0-1.9)	0.2 (0-1)	0.05 (0-0.2)	0.1 (0-0.9)	0 (0-1.7)	0 (0-1.7)	0 (0-0.7)	0 (0-0.2)	0 (0-0.7)
Actinobacteria	0.0016	0.00186667	20.95 (1.5 - 51.5)	18.1 (6.6 - 30)	3.7 (0.2 - 61)	11.5 (0 - 40.2)	15.15 (3.3 - 44.9)	2.7 (0.3 - 60.9)	16.4 (0.6 - 36.4)	8.1 (0.2 - 38.3)	21.25 (1 - 94)	5.15 (1 - 65.4)	2.05 (0 - 78.5)	11 (2.5 - 97.1)
Acidimicrobiia	0.0036	0.00624	0 (0-0.7)	0.1 (0-0.7)	0 (0-1.2)	0 (0-0.9)	0.1 (0-0.7)	0 (0-0.1)	0.1 (0-0.8)	0 (0-0.4)	0.05 (0-1)	0^{E} (0-0)	0^{E} (0-0)	0.1 (0-0.4)
Acidimicrobiales	0.0036	0.00952941	0 (0-0.7)	0.1 (0-0.7)	0 (0-1.2)	0 (0-0.9)	0.1 (0-0.7)	0 (0-0.1)	0.1 (0-0.8)	0 (0-0.4)	0.05 (0-1)	0^{E} (0-0)	0^{E} (0-0)	0.1 (0-0.4)
Actinobacteria	0.0021	0.00321176	15.1 (1.5-48.1)	15.3 (6.4-24.2)	3.6 (0.1-59.2)	10 (0-25.2)	12.75 (2.7-27.5)	2.3 (0.3-60.5)	14.7 (0.6-24.4)	7.8 (0.2-34.1)	14.2 (0.5-93.5)	5.15 (1-65.2)	$1.45^{A, B}$ (0-78.5)	8.7 (1.3-96.9)
Actinomycetales	0.0023	0.00544737	14.9 (1.5-48.1)	15.1 (6.4-24.2)	3.6 (0.1-59.2)	10 (0-25.2)	12.55 (2.6-26.7)	2.3 (0.3-60.5)	14.4 (0.6-24.1)	7.8 (0.2-34)	14.1 (0.5-93.5)	5.15 (1-65.2)	1.45^{A} (0-78.5)	8.7 (1.3-96.9)
Actinomycetaceae	0.0166	0.06989474	0.05 (0-1)	0.1 (0-1)	0 (0-0)	0 (0-0.2)	0.1 (0-1)	0 (0-0.5)	0 (0-1.8)	0 (0-0.4)	0 (0-0.4)	0.2^{C} (0-1.8)	0 (0-3.6)	0 (0-0.6)
Actinomyces	0.0043	0.00759362	0 (0-0.2)	0 (0-0.2)	0 (0-0)	0 (0-0.1)	0 (0-0.4)	0 (0-0.2)	0 (0-0.2)	0 (0-0.2)	0 (0-0.2)	$0.1^{C, D, G}$ (0-0.9)	0^{I} (0-3.6)	0 (0-0.6)
Corynebacteriaceae	0.5475	43.8	0.85 (0-31)	1.8 (0.2-5.3)	0.7 (0-55.2)	0.2 (0-24)	0.95 (0.2-8.4)	1.45 (0-52.2)	0.4 (0-13.5)	0.9 (0-5.2)	0.25 (0-91.3)	1.4 (0-63)	0.15 (0-77.6)	1.4 (0-96.1)
Corynebacterium	0.5533	22.96195	0.85 (0-31)	1.8 (0.2-5.3)	0.7 (0-55.2)	0.2 (0-24)	0.95 (0.2-8.4)	1.4 (0-52.2)	0.4 (0-13.5)	0.9 (0-5.2)	0.25 (0-91.3)	1.4 (0-63)	0.15 (0-77.6)	1.4 (0-96.1)
Frankiaceae	0.0476	0.31733333	0.05 (0-0.3)	0 (0-0.6)	0 (0-0.8)	0 (0-0.3)	0 (0-0.4)	0 (0-0)	0 (0-1.2)	0 (0-0.2)	0 (0-0.3)	0 (0-0.1)	0 (0-0)	0 (0-0.6)
Geodermatophilaceae	0.0008	0.0013617	0.05 (0-0.5)	0.2 (0-1)	0 (0-0.7)	0 (0-2.5)	0.2 (0-0.9)	0 (0-0.1)	0 (0-1.1)	0 (0-0.4)	0.25 (0-0.7)	$0^{E, I}$ (0-0.1)	$0^{E, I}$ (0-0.2)	0 (0-0.6)
Intrasporangiaceae	0.0004	0.00059259	0.35 (0-4.5)	0.4 (0-1.2)	0.1 (0-0.5)	0 (0-2.9)	0.3 (0-2)	0 (0-0.3)	0.2 (0-0.6)	0.1 (0-2.1)	0.25 (0-1.1)	0 (0-0.8)	$0^{A, B, E, I}$ (0-0)	0.1 (0-1.5)
Kineosporiaceae	0.0001	0.0001	0.1 (0-1.1)	0.2 (0-0.6)	0^{B} (0-0.6)	0 (0-0.3)	0 (0-1.4)	0^{B} (0-0.2)	0.1 (0-1.3)	0^{B} (0-0.2)	0.05 (0-0.4)	0^{B} (0-0)	0^{B} (0-0)	0 (0-0.4)
Kineococcus	0.0012	0.00171724	0.05 (0-0.9)	0.2 (0-0.6)	0^{B} (0-0.6)	0 (0-0.3)	0 (0-1)	0^{B} (0-0.2)	0 (0-0.7)	0 (0-0.2)	0 (0-0.4)	0^{B} (0-0)	0^{B} (0-0)	0 (0-0.4)
Microbacteriaceae	0.0013	0.0026	0.8 (0.3-4.2)	1.2 (0.6-4.6)	0 (0-3)	0.35 (0-1.8)	1.15 (0-2.9)	$0.05^{A, B}$ (0-0.4)	0.5 (0-4.3)	1 (0-4.4)	0.6 (0.1-3.8)	0.65 (0-3.1)	0.05 (0-4.3)	0.2 (0-2.2)
Curtobacterium	0.0001	0.0001	0.2 (0-1.3)	0.2 (0-0.5)	0 (0-0.6)	0 (0-1.4)	0.2 (0-0.5)	0^{B} (0-0)	0 (0-0.4)	0.1 (0-0.6)	0 (0-0.2)	0^{B} (0-0.2)	0^{B} (0-0)	0^{B} (0-0.1)
Leucobacter	0.0453	0.19788947	0.15 (0-1.2)	0 (0-0.5)	0 (0-0.3)	0 (0-0.5)	0 (0-1.2)	0 (0-0.1)	0.1 (0-1.3)	0.1 (0-3.3)	0 (0-0.4)	0.15 (0-3.1)	0 (0-4.3)	0 (0-0.7)
Microbacterium	0.0001	0.00010122	0.1 (0-1.3)	0.3 (0-3.7)	0 (0-0.3)	0.05 (0-0.3)	0.15 (0-2.3)	0^{B} (0-0.1)	0.1 (0-1.4)	0.2 (0-0.3)	0 (0-1.4)	$0^{B, E}$ (0-0)	0^{B} (0-0.6)	0 (0-0.8)
Micrococcaceae	0.0056	0.01357576	0.4 (0-4.6)	0.8 (0.1-2.8)	0.1 (0-1.2)	0 (0-1.3)	0.45 (0-3.3)	0 (0-6.1)	0.2 (0-0.6)	0.2 (0-1.6)	0.5 (0-3.6)	0.15 (0-1.2)	0 (0-1.3)	0.2 (0-3.2)
Kocuria	0.2907	3.44687143	0 (0-0.1)	0 (0-0.2)	0 (0-0.2)	0 (0-0.1)	0.05 (0-0.9)	0 (0-0.1)	0 (0-0.2)	0 (0-0.1)	0 (0-0.3)	0.05 (0-0.4)	0 (0-0.7)	0 (0-0.2)
Micrococcus	0.0028	0.00455686	0.35 (0-4.4)	0.5 (0-2.2)	0 (0-0.7)	0 (0-1.3)	0.15 (0-1.2)	0 (0-5.6)	0 (0-0.5)	0 (0-0.9)	0.45 (0-2)	0^{B} (0-1.1)	0^{B} (0-0.5)	0 (0-2.1)
Micromonosporaceae	0.0007	0.00116667	0.1 (0-0.4)	0 (0-0.3)	0 (0-0)	0 (0-0.6)	0.2^{C} (0-1)	0 (0-0.1)	0.1 (0-0.6)	0 (0-0.3)	0.1 (0-1.7)	0^{E} (0-0.1)	0^{E} (0-0.1)	0 (0-0.3)
Mycobacteriaceae	0.0001	0.00010127	1 (0.1-4)	0.7 (0.1-1.8)	0.1 (0-2.8)	0.3 (0-4.1)	1.2 (0-3)	$0^{A, E}$ (0-0.5)	0.4 (0.1-3.2)	0.3 (0-8.4)	0.4 (0.1-6.3)	$0^{A, B, E, G}$ (0-0.5)	$0^{A, E}$ (0-2.6)	0.4 (0-0.9)
Mycobacterium	0.0001	0.00010247	1 (0.1-4)	0.7 (0.1-1.8)	0.1 (0-2.8)	0.3 (0-4.1)	1.2 (0-3)	$0^{A, E}$ (0-0.5)	0.4 (0.1-3.2)	0.3 (0-8.4)	0.4 (0.1-6.3)	$0^{A, B, E, G}$ (0-0.5)	$0^{A, E}$ (0-2.6)	0.4 (0-0.9)
Nocardiaceae	0.0001	0.00010256	0.35 (0-6.6)	0.2 (0-3.9)	0 (0-1.6)	0.05 (0-1.7)	0.65 (0-5.4)	$0^{A, E}$ (0-0)	0.4 (0-1.9)	0 (0-11.5)	0.1 (0-2.8)	$0^{A, B, E}$ (0-0)	0^{E} (0-0.4)	0 (0-3.2)
Rhodococcus	0.0001	0.00010375	0.35 (0-6.6)	0.2 (0-3.9)	0 (0-1.6)	0.05 (0-1.6)	0.6 (0-5.4)	$0^{A, E}$ (0-0)	0.4 (0-1.9)	0 (0-11.5)	0.1 (0-2.8)	$0^{A, B, E}$ (0-0)	0 (0-0.4)	0 (0-3.2)
Nocardioidaceae	0.0001	0.0001039	2.25 (0-9.1)	1.6 (0.2-2.8)	0^{A} (0-4.9)	0.7 (0-6.5)	1.2 (0-4.7)	0.05^{A} (0-0.4)	1.4 (0-3.4)	0.6 (0-2.1)	1 (0-5.8)	$0^{A, B, E, I}$ (0-1.2)	$0^{A, B, E, I}$ (0-0.3)	0.5 (0-0.9)
Aeromicrobium	0.0004	0.00048824	0.35 (0-2.4)	0.1 (0-1)	0^{A} (0-0.2)	0 (0-0.8)	0 (0-0.5)	0^{A} (0-0.2)	0 (0-0.9)	0 (0-0.4)	0.05 (0-1)	0^{A} (0-0)	0^{A} (0-0)	0 (0-0.4)
Friedmanniella	0.0219	0.05680313	0.1 (0-1.1)	0.1 (0-0.6)	0 (0-0.7)	0.05 (0-0.6)	0 (0-1.1)	0 (0-0.1)	0 (0-0.6)	0 (0-0.6)	0.05 (0-1)	0 (0-0.6)	0 (0-0.3)	0 (0-0)
Nocardioides	0.0001	0.00010506	0.1 (0-1.1)	0.1 (0-1.2)	0 (0-0.8)	0 (0-0.3)	0.1 (0-0.7)	0 (0-0.1)	0.1 (0-0.3)	0 (0-0.2)	0.05 (0-0.8)	0 (0-0)	0 (0-0)	0 (0-0.1)
Propionibacteriaceae	0.0011	0.00204651	0.85 (0.1-5.1)	3.5 (0.3-9.5)	0.3 (0-3.6)	0.3 (0-5.6)	0.65 (0-2.5)	0.05^{B} (0-0.9)	0.6 (0.1-3.7)	0.5 (0-5.5)	0.6 (0-3.1)	0.15^{B} (0-5)	0.1^{B} (0-1.1)	0.5 (0-2.7)
Microlunatus	0.0391	0.14751364	0.3 (0-0.6)	0 (0-0.5)	0 (0-0.2)	0 (0-1.1)	0 (0-1.3)	0 (0-0.1)	0 (0-0.9)	0 (0-0.3)	0 (0-0.8)	0 (0-0.1)	0^{A} (0-0)	0 (0-0.2)
Propionibacterium	0.0107	0.02277179	0.3 (0-4.8)	3.3 (0.2-9.5)	0.2 (0-3.4)	0.2 (0-5.6)	0.4 (0-2.3)	0^{B} (0-0.7)	0.4 (0-3.2)	0.3 (0-5)	0.2 (0-3.1)	0.1^{B} (0-5)	0.1^{B} (0-1.1)	0.3 (0-2.6)
Pseudonocardiaceae	0.0006	0.00092308	0.5 (0-1.1)	0.2 (0-1.6)	0.1 (0-1.2)	0.2 (0-2)	0.5 (0-2.2)	$0^{A, E}$ (0-0.1)	0.1 (0-3.3)	0 (0-1.2)	0.4 (0-2.2)	0 (0-0.4)	0^{E} (0-0.9)	0.1 (0-1.9)
Actinomycetospora	0.0216	0.05432727	0 (0-0.2)	0 (0-0.3)	0 (0-0.1)	0 (0-0.2)	0 (0-0.4)	0 (0-0)	0 (0-0.8)	0 (0-0.1)	0 (0-0.2)	0 (0-0)	0 (0-0)	0 (0-0.2)
Pseudonocardia	0.0273	0.07813448	0.1 (0-0.8)	0.2 (0-1.2)	0 (0-1.1)	0.15 (0-1.7)	0.45 (0-2.2)	0 (0-0.1)	0.1 (0-2.7)	0 (0-1.1)	0.35 (0-2.1)	0 (0-0.4)	0 (0-0.9)	0 (0-1.9)
Sporichthyaceae	0.1523	2.4368	0.05 (0-0.4)	0 (0-0.7)	0 (0-0.2)	0 (0-0.4)	0 (0-0.1)	0 (0-0)	0 (0-0.2)	0 (0-0.3)	0 (0-0.1)	0 (0-0.1)	0 (0-0.2)	0 (0-0.3)
Streptomycetaceae	0.0001	0.00010526	0.2 (0-0.9)	0 (0-0.2)	0^{A} (0-0.2)	0.05 (0-1.2)	0.05 (0-0.6)	0^{A} (0-0.1)	0 (0-0.6)	0 (0-0.2)	0.2 (0-1.4)	$0^{A, I}$ (0-0)	0^{A} (0-0.1)	0 (0-0.1)
Streptomyces	0.0013	0.00192679	0.05 (0-0.4)	0 (0-0)	0 (0-0.2)	0 (0-1.1)	0 (0-0.3)	0 (0-0)	0 (0-0.2)	0 (0-0.2)	0.15 (0-1)	0^{I} (0-0)	0 (0-0.1)	0 (0-0.1)
Williamsiaceae	0.0001	0.00010667	0.05 (0-0.7)	0.3 (0-0.5)	0^{B} (0-0.1)	0 (0-0.4)	0.1 (0-0.5)	0^{B} (0-0)	0 (0-0.3)	0 (0-1.2)	0^{B} (0-0.2)	0^{B} (0-0.2)	0^{B} (0-0)	0 (0-0.4)
Williamsia	0.0001	0.00010641	0.05 (0-0.7)	0.3 (0-0.5)	0^{B} (0-0.1)	0 (0-0.4)	0.1 (0-0.5)	0^{B} (0-0)	0 (0-0.3)	0 (0-1.2)	0^{B} (0-0.2)	0^{B} (0-0.2)	0^{B} (0-0)	0 (0-0.4)
Rubrobacteria	0.0513	0.26676	0.1 (0-0.4)	0 (0-0.4)	0 (0-0.6)	0 (0-0.4)	0.05 (0-2.5)	0 (0-0.1)	0 (0-0.6)	0 (0-0.6)	0 (0-1)	0 (0-0)	0 (0-0.8)	0 (0-0)
Rubrobacterales	0.0513	0.577125	0.1 (0-0.4)	0 (0-0.4)	0 (0-0.6)	0 (0-0.4)	0.05 (0-2.5)	0 (0-0.1)	0 (0-0.6)	0 (0-0.6)	0 (0-1)	0 (0-0)	0 (0-0.8)	0 (0-0)
Rubrobacteraceae	0.0513	0.4104	0.1 (0-0.4)	0 (0-0.4)	0 (0-0.6)	0 (0-0.4)	0.05 (0-2.5)	0 (0-0.1)	0 (0-0.6)	0 (0-0.6)	0 (0-1)	0 (0-0)	0 (0-0.8)	0 (0-0)
Rubrobacter	0.0513	0.23655	0.1 (0-0.4)	0 (0-0.4)	0 (0-0.6)	0 (0-0.4)	0.05 (0-2.5)	0 (0-0.1)	0 (0-0.6)	0 (0-0.6)	0 (0-1)	0 (0-0)	0 (0-0.8)	0 (0-0)
Thermoleophilia	0.0004	0.00049524	2.8 (0-16.8)	2.2 (0.1-5.9)	0 (0-2.7)	0.7 (0-17.1)	2.95 (0-14.4)	0.1 (0-1.1)	0.6 (0-15.7)	0.3 (0-5.1)	0.75 (0-26.6)	0 (0-1.2)	0 (0-3.2)	0.3 (0-5.1)
Gaiellales	0.0028	0.007	1.7 (0-7.8)	0.9 (0-2.9)	0 (0-2)	0.5 (0-8.2)	1.25 (0-7.5)	0 (0-0.3)	0.4 (0-7.2)	0.2 (0-2.2)	0.2 (0-10.4)	0 (0-0.8)	0 (0-1.1)	0.1 (0-2.7)
Gaiellaceae	0.0042	0.0096	1.65 (0-7.6)	0.9 (0-2.8)	0 (0-1.9)	0.5 (0-8.1)	1.25 (0-6.9)	0 (0-0.3)	0.4 (0-7.2)	0.1 (0-2.2)	0.2 (0-10.1)	0 (0-0.8)	0 (0-1.1)	0.1 (0-2.7)
Solirubrobacterales	0.0004	0.0006	1.2 (0-9)	1.3 (0-3)	0 (0-0.8)	0.45 (0-8.9)	1.25 (0-6.9)	0.05 (0-0.9)	0.5 (0-8.5)	0.2 (0-2.9)	0.6 (0-16.2)	0 (0-0.4)	0 (0-2.1)	0.3 (0-2.4)
Patulibacteraceae	0.0132	0.048	0.05 (0-0.6)	0 (0-0.5)	0 (0-0.4)	0 (0-0.2)	0 (0-0.9)	0 (0-0)	0 (0-0.3)	0.1 (0-0.6)	0 (0-0.8)	0 (0-0)	0 (0-0.6)	0 (0-0)
Solirubrobacteraceae	0.0003	0.00043636	0.35 (0-1.4)	0.1 (0-1.2)	0 (0-0.7)	0.1 (0-1.9)	0.5 (0-2.3)	0 (0-0.3)	0.2 (0-2)	0 (0-0.5)	0.35 (0-3.2)	0 (0-0.2)	0^{I} (0-0.9)	0.1 (0-0.4)
Bacteroidetes	0.0001	0.0001	37.5 (0.5 - 62.8)	4.1 (0.6 - 18.2)	0.2 (0 - 3.1)	1.4 (0 - 4.1)	5.55^{C} (1.1 - 27.8)	13.4C (0.1 - 78.4)	1 (0 - 5.6)	4.2 (0 - 22.9)	1.6 (0 - 16.1)	13.95C (0 - 61)	0.1^{J} (0 - 44.6)	1.6 (0 - 24.2)
Bacteroidia	0.0001	0.0001	2.55 (0-62.4)	2.5 (0.1-16.3)	0.1 (0-1.9)	0.05 (0-3.9)	1.7 (0-26.7)	12.75 (0.1-78.4)	0.2 (0-4.8)	3.7 (0-22.9)	0.1 (0-15.3)	$13.45^{C, D, G}$ (0-59.4)	0.05 (0-44.3)	0 (0-23.4)
Bacteroidales	0.0001	0.0001	2.55 (0-62.4)	2.5 (0.1-16.3)	0.1 (0-1.9)	0.05 (0-3.9)	1.7 (0-26.7)	12.75 (0.1-78.4)	0.2 (0-4.8)	3.7 (0-22.9)	0.1 (0-15.3)	$13.45^{C, D, G}$ (0-59.4)	0.05 (0-44.3)	0 (0-23.4)
Paraprevotellaceae	0.0012	0.00234146	0.2 (0-57.7)	0.4 (0-1.7)	0 (0-0.2)	0 (0-0.3)	0.15 (0-5.3)	0.8 (0-10.4)	0 (0-1.1)	0.3 (0-3.8)	0 (0-1.1)	$1.4^{C, D, I}$ (0-20.1)	0 (0-2)	0 (0-1)
Prevotella	0.0012	0.00174737	0.2 (0-0.8)	0.4 (0-1.7)	0 (0-0.2)	0 (0-0.3)	0.15 (0-5.3)	0.8 (0-10.4)	0 (0-1.1)	0.3 (0-3.8)	0 (0-1.1)	$1.1^{C, D, I}$ (0-20.1)	0 (0-2)	0 (0-1)
Bacteroidaceae	0.0001	0.00010811	0 (0-0.2)	0 (0-0)	0 (0-0.1)	0 (0-0.3)	0 (0-4.5)	$1.6^{A, B, C, D, E}$ (0-18.5)	0^{F} (0-0.1)	0 (0-17.9)	0^{F} (0-0)	0^{F} (0-0.9)	0^{F} (0-25.2)	0^{F} (0-0)
Bacteroides	0.0001	0.00010779	0 (0-0.2)	0 (0-0)	0 (0-0.1)	0 (0-0.3)	0 (0-4.5)	$1.6^{A, B, C, D, E}$ (0-18.5)	0^{F} (0-0.1)	0 (0-17.9)	0^{F} (0-0)	0^{F} (0-0.9)	0^{F} (0-25.2)	0^{F} (0-0)
Porphyromonadaceae	0.0006	0.00094118	1.6 (0-12.6)	1.5 (0-14.4)	0 (0-1.9)	0 (0-3.5)	1 (0-22.7)	0.65 (0-71.7)	0.1 (0-3.3)	1.1 (0-16.5)	0.1 (0-15.2)	$8^{C, D}$ (0-54.4)	0.05 (0-41)	0 (0-22.9)
Porphyromonas	0.0006	0.00076615	1.45 (0-12.4)	1.5 (0-13.6)	0 (0-1.6)	0 (0-3.2)	1 (0-22.3)	0.25 (0-70.5)	0.1 (0-3.3)	1.1 (0-15.6)	0.1 (0-14)	$7.95^{C, D}$ (0-51.5)	0.05 (0-40.2)	0 (0-22.5)
Flavobacteriia	0.0019	0.00274444	0.2 (0-2.1)	0.3 (0-1.6)	0 (0-0.2)	0.05 (0-0.7)	0.4^{C} (0-1.3)	0 (0-0.6)	0.1 (0-0.7)	0.2 (0-1.6)	0.05 (0-0.8)	0.5^{C} (0-9.6)	0 (0-2.6)	0.1 (0-1.6)
Flavobacteriales	0.0019	0.004275	0.2 (0-2.1)	0.3 (0-1.6)	0 (0-0.2)	0.05 (0-0.7)	0.4^{C} (0-1.3)	0 (0-0.6)	0.1 (0-0.7)	0.2 (0-1.6)	0.05 (0-0.8)	0.5^{C} (0-0.6)	0 (0-2.6)	0.1 (0-1.6)
Flavobacteriaceae	0.0019	0.00410811	0.2 (0-2.1)	0.3 (0-1.6)	0 (0-0.2)	0.05 (0-0.7)	0.4^{C} (0-01.3)	0 (0-0.6)	0.1 (0-0.7)	0.2 (0-1.6)	0.05 (0-0.8)	0.5^{C} (0-9.6)	0 (0-2.6)	0.1 (0-1.6)
Bergeyella	0.0313	0.09621852	0 (0-0.7)	0.1 (0-1.1)	0 (0-0)	0 (0-0.2)	0.1 (0-0.6)	0 (0-0.6)	0 (0-0.2)	0 (0-1.4)	0 (0-0.1)	0.1 (0-8.3)	0 (0-0.2)	0 (0-0.8)

Capnocytophaga	0.0735	0.38128125	0 (0-0.9)	0 (0-0.6)	0 (0-0.1)	0 (0-0.1)	0.1 (0-0.7)	0 (0-0.2)	0 (0-0.3)	0.1 (0-1)	0 (0-0.4)	0.05 (0-0.8)	0 (0-2.1)	0 (0-0.6)
Flavobacterium	0.0263	0.07276333	0 (0-0.8)	0.1 (0-0.3)	0 (0-0)	0 (0-0.6)	0 (0-0.4)	0 (0-0)	0 (0-0.3)	0 (0-0.1)	0 (0-0.3)	0 (0-0.2)	0 (0-0)	0 (0-0.6)
Sphingobacteriia	0.0001	0.000104	0.4 (0.2-1.8)	0.3 (0-1.7)	0 (0-1.9)	0.25 (0-2.6)	0.5 (0-8.3)	0 (0-0.7)	0.7 (0-1.3)	0.1 (0-0.9)	0.75 (0-1.5)	0^{A} (0-0.9)	$0^{A, E, G}$ (0-0.1)	0 (0-1)
Sphingobacteriales	0.0001	0.00010227	0.4 (0.2-1.8)	0.3 (0-1.7)	0 (0-1.9)	0.25 (0-2.6)	0.5 (0-8.3)	0 (0-0.7)	0.7 (0-1.3)	0.1 (0-0.9)	0.75 (0-1.5)	0^{A} (0-0.9)	$0^{A, E, G}$ (0-0.1)	0 (0-1)
Chitinophagaceae	0.0009	0.0016	0.25 (0-1.4)	0.1 (0-1)	0 (0-1.7)	0.05 (0-2.1)	0.25 (0-1.3)	0 (0-0.3)	0.3 (0-1)	0 (0-0.5)	0.35 (0-1.3)	0 (0-0.1)	0^{A} (0-0.1)	0 (0-0.7)
Flavisolibacter	0.0354	0.117528	0.1 (0-0.9)	0 (0-0.3)	0 (0-1.1)	0 (0-1.4)	0.05 (0-0.9)	0 (0-0.3)	0 (0-0.7)	0 (0-0.4)	0.05 (0-1.2)	0 (0-0.1)	0 (0-0)	0 (0-0.2)
Flexibacteraceae	0.0138	0.0552	0.1 (0-0.8)	0.1 (0-1.7)	0 (0-0.1)	0 (0-0.4)	0.05 (0-7.3)	0 (0-0.4)	0 (0-0.5)	0 (0-0.4)	0 (0-0.3)	0 (0-0.8)	0^{B} (0-0)	0 (0-0.4)
Chloroflexi	0.0143	0.0286	0.4 (0 - 1.7)	0.2 (0 - 0.8)	0 (0 - 1.3)	0.05 (0 - 2.7)	0.35 (0 - 2.3)	0 (0 - 0.6)	0.2 (0 - 3.8)	0 (0 - 2.3)	0.85 (0 - 2.8)	0.05 (0 - 0.5)	0 (0 - 0.5)	0.5 (0 - 1.2)
Chloroflexi	0.2783	7.2358	0 (0-0.5)	0 (0-0.8)	0 (0-0.5)	0 (0-1.4)	0.05 (0-1.4)	0 (0-0.3)	0 (0-3.8)	0 (0-0.9)	0.15 (0-0.9)	0 (0-0.2)	0 (0-0.4)	0 (0-0.6)
Cyanobacteria	0.0001	0.00010769	2.35 (0.2 - 42.1)	3 (0.2 - 5.5)	0.6 (0 - 24.2)	1.5 (0 - 7.2)	2.7 (0.4 - 36.1)	$0^{A, B, E}$ (0 - 0.7)	2.5^{F} (0 - 21.1)	1.5 (0 - 11.7)	3.95 (0 - 9.4)	$0.05^{A, B, E, G}$ (0 - 0.4)	$0^{A, B, E, G}$ (0 - 1.6)	0.4 (0 - 4.9)
Chloroplast	0.0001	0.00010833	1.65 (0.1-8.9)	1.9 (0-4)	0.4 (0-24)	0.95 (0-3.7)	1.5 (0.1-36.1)	$0^{A, E}$ (0-0.3)	1.3 (0-20.5)	1.1 (0-8.5)	2.2 (0-7.1)	$0^{A, B, E}$ (0-0.3)	$0^{A, E}$ (0-0.7)	0.2 (0-2.4)
Chlorophyta	0.0087	0.02796429	0.05 (0-1.1)	0 (0-0.5)	0 (0-0)	0.05 (0-1)	0 (0-1.5)	0 (0-0.1)	0 (0-2.8)	0 (0-0.3)	0 (0-0.9)	0 (0-0)	0 (0-0)	0 (0-0.4)
Stramenopiles	0.0013	0.00265909	0.35 (0-7.1)	0 (0-2.2)	0 (0-1.2)	0 (0-2.4)	0.05 (0-3)	0 (0-0.1)	0 (0-2.3)	0.1 (0-7.4)	0.15 (0-5.9)	0^{A} (0-0.1)	0^{A} (0-0)	0 (0-2.2)
Streptophyta	0.0001	0.00010465	0.7 (0-7.1)	0.8 (0-3.8)	0.3 (0-24)	0.1 (0-2.7)	0.85 (0-36.1)	$0^{A, B, E}$ (0-0.3)	0.4 (0-20.3)	0.1 (0-2.5)	0.1 (0-3.5)	$0^{A, B, E}$ (0-0.3)	0^{E} (0-0.7)	0.1 (0-1.3)
Nostocophycideae	0.0477	0.2067	0 (0-0.6)	0.1 (0-0.6)	0 (0-1)	0 (0-3.5)	0 (0-0.6)	0 (0-0.4)	0.1 (0-1.2)	0 (0-3.8)	0 (0-0.8)	0 (0-0.1)	0 (0-0)	0 (0-1)
Nostocales	0.2031	9.1395	0 (0-0.6)	0 (0-0.6)	0 (0-1)	0 (0-3.5)	0 (0-0.6)	0 (0-0)	0 (0-1.2)	0 (0-3.8)	0 (0-0.8)	0 (0-0.1)	0 (0-0)	0 (0-1)
Nostocaceae	0.4639	18.556	0 (0-0.6)	0 (0-0.6)	0 (0-1)	0 (0-3.5)	0 (0-0.6)	0 (0-0)	0 (0-1.2)	0 (0-3.3)	0 (0-0.8)	0 (0-0.1)	0 (0-0)	0 (0-0.7)
Oscillatoriophycideae	0.0001	0.00011304	0.3 (0-34.9)	0.3 (0-2.6)	0 (0-1.2)	0 (0-2.1)	0.7 (0-3.4)	0 (0-0.5)	0.4 (0-1.8)	0.1 (0-2.4)	0.65 (0-2.5)	$0^{A, E, I}$ (0-0.1)	$0^{A, E, I}$ (0-0.4)	0 (0-1.1)
Chroococcales	0.0007	0.00116667	0.15 (0-34.9)	0.1 (0-0.4)	0 (0-0.2)	0 (0-0.5)	0.1 (0-2.8)	0 (0-0.1)	0 (0-1.4)	0 (0-0.6)	0.2 (0-2.5)	0 (0-0.1)	0 (0-0.4)	0 (0-0.4)
Xenococcaceae	0.0519	0.46133333	0.05 (0-34.9)	0 (0-0.4)	0 (0-0)	0 (0-0.5)	0 (0-2.8)	0 (0-0.1)	0 (0-1.3)	0 (0-0.6)	0 (0-2.4)	0 (0-0.1)	0 (0-0.1)	0 (0-0.3)
Oscillatoriales	0.037	0.208125	0 (0-1.6)	0 (0-2.6)	0 (0-0.9)	0 (0-1.3)	0 (0-1.7)	0 (0-0.4)	0 (0-0.6)	0 (0-2)	0.1 (0-0.8)	0 (0-0)	0 (0-0)	0 (0-0.5)
Phormidiaceae	0.037	0.21142857	0 (0-1.6)	0 (0-2.6)	0 (0-0.9)	0 (0-1.3)	0 (0-1.7)	0 (0-0.4)	0 (0-0.6)	0 (0-2)	0.1 (0-0.8)	0 (0-0)	0 (0-0)	0 (0-0.5)
Phormidium	0.037	0.13352174	0 (0-1.6)	0 (0-2.6)	0 (0-0.9)	0 (0-1.3)	0 (0-1.7)	0 (0-0.4)	0 (0-0.6)	0 (0-2)	0.1 (0-0.8)	0 (0-0)	0 (0-0)	0 (0-0.5)
Synechococcophycideae	0.1071	0.9282	0 (0-0.5)	0.1 (0-0.5)	0 (0-0.8)	0 (0-0.7)	0.05 (0-1.4)	0 (0-0)	0 (0-5.1)	0 (0-0.7)	0 (0-0.4)	0 (0-0.2)	0 (0-0.5)	0 (0-0.2)
Pseudanabaenales	0.0227	0.10215	0 (0-0.5)	0 (0-0.5)	0 (0-0)	0 (0-0.2)	0.05 (0-1.4)	0 (0-0)	0 (0-0.7)	0 (0-0.6)	0 (0-0.4)	0 (0-0.2)	0 (0-0.4)	0 (0-0)
Pseudanabaenaceae	0.0227	0.10682353	0 (0-0.5)	0 (0-0.5)	0 (0-0)	0 (0-0.2)	0.05 (0-1.4)	0 (0-0)	0 (0-0.7)	0 (0-0.6)	0 (0-0.4)	0 (0-0.2)	0 (0-0.4)	0 (0-0)
Firmicutes	0.0675	0.315	16.3 (5 - 48.7)	15.6 (3.5 - 51.3)	9.6 (0.6 - 44.5)	13.8 (0.2 - 69.9)	13 (3.2 - 44.2)	28.1 (4.8 - 62.8)	16.7 (0 - 51.2)	15.1 (1.1 - 31.8)	9.55 (1 - 55.3)	12.65 (0.3 - 28.9)	5.55 (0.8 - 51)	19.1 (0.6 - 67)
Bacilli	0.0946	0.6149	14.4 (4.3-45.7)	13.9 (3.1-49.9)	8.9 (0.6-43.8)	9.8 (0.1-69.2)	8.15 (2.9-37.4)	3.25 (0.1-30.2)	16.7 (0-49.5)	13.2 (0.3-28)	8.3 (1-51.9)	5.95 (0-25.8)	2.95 (0.1-50.4)	19.1 (0.3-64.5)
Bacillales	0.0012	0.00225	10.05 (0.9-40.9)	6.4 (2.8-47.6)	8.7 (0.5-40.4)	7.35 (0.1-68.7)	5.4 (1.7-31.9)	1.15 (0-29.8)	11.4 (0-46.8)	5.6 (0-24.8)	3.55 (0.9-44.5)	$0.75^{A, G}$ (0-15.4)	1.85 (0.1-50.2)	19.1^{I} (.1-59.3)
Alicyclobacillaceae	0.0063	0.0168	0.45 (0-2.2)	0.7 (0.2-7.7)	4.3 (0-10.9)	0.5 (0-6.7)	0.7 (0.1-6)	0.05 (0-1.7)	1.5 (0-21.8)	0.2 (0-2.9)	0.35 (0-4)	0.15 (0-2.4)	0.45 (0-11.5)	1 (0-19.1)
Alicyclobacillus	0.0061	0.01150682	0.45 (0-2.2)	0.7 (0.2-7.7)	4.3 (0-10.9)	0.5 (0-6.7)	0.7 (0.1-6)	0.05^{C} (0-1.7)	1.5 (0-21.8)	0.2 (0-2.9)	0.35 (0-4)	0.15 (0-2.4)	0.45 (0-11.5)	1 (0-19.1)
Bacillaceae	0.0001	0.00010959	3.55 (0-10.6)	2.2 (0.8-8.5)	0^{A} (0-7.6)	0.75 (0-7.6)	2 (0.4-6.4)	$0^{A, B, E}$ (0-1)	1.3 (0-14.4)	1 (0-18.3)	1.5 (0.1-9.2)	$0^{A, B, E}$ (0-1.5)	$0.1^{A, B, E}$ (0-1.3)	0.5 (0-3.1)
Bacillus	0.0001	0.00010921	3.4 (0-10.5)	1.7 (0.7-8.5)	0^{A} (0-7.5)	0.7 (0-7.5)	1.6 (0.3-5.9)	$0^{A, B, E}$ (0-1)	1.3 (0-14.1)	1 (0-17)	1.35 (0.1-9.2)	$0^{A, B, E}$ (0-1.3)	$0^{A, B, E}$ (0-1.3)	0.5 (0-2.7)
Paenibacillaceae	0.0006	0.00096	0.3 (0-1.6)	0 (0-0.9)	0 (0-0.6)	0 (0-0.4)	0.2 (0-0.7)	0^{A} (0-0)	0.1 (0-1.1)	0.1 (0-2.2)	0.1 (0-1.4)	0 (0-0.3)	0^{A} (0-0.1)	0 (0-0.5)
Ammoniphilus	0.003	0.00498	0 (0-0.7)	0 (0-0)	0 (0-0.6)	0 (0-0)	0.05 (0-0.3)	0 (0-0)	0 (0-0.2)	0 (0-0.3)	0 (0-0.7)	0 (0-0.3)	0 (0-0)	0 (0-0.3)
Paenibacillus	0.01	0.0202439	0.15 (0-0.8)	0 (0-0.5)	0 (0-0.2)	0 (0-0.3)	0 (0-0.3)	0^{A} (0-0)	0 (0-0.9)	0 (0-0.3)	0 (0-0.4)	0 (0-0.1)	0 (0-0.1)	0 (0-0.1)
Planococcaceae	0.0001	0.00011111	0.95 (0-2.3)	0.8 (0-2.3)	0 (0-0.7)	0.15 (0-2.7)	0.4 (0-3.1)	0 (0-0.4)	0.7 (0-1.5)	0.2 (0-1.1)	0.15 (0-5.5)	$0^{A, E}$ (0-0.2)	$0^{A, E, G}$ (0-0)	0 (0-4)
Rummeliibacillus	0.1893	1.74576667	0 (0-0.2)	0 (0-0.2)	0 (0-0.2)	0 (0-2.4)	0 (0-0.7)	0 (0-0)	0 (0-0.4)	0 (0-0.4)	0 (0-0)	0 (0-0)	0 (0-0)	0 (0-0)
Sporosarcina	0.0032	0.00542041	0.1 (0-1.6)	0.5 (0-0.9)	0 (0-0.3)	0 (0-1)	0.2 (0-1.2)	0 (0-0.3)	0.2 (0-1)	0 (0-0.8)	0 (0-1.9)	0 (0-0)	0 (0-0)	0 (0-0.4)
Staphylococcaceae	0.1726	3.452	2.1 (0.2-33.9)	2.1 (0.2-39.5)	1 (0-30.2)	2.8 (0-66.5)	1.35 (0-24.2)	0.35 (0-29.6)	0.8 (0-42.5)	0.6 (0-22)	1.15 (0-30.4)	0.25 (0-12)	0.35 (0-42.9)	2.3 (0-53.3)
Macrococcus	0.4394	12.1567333	0.1 (0-32.2)	0 (0-38.7)	0 (0-27.7)	0.05 (0-60)	0 (0-20.1)	0 (0-26.2)	0 (0-40.6)	0 (0-10.2)	0 (0-28.5)	0 (0-0.2)	0 (0-0.1)	0 (0-12.5)
Salinicoccus	0.358	7.4285	0 (0-1.9)	0 (0-0)	0 (0-0.4)	0 (0-0.1)	0 (0-0.3)	0 (0-0)	0 (0-0.1)	0 (0-0.1)	0 (0-0.2)	0 (0-0)	0 (0-0)	0 (0-0.1)
Staphylococcus	0.3408	5.65728	0.85 (0.2-8.6)	1.6 (0.2-4)	1 (0-8.9)	1.25 (0-66.2)	1 (0-4)	0.2 (0-3.2)	0.7 (0-1.9)	0.5 (0-14.1)	0.95 (0-3.4)	0.25 (0-12)	0.35 (0-42.9)	2.2 (0-53.3)
Exiguobacterales	0.0062	0.0186	0.1 (0-1.5)	0.1 (0-0.7)	0 (0-0.1)	0 (0-0.5)	0 (0-0.5)	0 (0-0.1)	0 (0-0.6)	0.1 (0-0.4)	0 (0-0.2)	0 (0-0)	0 (0-0.1)	0 (0-1.8)
Exiguobacteraceae	0.0062	0.016	0.1 (0-1.5)	0.1 (0-0.7)	0 (0-0.1)	0 (0-0.5)	0 (0-0.5)	0 (0-0.1)	0 (0-0.6)	0.1 (0-0.4)	0 (0-0.2)	0 (0-0)	0 (0-0.1)	0 (0-1.8)
Exiguobacterium	0.0062	0.01196744	0.1 (0-1.5)	0.1 (0-0.7)	0 (0-0.1)	0 (0-0.5)	0 (0-0.5)	0 (0-0.1)	0 (0-0.6)	0.1 (0-0.4)	0 (0-0.2)	0 (0-0)	0 (0-0.1)	0 (0-1.8)
Lactobacillales	0.0046	0.0129375	0.9 (0-33.6)	2.1 (0.3-8.2)	0.1 (0-3.1)	0.25 (0-13)	2.6 (0-17.4)	1.15 (0-12.3)	0.5 (0-10.4)	2.9 (0.3-18.5)	0.25 (0-7.6)	3.35 (0-24)	0^{I} (0-3.3)	1.9 (0-8.5)
Aerococcaceae	0.001	0.00181818	0.05 (0-1.4)	0.1 (0-2.2)	0 (0-0.4)	0 (0-0.9)	0.15 (0-2)	0 (0-8.1)	0 (0-0.4)	0.3 (0-3.2)	0.05 (0-1.1)	0.6 (0-10.3)	0^{I} (0-1.1)	0.2 (0-2.3)
Abiotrophia	0.0016	0.00250566	0 (0-1.3)	0.1 (0-0.8)	0 (0-0.4)	0 (0-0.8)	0.1 (0-1.6)	0 (0-8.1)	0 (0-0.3)	0.3 (0-3.2)	0 (0-0.7)	$0.6^{C, D, G}$ (0-10.2)	0^{I} (0-1.1)	0 (0-2.2)
Enterococcaceae	0.3264	8.704	0 (0-2.2)	0 (0-0.7)	0 (0-0.1)	0 (0-0.4)	0 (0-1.9)	0.05 (0-7.6)	0 (0-0.8)	0 (0-1.8)	0 (0-4)	0 (0-0.1)	0 (0-0.3)	0 (0-1)
Streptococcaceae	0.006	0.015	0.35 (0-33.5)	0.4 (0.1-7.3)	0.1 (0-1.4)	0.1 (0-1.7)	1.25 (0-17)	0.1 (0-4.2)	0.2 (0-9.4)	1.1 (0-17.9)	0.05 (0-7.6)	2.95 (0-21)	0 (0-3)	0 (0-2.8)
Streptococcus	0.0015	0.00230556	0.3 (0-33.3)	0.3 (0-7.1)	0 (0-0.8)	0.1 (0-1.7)	1.2 (0-12.3)	0.1 (0-4.2)	0.1 (0-8.9)	1.1 (0-17.9)	0.05 (0-7.6)	2.9^{C} (0-21)	0 (0-2.1)	0^{I} (0-2.8)
Clostridia	0.0079	0.01711667	1.45 (0-4.1)	1.7 (0.1-3.8)	0.7 (0-1.4)	0.6 (0-7.3)	1.05 (0.3-33.9)	14.05^{C} (0.4-60.8)	0.8 (0-4.7)	1.7 (0.1-31.2)	0.65 (0-3)	4.15 (0.2-13)	0.95 (0-7.4)	0.6 (0-2.4)
Clostridiales	0.0439	0.32925	0.85 (0-4)	1.4 (0.1-2.2)	0.4 (0-1.4)	0.55 (0-5.7)	1 (0.2-31.9)	11.95 (0.3-57.3)	0.5 (0-2.9)	1.5 (0.1-28.5)	0.6 (0-3)	1.35 (0.2-5.9)	0.65 (0-6.7)	0.5 (0-2.2)
Clostridiaceae	0.0926	0.926	0.35 (0-1.3)	0.3 (0-1.5)	0.1 (0-0.8)	0.2 (0-1.3)	0.45 (0-9.4)	2 (0-15.7)	0.3 (0-2.5)	0.5 (0-4.5)	0.45 (0-1.2)	0.3 (0-2.1)	0 (0-2.1)	0.1 (0-1.3)
Clostridium	0.0054	0.00974348	0.15 (0-0.6)	0 (0-0.8)	0 (0-0.5)	0.05 (0-1.2)	0.1 (0-9.3)	0.95 (0-15.7)	0 (0-0.6)	0.1 (0-4.5)	0.15 (0-1.1)	0^{F} (0-0.2)	0^{F} (0-0.7)	0 (0-0.6)
Lachnospiraceae	0.0095	0.02923077	0 (0-1.6)	0.1 (0-1.4)	0 (0-0.6)	0 (0-1.9)	0 (0-14.4)	$5.4^{C, E}$ (0-55.6)	0^{F} (0-0.2)	0 (0-11.9)	0^{F} (0-0.3)	0.1 (0-1.3)	0^{F} (0-3.7)	0^{F} (0-0.2)
Ruminococcus	0.0001	0.00011067	0 (0-0)	0 (0-0)	0 (0-0)	0 (0-0.2)	0 (0-0.4)	$0.2^{A, B, C, D, E}$ (0-3.9)	0^{F} (0-0)	0^{F} (0-1.2)	0^{F} (0-0)	0^{F} (0-0)	0^{F} (0-0.2)	0^{F} (0-0)
Blautia	0.0006	0.00077813	0 (0-1.1)	0 (0-0.3)	0 (0-0.1)	0 (0-1.3)	0 (0-7.1)	$1.05^{A, C, E}$ (0-15.1)	0^{F} (0-0.2)	0^{F} (0-7.3)	0^{F} (0-0.1)	0^{F} (0-0.2)	0^{F} (0-0.3)	0^{F} (0-0)
Peptostreptococcaceae	0.0502	0.36509091	0.15 (0-1.2)	0.1 (0-0.5)	0 (0-0.7)	0 (0-2.9)	0.1 (0-2.9)	0.1 (0-1.3)	0.1 (0-0.8)	0.2 (0-0.6)	0.1 (0-1.3)	0.15 (0-1.3)	0 (0-0.2)	0.1 (0-1.3)
Ruminococcaceae	0.1087	1.44933333	0.1 (0-0.5)	0 (0-0.1)	0 (0-0.2)	0 (0-1)	0.1 (0-5.5)	0.45 (0-11.8)	0 (0-0.7)	0.1 (0-1.3)	0 (0-0.3)	0.05 (0-1.9)	0 (0-1.5)	0 (0-0.3)
Faecalibacterium	0.0173	0.04102571	0 (0-0)	0 (0-0.1)	0 (0-0.1)	0 (0-0.4)	0 (0-3.5)	$0.2^{A, C}$ (0-10.7)	0^{F} (0-0.1)	0 (0-0.8)	0 (0-0.2)	0^{F} (0-0.1)	0^{F} (0-0.7)	0 (0-0.1)
Veillonellaceae	0.0135	0.05142857	0 (0-0.4)	0 (0-0.7)	0 (0-0.1)	0 (0-0.9)	0 (0-2.1)	0.15 (0-5.5)	0 (0-0.6)	0 (0-10.7)	0 (0-0.1)	0^{F} (0-0.1)	0 (0-1)	0^{F} (0-0)
Megamonas	0.0006	0.00079048	0 (0-0.1)	0 (0-0.1)	0 (0-0)	0 (0-0)	0 (0-2.1)	$0.1^{A, B, C, D, E}$ (0-5.5))	0^{F} (0-0.1)	0^{F} (0-10.7)	0^{F} (0-0.1)	0^{F} (0-0)	0^{F} (0-1)	0^{F} (0-0)
Coriobacteriales	0.0001	0.00010714	0 (0-0.1)	0 (0-0.4)	0 (0-0.1)	0 (0-0.2)	0 (0-0.4)	$0.4^{A, B, C, D, E}$ (0-2.3)	0^{F} (0-0)	0^{F} (0-0.3)	0 (0-0.2)	0^{F} (0-0.1)	0^{F} (0-0.2)	0^{F} (0-0.1)
Coriobacteriaceae	0.0001	0.00011268	0 (0-0.1)	0 (0-0.4)	0 (0-0.1)	0 (0-0.2)	0 (0-0.4)	$0.4^{A, B, C, D, E}$ (0-2.3)	0^{F} (0-0)	0^{F} (0-0.3)	0 (0-0.2)	0^{F} (0-0.1)	0^{F} (0-0.2)	0^{F} (0-0.1)
Collinsella	0.0001	0.00011216	0 (0-0.1)	0 (0-0.4)	0 (0-0)	0 (0-0.2)	0 (0-0.4)	$0.4^{A, B, C, D, E}$ (0-2.2)	0^{F} (0-0)	0^{F} (0-0.3)	0^{F} (0-0.1)	0^{F} (0-0.1)	0^{F} (0-0.2)	0^{F} (0-0.1)
Erysipelotrichi	0.0012	0.00164211	0.1 (0-0.6)	0.1 (0-0.2)	0 (0-0.4)	0 (0-0.1)	0.1 (0-0.7)	0.35^{D} (0-2.1)	0^{F} (0-0.3)	0 (0-0.6)	0 (0-0.4)	0.2 (0-0.8)	0^{F} (0-0.6)	0 (0-0.5)
Erysipelotrichales	0.0012	0.00234783	0.1 (0-0.6)	0.1 (0-0.2)	0 (0-0.4)	0 (0-0.1)	0.1 (0-0.7)	0.35^{D} (0-2.1)	0^{F} (0-0.3)	0 (0-0.6)	0 (0-0.4)	0.2 (0-0.8)	0^{F} (0-0.6)	0 (0-0.5)
Coprobacillaceae	0.0001	0.00011429	0 (0-0)	0 (0-0)	0 (0-0)	0 (0-0)	0 (0-0.2)	$0.1^{A, B, C, D, E}$ (0-0.8)	0^{F} (0-0)	0^{F} (0-0.2)	0^{F} (0-0)	0^{F} (0-0)	0^{F} (0-0)	0^{F} (0-0.1)
Erysipelotrichaceae	0.0066	0.0182069	0.1 (0-0.6)	0.1 (0-0.2)	0 (0-0.4)	0 (0-0.1)	0.1 (0-0.5)	0.35 (0-1.5)	0 (0-0.3)	0 (0-0.6)	0 (0-0.4)	0.2 (0-0.8)	0 (0-0.6)	0 (0-0.5)

Eubacterium	0.0005	0.0006194	0 (0-0.4)	0 (0-0)	0 (0-0)	0 (0-0)	0 (0-0.2)	$0.05^{A, B, C, D, E}$ (0-0.8)	0^{F} (0-0)	0 (0-0.6)	0^{F} (0-0)	0^{F} (0-0.5)	0^{F} (0-0)	0^{F} (0-0)
Fusobacteria	0.0094	0.01462222	0.05 (0 - 1.5)	0.4 (0 - 2.2)	0 (0 - 0.6)	0 (0 - 0.8)	0.35 (0 - 7.5)	3.05^{D} (0 - 9.4)	0 (0 - 0.3)	0.3 (0 - 38.3)	0.1 (0 - 0.4)	0.25 (0 - 3)	0.05 (0 - 13)	0 (0 - 1.4)
Fusobacteria	0.0094	0.02221818	0.05 (0-1.5)	0.4 (0-2.2)	0 (0-0.6)	0 (0-0.8)	0.35 (0-7.5)	30.5^{D} (0-9.4)	0 (0-0.3)	0.3 (0-38.3)	0.1 (0-0.4)	0.25 (0-3)	0.05 (0-13)	0 (0-1.4)
Fusobacteriales	0.0094	0.03253846	0.05 (0-1.5)	0.4 (0-2.2)	0 (0-0.6)	0 (0-0.8)	0.35 (0-7.5)	3.05 (0-9.4)	0 (0-0.3)	0.3 (0-38.3)	0.1 (0-0.4)	0.25 (0-3)	0.05 (0-13)	0 (0-1.4)
Fusobacteriaceae	0.0101	0.03232	0.05 (0-1.5)	0.4 (0-2.2)	0 (0-0.6)	0 (0-0.8)	0.35 (0-7.4)	30.5^{D} (0-9.4)	0 (0-0.3)	0.3 (0-38.3)	0.05 (0-0.4)	0.25 (0-3)	0 (0-11.8)	0 (0-1.4)
Fusobacterium	0.2887	2.9952625	0 (0-1.1)	0.4 (0-0.8)	0 (0-0.6)	0 (0-0.5)	0.15 (0-7.4)	0 (0-2.4)	0 (0-0.3)	0.1 (0-5.2)	0 (0-0.4)	0.25 (0-3)	0 (0-11.8)	0 (0-1.4)
Gemmatimonadetes	0.0855	0.5985	0 (0 - 0.8)	0 (0 - 0.5)	0 (0 - 1.5)	0 (0 - 1.4)	0 (0 - 0.3)	0 (0 - 0.1)	0.1 (0 - 1.2)	0 (0 - 0.2)	0 (0 - 1.1)	0 (0 - 0.1)	0 (0 - 0)	0 (0 - 0.3)
Planctomycetes	0.0038	0.00483636	0.25 (0 - 1.7)	0.4 (0 - 1)	0 (0 - 0.9)	0 (0 - 2)	0.2 (0 - 1.6)	0 (0 - 0.1)	0.3 (0 - 1.7)	0.2 (0 - 1.6)	0.2 (0 - 1.7)	0^{A} (0 - 0.7)	0^{A} (0 - 00.4)	0 (0 - 1.6)
Planctomycetia	0.0039	0.00724286	0.25 (0-1.7)	0.4 (0-1)	0 (0-0.9)	0 (0-2)	0.2 (0-1.6)	0 (0-0.1)	0.3 (0-1)	0.2 (0-1.6)	0.2 (0-1.7)	0^{A} (0-0.7)	0.2^{A} (0-0.4)	0 (0-1.6)
Gemmatales	0.0254	0.127	0.05 (0-1.3)	0.1 (0-1)	0 (0-0.7)	0 (0-1.1)	0.1 (0-1.2)	0 (0-0.1)	0.3 (0-0.5)	0.1 (0-0.7)	0.2 (0-1.4)	0 (0-0)	0 (0-0.4)	0 (0-1.4)
Gemmataceae	0.023	0.115	0 (0-0.6)	0 (0-0.5)	0 (0-0)	0 (0-0.7)	0 (0-0.5)	0 (0-0.1)	0 (0-0.3)	0 (0-0.3)	0.2^{C} (0-1)	0^{I} (0-0)	0 (0-0.1)	0 (0-0.8)
Isosphaeraceae	0.0979	1.11885714	0 (0-0.9)	0 (0-0.5)	0 (0-0.7)	0 (0-0.6)	0.05 (0-0.7)	0 (0-0.1)	0.1 (0-0.5)	0.1 (0-0.4)	0 (0-0.5)	0 (0-0)	0 (0-0.4)	0 (0-0.6)
Pirellulales	0.0006	0.00093103	0.15 (0-0.5)	0 (0-0.2)	0 (0-0.5)	0 (0-1)	0.05 (0-1.5)	0^{A} (0-0)	0 (0-0.4)	0 (0-0.9)	0 (0-0.5)	0^{A} (0-0.7)	0^{A} (0-0)	0^{A} (0-0.2)
Pirellulaceae	0.0006	0.00097959	0.15 (0-0.55)	0 (0-0.7)	0 (0-0.1)	0 (0-0.1)	0.05 (0-0.3)	0^{A} (0-0)	0 (0-0.4)	0 (0-0.1)	0 (0-0.2)	0^{A} (0-0)	0^{A} (0-0)	0^{A} (0-0)
Proteobacteria	0.1165	1.631	37.9 (18.9 - 56.5)	45 (17.8 - 78.9)	73.9 (27.7 - 97.8)	61.2 (10.9 - 99.8)	43.85 (23.8 - 70.8)	28.75 (3.4 - 90.1)	49.4 (27.2 - 99.4)	54.5 (6.8 - 98.1)	41.35 (2.4 - 96.9)	37.1 (7.1 - 94.7)	79.3 (18.3 - 96)	42.3 (1.5 - 70.4)
Alphaproteobacteria	0.0001	0.00011818	10.45 (3.9-26.9)	11 (4-26.6)	3.8 (0-13.8)	5.1 (0-24)	14.25 (2.9-26.3)	$0.35^{A, B, E}$ (0-4.3)	8.5^{F} (0.8-32.6)	6.3 (0-20.5)	9.5 (1.5-26.5)	$0.65^{A, B, E, G, I}$ (0-4.3)	$0.45^{A, B, E, G}$ (0-11.2)	4 (0.8-24.5)
Caulobacterales	0.0001	0.00010976	0.55 (0-1.2)	0.7 (0-1.8)	0.1 (0-2)	0.1 (0-3.3)	0.3 (0-1)	$0^{A, B}$ (0-0.2)	0.4 (0-0.9)	0.1 (0-1)	0.2 (0-0.9)	0^{A} (0-0.5)	$0^{A, B}$ (0-0.2)	0 (0-1.1)
Caulobacteraceae	0.0001	0.00011594	0.55 (0-1.2)	0.7 (0-1.8)	0.1 (0-2)	0.1 (0-3.3)	0.3 (0-1)	$0^{A, B}$ (0-0.2)	0.4 (0-0.9)	0.1 (0-1)	0.2 (0-0.9)	0^{A} (0-0.5)	$0^{A, B}$ (0-0.2)	0 (0-1.1)
Rhizobiales	0.0001	0.0001125	5.45 (1.4-11.3)	4.7 (1.8-12.8)	1.2 (0-5.2)	1.75 (0-12.5)	3.65 (0.4-12.6)	$0.25^{A, B, E}$ (0-2.4)	4.8 (0-18)	3.2 (0-8.1)	4.4 (0.3-10.9)	$0.3^{A, B, E}$ (0-4.1)	$0.2^{A, B}$ (0-4.9)	2.1 (0.4-22.8)
Aurantimonadaceae	0.0002	0.0002807	0.25 (0-1.3)	0.6 (0-2.1)	0 (0-0.7)	0 (0-0.6)	0.1 (0-1.7)	0 (0-0.4)	0.1 (0-3.7)	0.1 (0-0.5)	0.1 (0-1.1)	$0^{A, B}$ (0-0.1)	$0^{A, B}$ (0-0.3)	0 (0-0.5)
Bradyrhizobiaceae	0.0054	0.01270588	1.2 (0.2-3.4)	0.5 (0-5.1)	0.1 (0-1.9)	0.35 (0-3.6)	0.9 (0-3.3)	0.1 (0-0.2)	0.1 (0-4.6)	0.1 (0-2.9)	0.7 (0-5.7)	0.05 (0-3.7)	0^{A} (0-2.6)	0.2 (0-1.4)
Balneimonas	0.0066	0.01304286	0.85 (0.2-2.6)	0.3 (0-3.8)	0.1 (0-1.9)	0.3 (0-2.7)	0.65 (0-1.8)	0.1 (0-0.2)	0.1 (0-4.4)	0.1 (0-2.7)	0.35 (0-4.6)	0.05 (0-3.7)	0^{A} (0-0.2)	0.1 (0-1)
Bradyrhizobium	0.0547	0.26706471	0.1 (0-0.9)	0.1 (0-0.6)	0 (0-0.4)	0 (0-1.1)	0 (0-1.4)	0 (0-0.1)	0 (0-1.3)	0 (0-0.5)	0.05 (0-0.5)	0 (0-0.1)	0 (0-2.2)	0 (0-0.4)
Hyphomicrobiaceae	0.0001	0.00011765	0.5 (0.1-2.3)	0.4 (0-2.4)	0 (0-1.4)	0.05 (0-2.6)	0.85^{C} (0.1-3.2)	$0^{A, E}$ (0-0.1)	01.2^{F} (0-3.3)	0.5 (0-1.2)	0.55^{F} (0-1.8)	$0^{A, E}$ (0-0.5)	$0^{A, E}$ (0-0.5)	0.2 (0-0.7)
Devosia	0.0001	0.0001137	0.2 (0-0.7)	0.3 (0-0.9)	0 (0-0.4)	0 (0-1.6)	0.2 (0-2.2)	$0^{A, E}$ (0-0)	0.2^{F} (0-2.7)	0.2^{F} (0-1.2)	0.15 (0-0.6)	$0^{A, E, H}$ (0-0.1)	$0^{A, E, H}$ (0-0.1)	0.2 (0-0.6)
Rhodoplanes	0.0104	0.02158	0.1 (0-1.2)	0 (0-1.2)	0 (0-0.3)	0 (0-1.2)	0.15 (0-1.7)	0 (0-0.1)	0 (0-1.7)	0 (0-0.7)	0.25 (0-0.7)	0 (0-0.5)	0 (0-0.5)	0 (0-0.7)
Methylobacteriaceae	0.0001	0.0001194	0.75 (0.2-2.7)	0.8 (0.5-1.3)	0.1 (0-0.8)	0.2 (0-3.3)	0.75 (0.1-3.4)	$0^{B, E}$ (0-0.6)	0.7 (0-2.6)	0.3 (0-1.7)	0.6 (0-1.4)	$0^{A, B, E, G}$ (0-0.4)	$0^{A, B, E}$ (0-0.7)	0.2 (0-0.5)
Methylobacterium	0.0001	0.00011528	0.3 (0-1.8)	0.6 (0-0.8)	0.1 (0-0.3)	0.05 (0-1.6)	0.35 (0-2)	0^{B} (0-0.3)	0.2 (0-1.8)	0.1 (0-1.5)	0.3 (0-0.9)	$0^{A, B, E}$ (0-0.1)	$0^{B, E}$ (0-0.6)	0 (0-0.4)
Phyllobacteriaceae	0.0005	0.00075472	0.15 (0-0.7)	0.1 (0-0.7)	0 (0-0.3)	0 (0-0.6)	0.05 (0-0.9)	0 (0-0)	0.1^{F} (0-1.5)	0 (0-0.3)	0.05 (0-0.4)	$0^{A, G}$ (0-0)	0 (0-0.3)	0 (0-0.4)
Rhizobiaceae	0.0013	0.00266667	0.4 (0-1.7)	0.7 (0-2.3)	0 (0-1)	0.05 (0-2.1)	0.45 (0-1.5)	0^{A} (0-0.3)	0.7 (0-2.6)	0.1 (0-2.7)	0.3 (0-1.3)	0 (0-1.3)	0 (0-2.4)	0.1 (0-3.9)
Agrobacterium	0.0054	0.00996	0.25 (0-1.1)	0.6 (0-1.8)	0 (0-1)	0 (0-1.7)	0.3 (0-1.1)	0 (0-0)	0.3 (0-2)	0.1 (0-2.7)	0.1 (0-1)	0 (0-1.3)	0 (0-2.4)	0.1 (0-3.9)
Rhodobacterales	0.0001	0.00011538	1.35 (0.3-4.7)	0.9 (0.2-5.9)	0 (0-3.2)	0.2 (0-3.8)	1.1 (0.1-5.7)	$0.05^{A, E}$ (0-0.6)	0.5 (0-3.8)	0.2 (0-3)	0.95 (0-3)	$0^{A, E}$ (0-0.9)	$0^{A, B, E}$ (0-0.9)	0.2 (0-1)
Rhodobacteraceae	0.0001	0.00012121	1.35 (0.3-4.7)	0.9 (0.2-5.9)	0 (0-3.2)	0.2 (0-3.8)	1.1 (0.1-5.7)	$0.05^{A, E}$ (0-0.5)	0.5 (0-3.4)	0.2 (0-2.9)	0.95 (0-2.8)	$0^{A, E}$ (0-0.9)	$0^{A, B, E}$ (0-0.9)	0.2 (0-1)
Paracoccus	0.0005	0.00062879	0.25 (0-1.9)	0.5 (0-1)	0 (0-1.2)	0 (0-1)	0.15 (0-2.5)	$0^{A, B}$ (0-0.1)	0.1 (0-0.6)	0.1 (0-0.8)	0 (0-1.1)	0^{A} (0-0.4)	$0^{A, B}$ (0-0)	0 (0-0.5)
Rubellimicrobium	0.0025	0.00399038	0.5 (0-2.7)	0.2 (0-4.4)	0 (0-1.6)	0.1 (0-3.5)	0.45 (0.1-4.6)	0 (0-0.5)	0 (0-2.6)	0 (0-2.1)	0.1 (0-2.8)	0^{E} (0-0.5)	0^{E} (0-0.7)	0 (0-0.2)
Rhodospirillales	0.0001	0.00011842	0.95 (0-5.7)	0.6 (0.1-3.5)	0.2 (0-2.5)	0.5 (0-6.3)	1.6 (0.1-6.1)	0.05^{E} (0-0.3)	0.6 (0-3.4)	0.3 (0-4.6)	1.05^{F} (0.2-3.8)	$0^{E, I}$ (0-1.3)	$0^{E, I}$ (0-0.8)	0.2 (0-2.6)
Acetobacteraceae	0.0001	0.00012308	0.4 (0-1.9)	0.5 (0-2)	0 (0-0.9)	0.35 (0-2.3)	0.8 (0.1-1.5)	0^{E} (0-0.2)	0.6 (0-2.2)	0.2 (0-1.1)	0.55 (0-2.4)	0^{E} (0-0.4)	$0^{A, E, I}$ (0-0.1)	0.2 (0-1.2)
Roseomonas	0.0008	0.00107097	0.3 (0-0.7)	0.2 (0-1)	0 (0-0.4)	0.1 (0-1.9)	0.35 (0-1)	0 (0-0.2)	0.1 (0-2.1)	0.1 (0-0.7)	0.05 (0-1.4)	0 (0-0.4)	0^{E} (0-0)	0.1 (0-0.9)
Rhodospirillaceae	0.0076	0.02171429	0.2 (0-3.7)	0.2 (0-1.9)	0.2 (0-2.5)	0.1 (0-3.7)	0.65 (0-5.7)	0 (0-0.2)	0.2 (0-2.2)	0.1 (0-4)	0.6 (0-1.7)	0^{I} (0-1.2)	0 (0-0.7)	0 (0-1.7)
Skermanella	0.0182	0.04442941	0.15 (0-1.7)	0 (0-1.4)	0 (0-2.5)	0.1 (0-3.1)	0.25 (0-4.4)	0 (0-0.1)	0 (0-1.1)	0.1 (0-2.4)	0.45 (0-0.9)	0 (0-0.2)	0 (0-0.5)	0 (0-1.5)
Rickettsiales	0.0008	0.00138462	0.05 (0-0.9)	0 (0-18.3)	0 (0-0.3)	0 (0-0.5)	0.25^{C} (0-21.8)	0^{E} (0-0.1)	0 (0-0.2)	0 (0-0.9)	0.05 (0-1.6)	0^{E} (0-0)	0^{E} (0-0.2)	0 (0-0.2)
mitochondria	0.0028	0.00622222	0.05 (0-0.9)	0 (0-18.3)	0 (0-0.3)	0 (0-0.5)	0.2^{C} (0-21.8)	0^{E} (0-0.1)	0 (0-0.2)	0 (0-0.3)	0.05 (0-1.6)	0^{E} (0-0)	0 (0-0.2)	0 (0-0.2)
Sphingomonadales	0.0001	0.00012162	1.85 (0.7-9.6)	2.2 (0.7-4.8)	0.3 (0-2.4)	1.55 (0-7.4)	2.75 (0.2-7.7)	0.15 (0-1.3)	2.2^{F} (0.6-11.1)	1.2 (0-3.8)	1.8 (0.1-8.9)	$0^{A, B, E, G, I}$ (0-1.1)	$0^{A, B, E, G}$ (0-5.2)	1.1 (0-3.1)
Erythrobacteraceae	0.0011	0.00209524	0.2 (0-0.8)	0.1 (0-0.4)	0 (0-0.1)	0 (0-0.9)	0 (0-0.6)	0 (0-0.1)	0.2 (0-1)	0 (0-0.1)	0.15 (0-0.6)	0 (0-0.4)	0 (0-0.3)	0 (0-0.3)
Sphingomonadaceae	0.0001	0.000125	1.55 (0.5-8.6)	1.6 (0.7-4.4)	0.3 (0-2.3)	1.45 (0-7.1)	2.75 (0.2-7.1)	0.15 (0-1.1)	1.4 (0-9.5)	1.1 (0-3.6)	1.65 (0.1-8.8)	$0^{A, B, E, G, I}$ (0-0.6)	$0^{A, B, E, G}$ (0-4.9)	0.8 (0-3.1)
Kaistobacter	0.0165	0.03804167	0.2 (0-1.7)	0 (0-0.3)	0 (0-0.4)	0.15 (0-2.9)	0.1 (0-3.7)	0 (0-0.1)	0.1 (0-1.4)	0.1 (0-0.6)	0 (0-3.4)	0 (0-0.2)	0 (0-0.1)	0 (0-0.5)
Novosphingobium	0.0139	0.03036053	0.1 (0-0.5)	0 (0-0.2)	0 (0-0)	0 (0-0.7)	0 (0-0.4)	0 (0-0.1)	0 (0-0.6)	0 (0-0.1)	0 (0-0.1)	0 (0-0.1)	0 (0-0)	0 (0-1.7)
Sphingomonas	0.0001	0.0001169	0.35 (0.1-4.4)	0.8 (0-2.4)	0.2 (0-1.7)	0.55 (0-2.7)	0.3 (0.1-2.4)	0 (0-1)	0.7 (0-6)	0.5 (0-2.1)	0.15 (0-1.9)	$0^{A, B, E, G}$ (0-0.5)	$0^{A, B, E, G}$ (0-4.7)	0.2 (0-1.7)
Betaproteobacteria	0.1468	1.9084	9.95 (2-39.5)	18.2 (5.8-70.4)	23.5 (0-85.8)	25 (1.4-99.8)	12.5 (3.6-47.6)	5.4 (0.2-49.4)	19.4 (2.3-94.4)	14.9 (2.1-97.9)	11.45 (0.6-82.3)	7.45 (0.1-38.9)	20.45 (0.1-95.3)	8.2 (0-65.7)
Burkholderiales	0.0469	0.4221	7.6 (1.1-35.7)	12.1 (4.1-69.2)	22.1 (0-85.5)	24.7 (1.3-99.8)	8.8 (0.6-45.7)	5.3 (0.2-47.5)	19.1 (1.6-94.4)	6.8 (0.9-97.9)	10.95 (0.6-82.3)	3.35 (0.1-37.8)	19.9 (0-95.3)	4.6 (0-65.5)
Alcaligenaceae	0.0001	0.00012698	0 (0-0.4)	0 (0-0.2)	0 (0-0)	0 (0-0.7)	0 (0-0.7)	$0.7^{A, B, C, D, E}$ (0-7.3)	0^{F} (0-0.1)	0^{F} (0-2)	0^{F} (0-0.1)	0^{F} (0-0)	0^{F} (0-1.2)	0^{F} (0-0.1)
Sutterella	0.0001	0.00011857	0 (0-0.4)	0 (0-0.2)	0 (0-0)	0 (0-0.7)	0 (0-0.4)	$0.7^{A, B, C, D, E}$ (0-7.3)	0^{F} (0-0.1)	0^{F} (0-2)	0^{F} (0-0)	0^{F} (0-0)	0^{F} (0-1.2)	0^{F} (0-0)
Burkholderiaceae	0.0101	0.03366667	0.05 (0-0.9)	0.2 (0-0.7)	0 (0-0.5)	0 (0-1.4)	0.35 (0-1.2)	0 (0-1.3)	0 (0-1.3)	0.3 (0-1.6)	0 (0-0.2)	0.05 (0-0.7)	0 (0-0.4)	0 (0-0.1)
Lautropia	0.1339	1.01033636	0.075 (0-0.5)	0.06 (0-0.2)	0.009 (0-0.3)	0.08 (0-0.9)	0.15 (0-1.2)	0.03 (0-0.7)	0.02 (0-0.1)	0.02 (0-0.5)	0.18 (0-0.1)	0.02 (0-0.7)	0 (0-0.2)	0 (0-0.1)
Comamonadaceae	0.0168	0.07466667	0.7 (0-2.7)	0.7 (0-1.9)	0.3 (0-1.6)	0.25 (0-2.6)	0.7 (0.1-17.8)	0 (0-1)	0.4 (0.1-4.5)	1 (0-3.8)	0.45 (0-3.3)	0.35 (0-4.1)	0 (0-2.5)	0.2 (0-1.8)
Azohydromonas	0.1706	1.41598	0 (0-0.4)	0 (0-0.3)	0 (0-0.1)	0 (0-1)	0 (0-0.7)	0 (0-0.3)	0 (0-0.2)	0 (0-0.3)	0 (0-1.5)	0 (0-0.2)	0 (0-0)	0 (0-0)
Diaphorobacter	0.3217	4.45018333	0 (0-0.7)	0 (0-0.2)	0 (0-1)	0 (0-0.2)	0 (0-0.6)	0 (0-0.6)	0 (0-2.2)	0 (0-0)	0 (0-0.1)	0 (0-0.1)	0 (0-1.2)	0 (0-0.1)
Oxalobacteraceae	0.0092	0.02725926	6.25 (0.5-34.7)	10 (3.5-68.4)	21.9 (0-85)	24.25 (0.6-99.6)	2.95 (0.5-42.5)	0.65 (0-45.8)	18.7 (1.1-93.9)	3 (0-97.9)	9.15 (0.6-82.3)	1.2^{C} (0-37.4)	19.9 (0-95.2)	3 (0-65.5)
Janthinobacterium	0.023	0.06158065	0 (0-0.5)	0 (0-0.3)	0 (0-0.1)	0 (0-0.5)	0 (0-0.1)	0 (0-0.1)	0 (0-0.3)	0 (0-0)	0.05^{H} (0-2.5)	0^{I} (0-0)	0 (0-0.1)	0 (0-0)
Ralstonia	0.0298	0.08833571	4.25 (0.2-29.8)	9.1 (1-56.3)	18.4 (0-76.5)	19.75 (0.2-84.6)	1.45 (0-34.9)	0.35 (0-44.9)	15.2 (0-76.9)	2 (0-84.2)	7 (0.1-68.3)	1.05 (0-33.2)	16.95 (0-83.8)	2.3 (0-56.2)
Neisseriales	0.0001	0.000125	1.45 (0-6.7)	2 (0.2-9.5)	0.2 (0-1.2)	0.1 (0-1.3)	$2.9^{C, D}$ (0.8-6)	0.05^{E} (0-32.4)	0.1^{E} (0-5.8)	2.3 (0-14.3)	0.3^{E} (0-1.2)	1.25 (0-13.5)	0.15^{E} (0-2.9)	0.2 (0-4.9)
Neisseriaceae	0.0001	0.00012903	1.45 (0-6.7)	2 (0.2-9.5)	0.2 (0-1.2)	0.1 (0-1.3)	2.9^{D} (0.8-6)	0.05^{E} (0-32.4)	0.1^{E} (0-5.8)	2.3 (0-14.3)	0.3^{E} (0-1.2)	1.25 (0-13.5)	0.15^{E} (0-2.9)	0.2 (0-4.9)
Conchiformibius	0.0001	0.00012029	0.4 (0-2.6)	0.8 (0.1-7.8)	0 (0-0.3)	0 (0-0.6)	$1.7^{C, D}$ (0-4.2)	0^{E} (0-16)	0.1 (0-1.3)	0.6 (0-8.7)	0^{E} (0-0.7)	0.65 (0-9.2)	0 (0-2.4)	0 (0-4.2)
Deltaproteobacteria	0.0069	0.0138	0.2 (0-4.2)	0.1 (0-7.1)	0 (0-2.1)	0.3 (0-1.9)	0.6 (0-3.1)	0 (0-1.3)	0.1 (0-12.3)	0.2 (0-1.6)	0.7 (0-1.4)	0.1 (0-2)	0^{E} (0-0.2)	0 (0-14.1)
Desulfovibrionales	0.0001	0.00012857	0 (0-0)	0 (0-0)	0 (0-0)	0 (0-0)	0 (0-0.5)	0 (0-0)	0 (0-0.6)	0 (0-0.3)	0 (0-0.1)	$0.1^{A, B, C, D, F, G, H, I}$ (0-2	0^{J} (0-0.2)	0 (0-0.1)
Desulfomicrobiaceae	0.0002	0.00028571	0 (0-0)	0 (0-0)	0 (0-0)	0 (0-0)	0 (0-0.3)	0 (0-0)	0 (0-0.6)	0 (0-0.3)	0 (0-0.1)	$.1^{A, B, C, D, F, G, H, I}$ (0-1.9	0^{J} (0-0.2)	0^{J} (0-0.1)
Myxococcales	0.0001	0.00013235	0.1 (0-3.7)	0.1 (0-6.8)	0 (0-0.8)	0.25 (0-1.9)	0.25 (0-3.1)	0 (0-1)	0 (0-12.1)	0 (0-1.1)	0.5 (0-1.1)	$0^{D, E, I}$ (0-0)	$0^{E, I}$ (0-0.1)	0 (0-14.1)
Spirobacillales	0.101	1.515	0.05 (0-1)	0 (0-0.1)	0 (0-0.5)	0 (0-0.7)	0 (0-0.7)	0 (0-0.5)	0 (0-0.4)	0 (0-0.5)	0 (0-0.5)	0 (0-0)	0 (0-0)	0 (0-0.3)
Epsilonproteobacteria	0.0221	0.06384444	0 (0-0.2)	0 (0-0.4)	0 (0-90.3)	0 (0-0)	0 (0-0.5)	0.1 (0-3.7)	0 (0-0.2)	0 (0-0.9)	0 (0-0.2)	0.15^{D} (0-0.5)	0 (0-3.3)	0 (0-9.3)
Campylobacterales	0.0221	0.09040909	0 (0-0.2)	0 (0-0.4)	0 (0-90.3)	0 (0-0)	0 (0-0.5)	0.1 (0-3.7)	0 (0-0.2)	0 (0-0.9)	0 (0-0.2)	0.15 (0-0.5)	0 (0-3.3)	0 (0-9.3)

Gammaproteobacteria	0.0035	0.0056875	6.85 (1.5-29.2)	10 (1.3-23.1)	1.6 (0.5-90.4)	2.65 (0-83.3)	9 (2.4-51.6)	4.65 (0.1-89.3)	2.8 (0-14.3)	11.2 (0.2-39)	6.9 (0.2-88.1)	23.5[C, D, G] (4.7-93.5)	38.5 (0.4-92.5)	6.9 (0.2-49.1)
Cardiobacteriales	0.1549	3.48525	0 (0-0.2)	0 (0-0.2)	0 (0-0.2)	0 (0-0.1)	0 (0-0.8)	0 (0-0.2)	0 (0-0)	0.2 (0-0.6)	0 (0-0.9)	0 (0-0.2)	0 (0-0.3)	0 (0-0.3)
Enterobacteriales	0.0001	0.00013636	0.95 (0-5.3)	1.4 (0.4-3)	0.2 (0-12.5)	0.35 (0-13.4)	0.5 (0-7)	0.15 (0-88.4)	0.7 (0-3.7)	0.6 (0.1-4.4)	0.55 (0-10)	0[B] (0-0.4)	0[A, B, G, H] (0-0.1)	0.3 (0-25.6)
Enterobacteriaceae	0.0001	0.00013115	0.95 (0-5.3)	1.4 (0.4-3)	0.2 (0-12.5)	0.35 (0-13.4)	0.5 (0-7)	0.15 (0-88.4)	0.7 (0-3.7)	0.6 (0.1-4.4)	0.55 (0-10)	0[B] (0-0.4)	0[A, B, G, H] (0-0.1)	0.3 (0-25.6)
Erwinia	0.0008	0.00108852	0.2 (0-2.8)	0.5 (0-0.7)	0 (0-0.4)	0 (0-0.7)	0.05 (0-1)	0 (0-0.2)	0.2 (0-1.3)	0.1 (0-1)	0 (0-2.7)	0[A, B] (0-0)	0 (0-0.1)	0 (0-0.2)
Klebsiella	0.0361	0.12484583	0 (0-0.4)	0 (0-0.2)	0 (0-10.9)	0 (0-0.2)	0 (0-0.3)	0 (0-0.1)	0 (0-0.3)	0 (0-0.2)	0 (0-0.5)	0 (0-0.1)	0 (0-0)	0.1 (0-10.7)
Trabulsiella	0.0344	0.10981538	0.05 (0-0.3)	0 (0-0.4)	0 (0-0.6)	0 (0-0.6)	0 (0-1.3)	0 (0-0)	0 (0-0.8)	0 (0-0.1)	0 (0-0.3)	0 (0-0)	0 (0-0)	0 (0-0.1)
Halomonas	0.1207	0.83484167	0 (0-1.1)	0 (0-0.4)	0 (0-0)	0 (0-0.7)	0 (0-0.1)	0 (0-0)	0 (0-0.1)	0 (0-0)	0 (0-0.6)	0 (0-0)	0 (0-0)	0 (0-0)
Pasteurellales	0.0001	0.00014063	0.45 (0-26.2)	1 (0-7.2)	0.1 (0-2.2)	0 (0-1.7)	1.3 (0.1-3.6)	0.75 (0-22.1)	0.2 (0-2.9)	1 (0-13.2)	0.3 (0-3)	13.4[C, D, G, I] (1-75.3)	0.05[J] (0-11.3)	0.1[J] (0-1.7)
Pasteurellaceae	0.0001	0.00013333	0.45 (0-26.2)	1 (0-7.2)	0.1 (0-2.2)	0 (0-1.7)	1.3 (0.1-3.6)	0.75 (0-22.1)	0.2 (0-2.9)	1 (0-13.2)	0.3 (0-3)	13.4[C, D, G, I] (1-75.3)	0.05[J] (0-11.3)	0.1[J] (0-1.7)
Pseudomonadales	0.0006	0.00096429	1.9 (0.1-8.4)	5.3 (0.3-9.8)	0.6 (0.1-77.4)	0.6 (0-2.7)	3.55 (0.7-50.5)	0.45 (0-4.9)	0.8 (0-5.9)	4 (0-28.5)	1.95 (0-84)	3.75 (0.1-91.2)	34.25[D] (0-92.1)	1.3 (0.1-36.2)
Moraxellaceae	0.0001	0.00013559	0.95 (0-8.3)	3.6 (0.2-5.5)	0.1 (0-0.6)	0.1 (0-1.3)	3.15[C] (0.3-50.5)	0.1[E] (0-1)	0.3 (0-2.6)	2.3 (0-28.2)	1.1 (0-83.6)	3.15[C, D, F] (0.1-18.4)	33.1[C, D, F] (0-92)	0.9 (0-35.7)
Acinetobacter	0.0765	0.4233	0 (0-0.5)	0.1 (0-0.6)	0 (0-0.3)	0 (0-0.1)	0.1 (0-1)	0 (0-0.1)	0 (0-0.2)	0 (0-0.3)	0.1 (0-2.6)	0.05 (0-2.2)	0 (0-0.5)	0.1 (0-31.8)
Enhydrobacter	0.0036	0.006225	0.1 (0-2.7)	0.2 (0-1.3)	0 (0-0.2)	0 (0-0.9)	0.1 (0-2)	0 (0-0.2)	0 (0-0.2)	0 (0-1.1)	0 (0-1.3)	0.3[C] (0-13.6)	0 (0-0.4)	0.1 (0-1.9)
Moraxella	0.0997	0.59107857	0 (0-0.1)	0 (0-0.1)	0 (0-0)	0 (0-0)	0.05 (0-1)	0 (0-0.2)	0 (0-0.2)	0 (0-0.3)	0 (0-0.1)	0 (0-7.7)	0 (0-0.4)	0 (0-0.7)
Pseudomonadaceae	0.0267	0.1424	0.5 (0-5.1)	0.6 (0.1-9)	0.3 (0-77.4)	0.2 (0-1.4)	0.45 (0-1.6)	0.25 (0-4.1)	0.4 (0-3.3)	0.4 (0-4.5)	0.4 (0-1.5)	0 (0-90)	0.05 (0-2.1)	0.2 (0-31.7)
Pseudomonas	0.0431	0.178865	0.25 (0-4.6)	0.2 (0-6.3)	0.3 (0-74.7)	0.05 (0-1.1)	0.3 (0-0.9)	0 (0-0.6)	0.2 (0-2.6)	0.1 (0-4.5)	0.2 (0-1.5)	0 (0-89.5)	0.05 (0-0.3)	0.1 (0-30.6)
Xanthomonadales	0.0001	0.00014516	0.55 (0.1-14.2)	0.7 (0.1-13)	0.3 (0-6.6)	0.2 (0-82.6)	0.6 (0.1-9.2)	0[A, B, E] (0-0.5)	0.8 (0-3.1)	0.4 (0-15.8)	0.6 (0-2.8)	0.1 (0-2.2)	0[A, B, E] (0-1)	0.2 (0-5.3)
Xanthomonadaceae	0.0001	0.00013793	0.5 (0.1-14.1)	0.7 (0.1-13)	0.1 (0-6.6)	0.2 (0-82.6)	0.6 (0.1-9.2)	0[A, B, E] (0-0.5)	0.6 (0-3.1)	0.3 (0-15.8)	0.55 (0-2.8)	0.1 (0-2.2)	0[A, B, E] (0-1)	0.2 (0-5.3)
Luteimonas	0.0154	0.03454595	0 (0-0.4)	0.1 (0-1.2)	0 (0-0)	0 (0-0.8)	0 (0-0.5)	0 (0-0)	0 (0-0.9)	0 (0-1.1)	0 (0-0.3)	0 (0-0)	0 (0-0.1)	0 (0-0.1)
Lysobacter	0.1206	0.76998462	0.05 (0-1)	0 (0-0.4)	0 (0-0.1)	0 (0-1.4)	0 (0-0.4)	0 (0-0.1)	0 (0-0.5)	0 (0-0.1)	0 (0-1.1)	0 (0-0.2)	0 (0-0)	0 (0-0.3)
Stenotrophomonas	0.5885	48.8455	0 (0-0.6)	0 (0-1.1)	0 (0-0.5)	0 (0-0.8)	0 (0-0.2)	0 (0-0)	0 (0-0.6)	0 (0-0.8)	0 (0-0.2)	0 (0-0.2)	0 (0-0.5)	0 (0-4.5)
Spirochaetes	0.007	0.0098	0.1 (0 - 0.3)	0.2 (0 - 0.5)	0 (0 - 0.4)	0 (0 - 0)	0 (0 - 2.9)	0 (0 - 0.2)	0 (0 - 0.1)	0 (0 - 2.4)	0 (0 - 0.4)	0.45[D, G] (0 - 13)	0 (0 - 0.9)	0 (0 - 1.1)
Spirochaetes	0.0008	0.00104	0.1 (0-0.3)	0.1 (0-0.5)	0 (0-0.4)	0 (0-0)	0 (0-0.3)	0 (0-0.2)	0 (0-0.1)	0 (0-2.3)	0 (0-0.4)	0.45[D, G] (0-13)	0 (0-0.9)	0 (0-1.1)
Spirochaetales	0.0008	0.00144	0.1 (0-0.3)	0.1 (0-0.5)	0 (0-0.4)	0 (0-0)	0 (0-2.9)	0 (0-0.2)	0 (0-0.1)	0 (0-2.3)	0 (0-0.4)	0.4[E, G] (0-13)	0 (0-0.009)	0 (0-0.011)
Spirochaetaceae	0.0008	0.0013913	0.1 (0-0.3)	0.1 (0-0.5)	0 (0-0.4)	0 (0-0)	0 (0-2.9)	0 (0-0.2)	0 (0-0.1)	0 (0-2.3)	0 (0-0.4)	0.4[D, G] (0-13)	0 (0-0.9)	0 (0-1.1)
Treponema	0.0008	0.00110667	0.1 (0-0.3)	0.1 (0-0.5)	0 (0-0.4)	0 (0-0)	0 (0-2.9)	0 (0-0.2)	0 (0-0.1)	0 (0-2.3)	0 (0-0.4)	0.4[D, G] (0-13)	0 (0-0.9)	0 (0-1.1)
Tenericutes	0.0112	0.0196	0 (0 - 0.6)	0.1 (0 - 21.9)	0 (0 - 0.2)	0 (0 - 0.6)	0 (0 - 2.8)	0 (0 - 15.6)	0 (0 - 0.3)	0.1 (0 - 13.2)	0 (0 - 6.4)	1.1[C, D] (0 - 12.9)	0 (0 - 3)	0 (0 - 1.3)
Mollicutes	0.0112	0.02912	0 (0-0.6)	0.1 (0-21.9)	0 (0-0.2)	0 (0-0.6)	0 (0-2.8)	0 (0-15.6)	0 (0-0.3)	0.1 (0-13.2)	0 (0-6.4)	1.1[C, D] (0-12.9)	0 (0-3)	0 (0-1.3)
Acholeplasmatales	0.0014	0.003	0 (0-0.1)	0 (0-0.1)	0 (0-0)	0 (0-0.6)	0 (0-0.6)	0 (0-0)	0 (0-0.1)	0 (0-1.3)	0 (0-0.1)	0.15[A, C, D, E, F] (0-11.5)	0[J] (0-3)	0 (0-0.3)
Acholeplasmataceae	0.0014	0.00294737	0 (0-0.1)	0 (0-0.1)	0 (0-0)	0 (0-0.6)	0 (0-0.6)	0 (0-0)	0 (0-0.1)	0 (0-1.3)	0 (0-0.1)	0.15[A, C, D, E, F] (0-11.5)	0[J] (0-3)	0 (0-0.3)
Acholeplasma	0.0014	0.00211273	0 (0-0.1)	0 (0-0.1)	0 (0-0)	0 (0-0.6)	0 (0-0.6)	0 (0-0)	0 (0-0.1)	0 (0-1.3)	0 (0-0.1)	0.15[A, C, D, E, F] (0-11.5)	0[J] (0-3)	0 (0-0.3)
Mycoplasmatales	0.0128	0.048	0 (0-0.6)	0 (0-21.9)	0 (0-0.2)	0 (0-0.1)	0 (0-2.8)	0 (0-15.6)	0 (0-0.3)	0 (0-12.7)	0 (0-6.4)	0.8 (0-6.4)	0 (0-0.2)	0 (0-1.2)
Mycoplasmataceae	0.0128	0.04452174	0 (0-0.6)	0 (0-21.9)	0 (0-0.2)	0 (0-0.1)	0 (0-2.8)	0 (0-15.6)	0 (0-0.3)	0 (0-12.7)	0 (0-6.4)	0.8[A, C, D] (0-6.4)	0 (0-0.2)	0 (0-1.2)
Mycoplasma	0.0011	0.00154746	0 (0-0.1)	0 (0-1.1)	0 (0-0)	0 (0-0.1)	0 (0-2.4)	0 (0-15.6)	0 (0-0.3)	0 (0-9.7)	0 (0-5.5)	0.3[A, C, D] (0-3.2)	0[J] (0-0.2)	0[J] (0-1)
Thermi	0.0417	0.14595	0 (0 - 0.3)	0 (0 - 0.3)	0 (0 - 0.4)	0 (0 - 0.1)	0.05 (0 - 0.2)	0 (0 - 0)	0 (0 - 0.2)	0 (0 - 0.1)	0 (0 - 0.4)	0 (0 - 0)	0 (0 - 0.1)	0.1 (0 - 0.3)
Deinococci	0.0417	0.135525	0 (0-4.2)	0 (0-2.2)	0 (0-0.3)	0 (0-0)	0 (0-0.3)	0 (0-4.6)	0 (0-0.1)	0 (0-0.5)	0.1 (0-1)	0 (0-0.5)	0 (0-0.1)	0 (0-0.4)
Deinococcales	0.0417	0.26807143	0 (0-4.2)	0 (0-2.2)	0 (0-0.3)	0 (0-0)	0 (0-0.3)	0 (0-4.6)	0 (0-0.1)	0 (0-0.5)	0.1 (0-1)	0 (0-0.5)	0 (0-0.1)	0 (0-0.4)
Deinococcaceae	0.0417	0.25661538	0 (0-4.2)	0 (0-2.2)	0 (0-0.3)	0 (0-0)	0 (0-0.3)	0 (0-4.6)	0 (0-0.1)	0 (0-0.5)	0.1 (0-1)	0 (0-0.5)	0 (0-0.1)	0 (0-0.4)
Deinococcus	0.0417	0.16481429	0 (0-4.2)	0 (0-2.2)	0 (0-0.3)	0 (0-0)	0 (0-0.3)	0 (0-4.6)	0 (0-0.1)	0 (0-0.5)	0.1 (0-1)	0 (0-0.5)	0 (0-0.1)	0 (0-0.4)
Verrucomicrobia	0.0206	0.05768	0 (0 - 4.2)	0 (0 - 2.2)	0 (0 - 0.3)	0 (0 - 0)	0 (0 - 0.3)	0 (0 - 4.6)	0 (0 - 0.1)	0 (0 - 0.5)	0.1 (0 - 1)	0 (0 - 0.5)	0 (0 - 0.1)	0 (0 - 0.4)

Taxa present in at least 50% of dogs in at least one of the skin sites.

*q-values adjusted based on the Benjamini and Hochberg False discovery rate.

Superscripts represent sites that were significantly different when compared to the skin sites in each column. A: Axilla; B: Concave pinna; C:Conjunctiva; D:Dorsal lumbar: E: Dorsal nose; F: Dorsal perianal; G: Ear; H: Groin; I: Interdigital skin between digits 4 & 5; L: Lip commissure; K: Nostril: L: Periocular.
