# Supplementary material for: The Skin Microbiome in Healthy and Allergic Dogs
Source: PLoS One. 2014 Jan 8;9(1):e83197. doi: 10.1371/journal.pone.0083197 (PMC3885435; doi:10.1371/journal.pone.0083197)
Supplement: Table S2 — Relative percentages of the most abundant bacterial groups on the different skin sites in the allergic versus healthy dogs at the various phylogenetic levels (phylum, class, order, family, genus) based on pyrosequencing. (PDF) [file pone.0083197.s002.pdf]

Table S2. Relative percentages of the most abundant bacterial groups on the different skin sites in the allergic versus healthy dogs at the various phylogenetic levels (phylum, class, order, family, genus) based on pyrosequencing.

Taxon	p-value	q-value**	Median % (Min-Max%)							
			Axilla		Groin		Interdigit45		Nostril	
			Allergic	Healthy	Allergic	Healthy	Allergic	Healthy	Allergic	Healthy
Chloracidobacteria	0.2027	0.310011765	0 (0-0.2)	0.1 (0-2.4)	0 (0-1.4)	0 (0-1.7)	0 (0-0.3)	0 (0-1.7)	0 (0-0)	0 (0-0.2)
Actinobacteria	0.4825	1.125833333	6.2 (0.6-19.1)	20.95 (1.5-51.5)	17.9 (3.1-95.9)	8.1 (0.2-38.3)	24 (16.7-94.8)	21.25 (1-94)	0.85 (0-27.2)	2.05 (0-78.5)
Acidimicrobiia	0.5613	2.91876	0 (0-0.2)	0 (0-0.7)	0 (0-0.6)	0 (0-0.4)	0 (0-0.6)	0.05 (0-1)	0 (0-0)	0 (0-0)
Acidimicrobiales	0.5613	2.104875	0 (0-0.2)	0 (0-0.7)	0 (0-0.6)	0 (0-0.4)	0 (0-0.6)	0.05 (0-1)	0 (0-0)	0 (0-0)
Actinobacteria	0.444	1.443	5.7 (0.6-16.9)	15.1 (1.5-48.1)	10.7 (2-95.9)	7.8 (0.2-34.1)	24 (6.7-94.8)	14.2 (0.5-93.5)	0.8 (0-27.2)	1.45 (0-78.5)
Actinomycetales	0.4301	1.07525	5.7 (0.6-16.8)	14.9 (1.5-48.1)	10.7 (2-95.9)	7.8 (0.2-34)	24 (6.7-94.8)	14.1 (0.5-93.5)	0.8 (0-27.2)	1.45 (0-78.5)
Actinomycetaceae	0.2672	0.548102564	0.1 (0-0.2)	0.05 (0-1)	0 (0-0.2)	0 (0-0.4)	0 (0-0)	0 (0-0.4)	0 (0-0.9)	0 (0-3.6)
Actinomyces	0.1671	0.252169091	0 (0-0.1)	0 (0-0.2)	0 (0-0)	0 (0-0.2)	0 (0-0)	0 (0-0.2)	0 (0-0)	0 (0-3.6)
Corynebacteriaceae	0.1495	0.221481481	0.3 (0-0.4)	0.85 (0-31)	0 (0-95.9)	0.9 (0-5.2)	0.7 (0-71)	0.25 (0-91.3)	0 (0-0.1)	0.15 (0-77.6)
Corynebacterium	0.1495	0.217692982	0.3 (0-0.4)	0.85 (0-31)	0 (0-95.9)	0.9 (0-5.2)	0.7 (0-71)	0.25 (0-91.3)	0 (0-0.1)	0.15 (0-77.6)
Frankiaceae	0.0336	0.0384	0 (0-0)	0.05 (0-0.3)	0 (0-0)	0 (0-0.2)	0 (0-0.1)	0 (0-0.3)	0 (0-0)	0 (0-0)
Geodermatophilaceae	0.576	2.194285714	0 (0-0.4)	0.05 (0-0.5)	0 (0-0.1)	0 (0-0.4)	0 (0-0.1)	0.25 (0-0.7)	0 (0-16.5)	0 (0-0.2)
Intrasporangiaceae	0.9394	12.52533333	0 (0-0.4)	0.35 (0-4.5)	0.3 (0-1.3)	0.1 (0-2.1)	0.3 (0-1.1)	0.25 (0-1.1)	0 (0-0)	0 (0-0)
Kineosporiaceae	0.4575	1.464	0 (0-0.5)	0.1 (0-1.1)	0 (0-0.3)	0 (0-0.2)	0.2 (0-0.9)	0.05 (0-0.4)	0 (0-0)	0 (0-0)
Kineococcus	0.256	0.518243902	0 (0-0.5)	0.05 (0-0.9)	0 (0-0.2)	0 (0-0.2)	0.2 (0-0.8)	0 (0-0.4)	0 (0-0)	0 (0-0)
Microbacteriaceae	0.7807	6.2456	1 (0.6-5)	0.8 (0.3-4.2)	2.1 (0-4.4)	1 (0-4.4)	0.3 (0-6.5)	0.6 (0.1-3.8)	0 (0-0)	0.05 (0-4.3)
Curtobacterium	0.097	0.127793651	0.8 (0.4-2.4)	0.2 (0-1.3)	0.1 (0-2.1)	0.1 (0-0.6)	0 (0-4.3)	0 (0-0.2)	0 (0-0)	0 (0-0)
Leucobacter	0.4417	1.357818519	0.2 (0-0.2)	0.15 (0-1.2)	0.4 (0-1.2)	0.1 (0-3.3)	0 (0-1.3)	0 (0-0.4)	0 (0-0)	0 (0-4.3)
Microbacterium	0.7144	3.70595	0.2 (0-1.2)	0.1 (0-1.3)	0.2 (0-1.5)	0.2 (0-0.3)	0 (0-0.7)	0 (0-1.4)	0 (0-0)	0 (0-0.6)
Micrococcaceae	0.1335	0.181016949	0.5 (0-1.1)	0.4 (0-4.6)	0.1 (0-0.1)	0.2 (0-1.6)	0* (0-0.2)	0.5 (0-3.6)	0 (0-9.7)	0 (0-1.3)
Kocuria	0.0487	0.057744286	0 (0-0)	0 (0-0.1)	0 (0-0)	0 (0-0.1)	0 (0-0)	0 (0-0.3)	0 (0-0)	0 (0-0.7)
Micrococcus	0.3161	0.771655882	0.5 (0-0.9)	0.35 (0-4.4)	0 (0-0.1)	0 (0-0.9)	0 (0-0.2)	0.45 (0-2)	0 (0-9.3)	0 (0-0.5)
Micromonosporaceae	0.0338	0.039188406	0 (0-0)	0.1 (0-0.4)	0 (0-0.1)	0 (0-0.3)	0 (0-0.7)	0.1 (0-1.7)	0 (0-0)	0 (0-0.1)
Mycobacteriaceae	0.1878	0.306612245	0 (0-0.7)	1 (0.1-4)	0.6 (0-3.3)	0.3 (0-8.4)	0 (0-3.9)	0.4 (0.1-6.3)	0 (0-0)	0 (0-2.6)
Mycobacterium	0.1878	0.305635294	0 (0-0.7)	1 (0.1-4)	0.6 (0-3.3)	0.3 (0-8.4)	0 (0-3.9)	0.4 (0.1-6.3)	0 (0-0)	0 (0-2.6)
Nocardiaceae	0.7806	5.677090909	0.8 (0-2.1)	0.35 (0-6.6)	0.3 (0-5.3)	0 (0-11.5)	0 (0-8.4)	0.1 (0-2.8)	0 (0-0.1)	0 (0-0.4)
Rhodococcus	1	83	0.8 (0-1.9)	0.35 (0-6.6)	0.3 (0-5.3)	0 (0-11.5)	0 (0-8.4)	0.1 (0-2.8)	0 (0-0.1)	0 (0-0.4)
Nocardioidaceae	0.6018	3.009	0.2 (0-2.9)	2.25 (0-9.1)	0.9 (0-3.8)	0.6 (0-2.1)	1.3 (0-2.4)	1 (0-5.8)	0 (0-1.1)	0 (0-0.3)
Aeromicrobium	0.1088	0.150506667	0 (0-0.5)	0.35 (0-2.4)	0 (0-0)	0 (0-0.4)	0 (0-1.2)	0.05 (0-1)	0 (0-0)	0 (0-0)
Friedmanniella	0.2826	0.633940541	0 (0-0.8)	0.1 (0-1.1)	0 (0-1.2)	0 (0-0.6)	0 (0-0.8)	0.05 (0-1)	0 (0-0)	0 (0-0.3)
Nocardioides	0.7643	4.229126667	0 (0-0.4)	0.1 (0-1.1)	0.1 (0-0.5)	0 (0-0.2)	0 (0-0.2)	0.05 (0-0.8)	0 (0-0)	0 (0-0)
Propionibacteriaceae	0.0937	0.120903226	1.3 (0-1.6)	0.85 (0.1-5.1)	0.4 (0-0.6)	0.5 (0-5.5)	0 (0-67.5)	0.6 (0-3.1)	0 (0-0)	0.1 (0-1.1)
Microlunatus	0.2164	0.382153191	0 (0-0.1)	0.3 (0-0.6)	0 (0-0.4)	0 (0-0.3)	0 (0-0)	0 (0-0.8)	0 (0-0)	0 (0-0)
Propionibacterium	0.1958	0.325028	1.3 (0-1.5)	0.3 (0-4.8)	0.2 (0-0.6)	0.3 (0-5)	0 (0-59.9)	0.2 (0-3.1)	0 (0-0)	0.1 (0-1.1)
Pseudonocardiaceae	0.1993	0.332166667	0.3 (0-1.3)	0.5 (0-1.1)	0 (0-0.8)	0 (0-1.2)	0 (0-1.4)	0.4 (0-2.2)	0 (0-0)	0 (0-0.9)
Actinomycetospora	0.4495	1.434942308	0.1 (0-1.3)	0 (0-0.2)	0 (0-0.8)	0 (0-0.1)	0 (0-1.4)	0 (0-0.2)	0 (0-0)	0 (0-0)
Pseudonocardia	0.0323	0.036724658	0 (0-0.2)	0.1 (0-0.8)	0 (0-0.1)	0 (0-1.1)	0 (0-0.1)	0.35 (0-2.1)	0 (0-0)	0 (0-0.9)
Sporichthyaceae	0.3436	0.808470588	0 (0-0.1)	0.05 (0-0.4)	0 (0-0)	0 (0-0.3)	0 (0-0.1)	0 (0-0.1)	0 (0-0.1)	0 (0-0.2)
Streptomycetaceae	0.0786	0.099809524	0 (0-0.4)	0.2 (0-0.9)	0 (0-0.2)	0 (0-0.2)	0 (0-0.4)	0.2 (0-1.4)	0 (0-0)	0 (0-0.1)
Streptomyces	0.0522	0.063714706	0 (0-0)	0.05 (0-0.4)	0 (0-0.2)	0 (0-0.2)	0 (0-0.1)	0.15 (0-1)	0 (0-0)	0 (0-0.1)
Williamsiaceae	0.2071	0.352510638	0 (0-0.1)	0.05 (0-0.7)	0 (0-0)	0 (0-1.2)	0 (0-0.6)	0 (0-0.2)	0 (0-0)	0 (0-0)
Williamsia	0.2071	0.350802041	0 (0-0.1)	0.05 (0-0.7)	0 (0-0)	0 (0-1.2)	0 (0-0.6)	0 (0-0.2)	0 (0-0)	0 (0-0)
Rubrobacteria	0.9784	25.4384	0 (0-0.2)	0.1 (0-0.4)	0.2 (0-0.4)	0 (0-0.6)	0 (0-4)	0 (0-1)	0 (0-0)	0 (0-0.8)
Rubrobacterales	0.9784	14.676	0 (0-0.2)	0.1 (0-0.4)	0.2 (0-0.4)	0 (0-0.6)	0 (0-4)	0 (0-1)	0 (0-0)	0 (0-0.8)
Rubrobacteraceae	0.9784	39.136	0 (0-0.2)	0.1 (0-0.4)	0.2 (0-0.4)	0 (0-0.6)	0 (0-4)	0 (0-1)	0 (0-0)	0 (0-0.8)
Rubrobacter	0.9784	40.6036	0 (0-0.2)	0.1 (0-0.4)	0.2 (0-0.4)	0 (0-0.6)	0 (0-4)	0 (0-1)	0 (0-0)	0 (0-0.8)
Thermoleophilia	0.3786	0.894872727	0.5 (0-1.7)	2.8 (0-16.8)	0.8 (0-7.3)	0.3 (0-5.1)	0.1 (0-5.4)	0.75 (0-26.6)	0 (0-0.1)	0 (0-3.2)
Gaiellales	0.2339	0.375910714	0 (0-0.6)	1.7 (0-7.8)	0.7 (0-6.5)	0.2 (0-2.2)	0 (0-3.2)	0.2 (0-10.4)	0 (0-0)	0 (0-1.1)
Gaiellaceae	0.3079	0.703771429	0 (0-0.6)	1.65 (0-7.6)	0.7 (0-6.5)	0.1 (0-2.2)	0 (0-3)	0.2 (0-10.1)	0 (0-0)	0 (0-1.1)
Solirubrobacterales	0.2863	0.560152174	0.5 (0-1.1)	1.2 (0-9)	0.1 (0-0.9)	0.2 (0-2.9)	0.1 (0-4.8)	0.6 (0-16.2)	0 (0-0.1)	0 (0-2.1)
Patulibacteraceae	0.0026	0.002666667	0 (0-0)	0.05 (0-0.6)	0 (0-0)	0.1 (0-0.6)	0 (0-0)	0 (0-0.8)	0 (0-0)	0 (0-0.6)
Solirubrobacteraceae	0.143	0.204285714	0.5 (0-0.6)	0.35 (0-1.4)	0.1 (0-0.5)	0 (0-0.5)	0* (0-0)	0.35 (0-3.2)	0 (0-0)	0 (0-0.9)
Bacteroidetes	0.1752	0.24528	3.1 (0.3-3.1)	3.75 (0.5-62.8)	1.2 (0-2.9)	4.2 (0-22.9)	2.8 (0-15.5)	1.6 (0-16.1)	0.15 (0-1)	0.1 (0-44.6)
Bacteroidia	0.0304	0.034365217	1 (0-2.5)	2.55 (0-62.4)	0.3 (0-0.8)	3.7 (0-22.9)	0 (0-1.2)	0.1 (0-15.3)	0.15 (0-0.4)	0.05 (0-44.3)
Bacteroidales	0.0304	0.033365854	1 (0-2.5)	2.55 (0-62.4)	0.3 (0-0.8)	3.7 (0-22.9)	0 (0-1.2)	0.1 (0-15.3)	0.15 (0-0.4)	0.05 (0-44.3)
Paraprevotellaceae	0.016	0.017297297	0 (0-0.2)	0.2 (0-57.7)	0 (0-0)	0.3 (0-3.8)	0 (0-0)	0 (0-1.1)	0 (0-0.4)	0 (0-2)
Prevotella	0.0172	0.018302564	0 (0-0.2)	0.2 (0-0.8)	0 (0-0)	0.3 (0-3.8)	0 (0-0)	0 (0-1.1)	0 (0-0.4)	0 (0-2)
Bacteroidaceae	0.4362	1.342153846	0 (0-0)	0 (0-0.2)	0 (0-0.4)	0 (0-17.9)	0 (0-0)	0 (0-0)	0 (0-0.3)	0 (0-25.2)
Bacteroides	0.4362	1.293021429	0 (0-0)	0 (0-0.2)	0 (0-0.4)	0 (0-17.9)	0 (0-0)	0 (0-0)	0 (0-0.3)	0 (0-25.2)
Porphyromonadaceae	0.019	0.021111111	0.7 (0-2.5)	1.6 (0-12.6)	0.3 (0-0.6)	1.1 (0-16.5)	0 (0-1.2)	0.1 (0-15.2)	0 (0-0)	0.05 (0-41)
Porphyromonas	0.0186	0.020049351	0.7 (0-2.4)	1.45 (0-12.4)	0.3 (0-0.6)	1.1 (0-15.6)	0 (0-1)	0.1 (0-14)	0 (0-0)	0.05 (0-40.2)
Flavobacteriia	0.2786	0.5174	0.1 (0-0.8)	0.2 (0-2.1)	0.3 (0-1.2)	0.2 (0-1.6)	0 (0-0.3)	0.05 (0-0.8)	0 (0-0.6)	0 (0-2.6)
Flavobacteriales	0.2786	0.50148	0.1 (0-0.8)	0.2 (0-2.1)	0.3 (0-1.2)	0.2 (0-1.6)	0 (0-0.3)	0.05 (0-0.8)	0 (0-0.6)	0 (0-2.6)
Flavobacteriaceae	0.2786	0.602378378	0.1 (0-0.8)	0.2 (0-2.1)	0.3 (0-1.2)	0.2 (0-1.6)	0 (0-0.3)	0.05 (0-0.8)	0 (0-0.6)	0 (0-2.6)
Bergeyella	0.2713	0.577382051	0 (0-0.3)	0 (0-0.7)	0 (0-0.6)	0 (0-1.4)	0 (0-0)	0 (0-0.1)	0 (0-0)	0 (0-0.2)
Capnocytophaga	0.474	1.57368	0 (0-0.1)	0 (0-0.9)	0 (0-0.5)	0.1 (0-1)	0 (0-0)	0 (0-0.4)	0 (0-0.6)	0 (0-2.1)
Flavobacterium	0.9056	9.3956	0 (0-0.5)	0 (0-0.8)	0 (0-0.3)	0 (0-0.1)	0 (0-0)	0 (0-0.3)	0 (0-0)	0 (0-0)
Sphingobacteriia	0.3776	0.818133333	0.5 (0-1.3)	0.4 (0.2-1.8)	0.6 (0-1.7)	0.1 (0-0.9)	1.6 (0-15.5)	0.75 (0-1.5)	0 (0-0)	0 (0-0.1)
Sphingobacteriales	0.3776	0.809142857	0.5 (0-1.3)	0.4 (0.2-1.8)	0.6 (0-1.7)	0.1 (0-0.9)	1.6 (0-15.5)	0.75 (0-1.5)	0 (0-0)	0 (0-0.1)
Chitinophagaceae	0.753	4.633846154	0 (0-0.8)	0.25 (0-1.4)	0.2 (0-1.2)	0 (0-0.5)	0 (0-1.9)	0.35 (0-1.3)	0 (0-0)	0 (0-0.1)
Flavisolibacter	0.5812	2.41198	0 (0-0.5)	0.1 (0-0.9)	0 (0-1.1)	0 (0-0.4)	0 (0-1.9)	0.05 (0-1.2)	0 (0-0)	0 (0-0)
Flexibacteraceae	0.8313	7.389333333	0 (0-0.1)	0.1 (0-0.8)	0 (0-0.3)	0 (0-0.4)	0.3 (0-15.5)	0 (0-0.3)	0 (0-0)	0 (0-0)
Chloroflexi	0.7228	3.373066667	0.1 (0-2)	0.4 (0-1.7)	2.9 (0-4.1)	0 (0-2.3)	0 (0-11.9)	0.85 (0-2.8)	0 (0-0.1)	0 (0-0.5)
Chloroflexi	0.2419	0.3930875	0 (0-1.6)	0 (0-0.5)	0.5 (0-3.5)	0 (0-0.9)	0 (0-8.6)	0.15 (0-0.9)	0 (0-0.1)	0 (0-0.4)

Cyanobacteria	0.7246	5.0722	1.2 (0.4-4.5)	2.35 (0.2-42.1)	6 (0-12.1)	1.5 (0-11.7)	0.2 (0-16.3)	3.95 (0-9.4)	0 (0-0)	0 (0-1.6)
Chloroplast	0.251	0.435066667	1.2 (0.4-2.1)	1.65 (0.1-8.9)	1.3 (0-3.2)	1.1 (0-8.5)	0.1 (0-2.3)	2.2 (0-7.1)	0 (0-0)	0 (0-0.7)
Chlorophyta	0.2822	0.529125	0 (0-0)	0.05 (0-1.1)	0 (0-1.8)	0 (0-0.3)	0 (0-0.1)	0 (0-0.9)	0 (0-0)	0 (0-0)
Stramenopiles	0.0861	0.101960526	0 (0-0.7)	0.35 (0-7.1)	0 (0-1)	0.1 (0-7.4)	0 (0-1.5)	0.15 (0-5.9)	0 (0-0)	0 (0-0)
Streptophyta	0.7919	5.93925	1.2 (0.4-1.4)	0.7 (0-7.1)	0.3 (0-2.2)	0.1 (0-2.5)	0.1 (0-0.7)	0.1 (0-3.5)	0 (0-0)	0 (0-0.7)
Nostocophycideae	0.156	0.2028	0 (0-0.1)	0 (0-0.6)	0.8 (0-5.2)	0 (0-3.8)	0 (0-8.8)	0 (0-0.8)	0 (0-0)	0 (0-0)
Nostocales	0.1123	0.144385714	0 (0-0.1)	0 (0-0.6)	0.6 (0-5.2)	0 (0-3.8)	0 (0-8.6)	0 (0-0.8)	0 (0-0)	0 (0-0)
Nostocaceae	0.1151	0.153466667	0.8 (0-2.1)	0.35 (0-6.6)	0.3 (0-5.3)	0 (0-11.5)	0 (0-8.4)	0.1 (0-2.8)	0 (0-0.1)	0 (0-0.4)
Oscillatoriophycideae	0.5548	2.404133333	0 (0-1.7)	0.3 (0-34.9)	1.9 (0-3.6)	0.1 (0-2.4)	0 (0-6.6)	0.65 (0-2.5)	0 (0-0)	0 (0-0.4)
Chroococcales	1	22.5	0 (0-0.1)	0.15 (0-34.9)	0.9* (0-1.9)	0 (0-0.6)	0 (0-6.6)	0.2 (0-2.5)	0 (0-0)	0 (0-0.4)
Xenococcaceae	0.3766	1.004266667	0 (0-0)	0.05 (0-34.9)	0.2 (0-0.7)	0 (0-0.6)	0 (0-0)	0 (0-2.4)	0 (0-0)	0 (0-0.1)
Oscillatoriales	0.3715	0.759886364	0 (0-1.6)	0 (0-1.6)	0 (0-2.6)	0 (0-2)	0* (0-0)	0.1 (0-0.8)	0 (0-0)	0 (0-0)
Phormidiaceae	0.3715	0.92875	0 (0-1.6)	0 (0-1.6)	0 (0-2.6)	0 (0-2)	0* (0-0)	0.1 (0-0.8)	0 (0-0)	0 (0-0)
Phormidium	0.3715	0.99466129	0 (0-1.6)	0 (0-1.6)	0 (0-2.6)	0 (0-2)	0* (0-0)	0.1 (0-0.8)	0 (0-0)	0 (0-0)
Synechococcophycideae	0.4334	1.252044444	0 (0-0.2)	0 (0-0.5)	0 (0-5.7)	0 (0-0.7)	0 (0-0)	0 (0-0.4)	0 (0-0)	0 (0-0.5)
Pseudanabaenales	0.218	0.327	0 (0-0)	0 (0-0.5)	0 (0-1.2)	0 (0-0.6)	0 (0-0)	0 (0-0.4)	0 (0-0)	0 (0-0.4)
Pseudanabaenaceae	0.218	0.396363636	0 (0-0)	0 (0-0.5)	0 (0-1.2)	0 (0-0.6)	0 (0-0)	0 (0-0.4)	0 (0-0)	0 (0-0.4)
Firmicutes	0.7049	1.97372	18 (9.6-31.5)	16.3 (5-48.7)	16.4 (3.9-67.3)	15.1 (1.1-31.8)	8.2 (0-30.8)	9.55 (1-55.3)	8.45 (0-96.1)	5.55 (0.8-51)
Bacilli	0.5331	1.980085714	16.4 (7.8-31.5)	14.4 (4.3-45.7)	16.3 (3.9-67.1)	13.2 (0.3-28)	5.3 (0-19.2)	8.3 (1-51.9)	8.45 (0-96)	2.95 (0.1-50.4)
Bacillales	0.7703	4.951928571	9.6 (5.9-31.5)	10.05 (0.9-40.9)	9.9 (3.9-66.4)	5.6 (0-24.8)	5.1 (0-19.1)	3.55 (0.9-44.5)	0.1 (0-94.9)	1.85 (0.1-50.2)
Alicyclobacillaceae	0.9553	19.106	1.2 (0-20.3)	0.45 (0-2.2)	1 (0-4.8)	0.2 (0-2.9)	1.6 (0-6.4)	0.35 (0-4)	0 (0-0.2)	0.45 (0-11.5)
Alicyclobacillus	0.9681	26.7841	1.2 (0-20.3)	0.45 (0-2.2)	1 (0-4.8)	0.2 (0-2.9)	1.6 (0-6.4)	0.35 (0-4)	0 (0-0.2)	0.45 (0-11.5)
Bacillaceae	0.2738	0.576421053	3.8 (0.9-4.4)	3.55 (0-10.6)	4 (0-23.7)	1 (0-18.3)	0.1* (0-0.9)	1.5 (0.1-9.2)	0 (0-0.5)	0.1 (0-1.3)
Bacillus	0.17	0.261296296	3.8 (0.9-4.4)	3.4 (0-10.5)	1.8 (0-23.7)	1 (0-17)	0.1* (0-0.9)	1.35 (0.1-9.2)	0 (0-0)	0 (0-1.3)
Paenibacillaceae	0.0257	0.028957746	0 (0-0.3)	0.3 (0-1.6)	0 (0-1.2)	0.1 (0-2.2)	0* (0-0)	0.1 (0-1.4)	0 (0-0)	0 (0-0.1)
Ammoniphilus	0.0195	0.021296053	0 (0-0)	0 (0-0.7)	0 (0-0)	0 (0-0.3)	0 (0-0)	0 (0-0.7)	0 (0-0)	0 (0-0)
Paenibacillus	0.1292	0.184889655	0 (0-0.2)	0.15 (0-0.8)	0 (0-1.2)	0 (0-0.3)	0 (0-0)	0 (0-0.4)	0 (0-0)	0 (0-0.1)
Planococcaceae	0.0694	0.085415385	0.4 (0-1.6)	0.95 (0-2.3)	0.1 (0-1.9)	0.2 (0-1.1)	0* (0-0)	0.15 (0-5.5)	0 (0-0)	0 (0-0)
Rummeliibacillus	0.5974	2.609694737	0.4 (0-1.3)	0 (0-0.2)	0 (0-0.1)	0 (0-0.4)	0 (0-0)	0 (0-0)	0 (0-0)	0 (0-0)
Sporosarcina	0.0222	0.024568	0 (0-0)	0.1 (0-1.6)	0 (0-0.1)	0 (0-0.8)	0 (0-0)	0 (0-1.9)	0 (0-0)	0 (0-0)
Staphylococcaceae	0.6979	3.988	3.4 (1.9-7.3)	2.1 (0.2-33.9)	2.6 (0-65.7)	0.6 (0-22)	1.9 (0-12.6)	1.15 (0-30.4)	0 (0-94.4)	0.35 (0-42.9)
Macrococcus	0.2987	0.708345714	0.3 (0.1-7.3)	0.1 (0-32.2)	0 (0-0)	0 (0-10.2)	0 (0-0)	0 (0-28.5)	0 (0-0.4)	0 (0-0.1)
Salinicoccus	0.5364	2.023690909	0 (0-2.9)	0 (0-1.9)	0 (0-0.2)	0 (0-0.1)	0 (0-11.6)	0 (0-0.2)	0 (0-0)	0 (0-0)
Staphylococcus	0.7744	4.944246154	0.2 (0-1.8)	0.85 (0.2-8.6)	2.6 (0-65.6)	0.5 (0-14.1)	1 (0-4.4)	0.95 (0-3.4)	0 (0-94)	0.35 (0-42.9)
Exiguobacterales	0.3936	0.8856	0.1 (0-0.1)	0.1 (0-1.5)	0 (0-0.6)	0.1 (0-0.4)	0 (0-0.1)	0 (0-0.2)	0 (0-0)	0 (0-0.1)
Exiguobacteraceae	0.3936	1.124571429	0.1 (0-0.1)	0.1 (0-1.5)	0 (0-0.6)	0.1 (0-0.4)	0 (0-0.1)	0 (0-0.2)	0 (0-0)	0 (0-0.1)
Exiguobacterium	0.3936	1.126510345	0.1 (0-0.1)	0.1 (0-1.5)	0 (0-0.6)	0.1 (0-0.4)	0 (0-0.1)	0 (0-0.2)	0 (0-0)	0 (0-0.1)
Lactobacillales	0.7316	4.11525	1.8 (0-6.5)	0.9 (0-33.6)	0.8 (0-6.4)	2.9 (0.3-18.5)	0.1 (0-12.2)	0.25 (0-7.6)	0.55 (0-16.7)	0 (0-3.3)
Aerococcaceae	0.9449	15.1184	1 (0-2.8)	0.05 (0-1.4)	0.2 (0-0.9)	0.3 (0-3.2)	0 (0-0)	0.05 (0-1.1)	0.1 (0-14.5)	0 (0-1.1)
Abiotrophia	0.2563	0.5318225	0.2 (0-2.4)	0 (0-1.3)	0 (0-0.9)	0.3 (0-3.2)	0 (0-0)	0 (0-0.7)	0 (0-0.2)	0 (0-1.1)
Enterococcaceae	0.6708	3.5776	0 (0-0)	0 (0-2.2)	0 (0-0)	0 (0-1.8)	0 (0-0.6)	0 (0-4)	0 (0-0)	0 (0-0.3)
Streptococcaceae	0.3868	1.067034483	0.8 (0-3.7)	0.35 (0-33.5)	0.1 (0-5.5)	1.1 (0-17.9)	0 (0-0.5)	0.05 (0-7.6)	0.45 (0-2.2)	0 (0-3)
Streptococcus	0.5044	1.820226087	0.8 (0-3.5)	0.3 (0-33.3)	0.1 (0-5.5)	1.1 (0-17.9)	0 (0-0)	0.05 (0-7.6)	0.45 (0-2.2)	0 (0-2.1)
Clostridia	0.0452	0.053418182	1.4 (0-1.4)	1.45 (0-4.1)	0.1 (0-3.4)	1.7 (0.1-31.2)	2.9 (0-12.9)	0.65 (0-3)	0* (0-0)	0.95 (0-7.4)
Clostridiales	0.0305	0.0343125	0.5 (0-0.9)	0.85 (0-4)	0.1 (0-1.8)	1.5 (0.1-28.5)	2.8 (0-11.7)	0.6 (0-3)	0* (0-0)	0.65 (0-6.7)
Clostridiaceae	0.0018	0.001822785	0.3 (0-0.6)	0.35 (0-1.3)	0* (0-0.1)	0.5 (0-4.5)	0* (0-0.2)	0.45 (0-1.2)	0 (0-0)	0 (0-2.1)
Clostridium	0.0117	0.01213875	0 (0-0.3)	0.15 (0-0.6)	0 (0-0.1)	0.1 (0-4.5)	0 (0-0.1)	0.15 (0-1.1)	0 (0-0)	0 (0-0.7)
Lachnospiraceae	0.0443	0.052895522	0 (0-0)	0 (0-1.6)	0 (0-0.1)	0 (0-11.9)	0 (0-0.1)	0 (0-0.3)	0 (0-0)	0 (0-3.7)
Ruminococcus	0.3809	1.053823333	0 (0-0)	0 (0-0)	0 (0-0)	0 (0-1.2)	0 (0-0)	0 (0-0)	0 (0-0)	0 (0-0.2)
Blautia	0.0654	0.08101791	0 (0-0)	0 (0-1.1)	0 (0-0)	0 (0-7.3)	0 (0-0)	0 (0-0.1)	0 (0-0)	0 (0-0.3)
Peptostreptococcaceae	0.2646	0.5292	0 (0-0.2)	0.15 (0-1.2)	0 (0-1.4)	0.2 (0-0.6)	0 (0-11.4)	0.1 (0-1.3)	0 (0-0)	0 (0-0.2)
Ruminococcaceae	0.0038	0.004	0 (0-0)	0.1 (0-0.5)	0 (0-0.2)	0.1 (0-1.3)	0 (0-0)	0 (0-0.3)	0 (0-0)	0 (0-1.5)
Faecalibacterium	0.8676	7.20108	0 (0-0)	0 (0-0)	0 (0-0.1)	0 (0-0.8)	0 (0-0)	0 (0-0.2)	0 (0-0)	0 (0-0.7)
Veillonellaceae	0.0361	0.042470588	0 (0-0)	0 (0-0.4)	0 (0-0)	0 (0-10.7)	0 (0-0)	0 (0-0.1)	0 (0-0)	0 (0-1)
Megamonas	0.2077	0.359147917	0 (0-0)	0 (0-0.1)	0 (0-0)	0 (0-10.7)	0 (0-0)	0 (0-0.1)	0 (0-0)	0 (0-1)
Coriobacteriales	0.0941	0.114445946	0 (0-0)	0 (0-0.1)	0 (0-0)	0 (0-0.3)	0 (0-0.1)	0 (0-0.2)	0 (0-0)	0 (0-0.2)
Coriobacteriaceae	0.0941	0.123409836	0 (0-0)	0 (0-0.1)	0 (0-0)	0 (0-0.3)	0 (0-0.1)	0 (0-0.2)	0 (0-0)	0 (0-0.2)
Collinsella	0.2966	0.683827778	0 (0-0)	0 (0-0.1)	0 (0-0)	0 (0-0.3)	0 (0-0.1)	0 (0-0.1)	0 (0-0)	0 (0-0.2)
Erysipelotrichi	0.1442	0.178533333	0.2 (0-0.4)	0.1 (0-0.6)	0 (0-0)	0 (0-0.6)	0 (0-0)	0 (0-0.4)	0 (0-0.1)	0 (0-0.6)
Erysipelotrichales	0.1442	0.196636364	0.2 (0-0.4)	0.1 (0-0.6)	0 (0-0)	0 (0-0.6)	0 (0-0)	0 (0-0.4)	0 (0-0.1)	0 (0-0.6)
Coprobacillaceae	0.5388	1.874086957	0 (0-0)	0 (0-0)	0 (0-0)	0 (0-0.2)	0 (0-0)	0 (0-0)	0 (0-0)	0 (0-0)
Erysipelotrichaceae	0.1523	0.229886792	0.2 (0-0.4)	0.1 (0-0.6)	0 (0-0)	0 (0-0.6)	0 (0-0)	0 (0-0.4)	0 (0-0.1)	0 (0-0.6)
Eubacterium	0.2792	0.609831579	0 (0-0)	0 (0-0.4)	0 (0-0)	0 (0-0.6)	0 (0-0)	0 (0-0)	0 (0-0)	0 (0-0)
Fusobacteria	0.0119	0.0119	0.2 (0-0.9)	0.05 (0-1.5)	0 (0-0.3)	0.3 (0-38.3)	0* (0-0)	0.1 (0-0.4)	0 (0-0)	0.05 (0-13)
Fusobacteria	0.0119	0.012376	0.2 (0-0.9)	0.05 (0-1.5)	0 (0-0.3)	0.3 (0-38.3)	0* (0-0)	0.1 (0-0.4)	0 (0-0)	0.05 (0-13)
Fusobacteriales	0.0119	0.012170455	0.2 (0-0.9)	0.05 (0-1.5)	0 (0-0.3)	0.3 (0-38.3)	0* (0-0)	0.1 (0-0.4)	0 (0-0)	0.05 (0-13)
Fusobacteriaceae	0.0035	0.003636364	0 (0-0.1)	0.05 (0-1.2)	0 (0-0.3)	0.3 (0-38.3)	0 (0-0)	0.05 (0-0.4)	0 (0-0)	0 (0-11.8)
Fusobacterium	0.0085	0.008709877	0 (0-0.1)	0 (0-1.1)	0 (0-0)	0.1 (0-5.2)	0 (0-0)	0 (0-0.4)	0 (0-0)	0 (0-11.8)
Gemmatimonadetes	0.3248	0.5684	0.2 (0-0.7)	0 (0-0.8)	0 (0-0.7)	0 (0-0.2)	0 (0-1.3)	0 (0-1.1)	0 (0-0)	0 (0-0)
Planctomycetes	0.3264	0.6528	0 (0-0.2)	0.25 (0-1.7)	0.3 (0-1.4)	0.2 (0-1.6)	0 (0-1.4)	0.2 (0-1.7)	0 (0-0)	0 (0-0.4)
Planctomycetia	0.3101	0.6202	0 (0-0.2)	0.25 (0-1.7)	0.3 (0-1.1)	0.2 (0-1.6)	0 (0-1.4)	0.2 (0-1.7)	0 (0-0)	0 (0-0.4)
Gemmatales	0.5433	1.880653846	0 (0-0.2)	0.05 (0-1.3)	0.2 (0-0.4)	0.1 (0-0.7)	0 (0-1.4)	0.2 (0-1.4)	0 (0-0)	0 (0-0.4)
Gemmataceae	0.1849	0.29584	0 (0-0.2)	0 (0-0.6)	0 (0-0.1)	0 (0-0.3)	0* (0-0)	0.2 (0-1)	0 (0-0)	0 (0-0.1)
Isosphaeraceae	0.9091	10.38971429	0 (0-0)	0 (0-0.9)	0.1 (0-0.4)	0.1 (0-0.4)	0 (0-1.4)	0 (0-0.5)	0 (0-0)	0 (0-0.4)
Pirellulales	0.5681	2.324045455	0* (0-0)	0.15 (0-0.5)	0.1 (0-0.7)	0 (0-0.9)	0 (0-0.6)	0 (0-0.5)	0 (0-0)	0 (0-0)
Pirellulaceae	0.5681	2.065818182	0* (0-0)	0.15 (0-0.5)	0.1 (0-0.7)	0 (0-0.9)	0 (0-0.6)	0 (0-0.5)	0 (0-0)	0 (0-0)
Proteobacteria	0.0593	0.069183333	50 (24.1-76.5)	37.9 (18.9-56.5)	42.6 (0.2-50.1)	54.5 (6.8-98.1)	33.7 (1-45.5)	41.35 (2.4-96.9)	36.25 (1.9-99.6)	79.3 (18.3-96)
Alphaproteobacteria	0.3808	0.99008	11.4 (9-25)	10.45 (3.9-26.9)	26.5 (0-30.7)	6.3 (0-20.5)	18.2 (0-21.3)	9.5 (1.5-26.5)	0.45 (0-9.7)	0.45 (0-11.2)
Caulobacterales	0.0689	0.0795	0 (0-0.4)	0.55 (0-1.2)	0.5 (0-1.4)	0.1 (0-1)	0* (0-0)	0.2 (0-0.9)	0 (0-0)	0 (0-0.2)
Caulobacteraceae	0.0689	0.083515152	0 (0-0.4)	0.55 (0-1.2)	0.5 (0-1.4)	0.1 (0-1)	0* (0-0)	0.2 (0-0.9)	0 (0-0)	0 (0-0.2)

Rhizobiales	1	45	5.1 (0-9.4)	5.45 (1.4-11.3)	6.4 (0-12.8)	3.2 (0-8.1)	9.4 (0-14.9)	4.4 (0.3-10.9)	0 (0-0.4)	0.2 (0-4.9)
Aurantimonadaceae	0.2208	0.410790698	2.3 (0-3.3)	0.25 (0-1.3)	1 (0-2.1)	0.1 (0-0.5)	0.2 (0-3)	0.1 (0-1.1)	0 (0-0)	0 (0-0.3)
Bradyrhizobiaceae	0.136	0.190877193	0 (0-1.3)	1.2 (0.2-3.4)	0.2 (0-2.1)	0.1 (0-2.9)	0.4 (0-2.3)	0.7 (0-5.7)	0 (0-0)	0 (0-2.6)
Balneimonas	0.2369	0.446879545	0 (0-1.2)	0.85 (0-2.6)	0.1 (0-1.6)	0.1 (0-2.7)	0 (0-1.9)	0.35 (0-4.6)	0 (0-0)	0 (0-0.2)
Bradyrhizobium	0.0731	0.091928788	0 (0-0)	0.1 (0-0.9)	0 (0-0.6)	0 (0-0.5)	0 (0-0.4)	0.05 (0-0.5)	0 (0-0)	0 (0-2.2)
Hyphomicrobiaceae	0.3735	0.963870968	0 (0-1.1)	0.5 (0.1-2.3)	0.3 (0-4.4)	0.5 (0-1.2)	0.2 (0-1.2)	0.55 (0-1.8)	0 (0-0.4)	0 (0-0.5)
Devosia	0.8827	8.140455556	0 (0-1)	0.2 (0-0.7)	0.1 (0-0.7)	0.2 (0-1.2)	0.2 (0-1.1)	0.15 (0-0.6)	0 (0-0.4)	0 (0-0.1)
Rhodoplanes	0.0976	0.130658065	0 (0-0)	0.1 (0-1.2)	0.1 (0-1.7)	0 (0-0.7)	0 (0-0.1)	0.25 (0-0.7)	0 (0-0)	0 (0-0.5)
Methylobacteriaceae	0.9871	78.968	0.7 (0-1.8)	0.75 (0-2.7)	1.6 (0-3.5)	0.3 (0-1.7)	0 (0-5.2)	0.6 (0-1.4)	0 (0-0)	0 (0-0.7)
Methylobacterium	0.8358	6.306490909	0.2 (0-1.1)	0.3 (0-1.8)	1.3 (0-2.9)	0.1 (0-1.5)	0 (0-4.9)	0.3 (0-0.9)	0 (0-0)	0 (0-0.6)
Phyllobacteriaceae	0.2935	0.652222222	0.1 (0-0.6)	0.15 (0-0.7)	0.2* (0-1.3)	0 (0-0.3)	0 (0-0.5)	0.05 (0-0.4)	0 (0-0)	0 (0-0.3)
Rhizobiaceae	0.232	0.452682927	0 (0-1.2)	0.4 (0-1.7)	0.4 (0-1.2)	0.1 (0-2.7)	0 (0-4)	0.3 (0-1.3)	0 (0-0)	0 (0-2.4)
Agrobacterium	0.0775	0.098961538	0 (0-0.7)	0.25 (0-1.1)	0.2 (0-0.5)	0.1 (0-2.7)	0 (0-0.2)	0.1 (0-1)	0 (0-0)	0 (0-2.4)
Rhodobacterales	0.4333	1.146970588	4.5 (0.9-6)	1.35 (0.3-4.7)	3.2 (0-9.6)	0.2 (0-3)	0.1 (0-5.5)	0.95 (0-3)	0 (0-0.3)	0 (0-0.9)
Rhodobacteraceae	0.4333	1.283851852	4.5 (0.9-6)	1.35 (0.3-4.7)	3.2 (0-9.6)	0.2 (0-2.9)	0.1 (0-5.5)	0.95 (0-2.8)	0 (0-0.3)	0 (0-0.9)
Paracoccus	0.5744	2.270247619	0.7 (0-3.1)	0.25 (0-1.9)	0.6 (0-1.9)	0.1 (0-0.8)	0 (0-0.3)	0 (0-1.1)	0 (0-0.3)	0 (0-0)
Rubellimicrobium	0.656	3.202823529	1.4 (0-4)	0.5 (0-2.7)	0.6 (0-4.2)	0 (0-2.1)	0* (0-0)	0.1 (0-2.8)	0 (0-0)	0 (0-0.7)
Rhodospirillales	0.4737	1.4211	0.4 (0-2)	0.95 (0-5.7)	2.7 (0-6.3)	0.3 (0-4.6)	0 (0-4.1)	1.05 (0.2-3.8)	0 (0-0)	0 (0-0.8)
Acetobacteraceae	0.9674	25.79733333	0.3 (0-1.1)	0.4 (0-1.9)	2.6 (0-3.5)	0.2 (0-1.1)	0 (0-4)	0.55 (0-2.4)	0 (0-0)	0 (0-0.1)
Roseomonas	0.3464	0.898475	0.3 (0-0.9)	0.3 (0-0.7)	1.4 (0-2)	0.1 (0-0.7)	0 (0-1)	0.05 (0-1.4)	0 (0-0)	0 (0-0)
Rhodospirillaceae	0.0776	0.097	0.1 (0-0.9)	0.2 (0-3.7)	0.2 (0-3.7)	0.1 (0-4)	0* (0-0.6)	0.6 (0-1.7)	0 (0-0)	0 (0-0.7)
Skermanella	0.1034	0.140691803	0.1 (0-0.8)	0.15 (0-1.7)	0 (0-2.7)	0.1 (0-2.4)	0* (0-0.1)	0.45 (0-0.9)	0 (0-0)	0 (0-0.5)
Rickettsiales	0.2757	0.477173077	0 (0-0.2)	0.05 (0-0.9)	0.1 (0-3.7)	0 (0-0.9)	0 (0-0)	0.05 (0-1.6)	0 (0-0)	0 (0-0.2)
mitochondria	0.2156	0.374956522	0 (0-0.2)	0.05 (0-0.9)	0.1 (0-3.7)	0 (0-0.3)	0 (0-0)	0.05 (0-1.6)	0 (0-0)	0 (0-0.2)
Sphingomonadales	0.2625	0.4375	7.2 (1.4-7.9)	1.85 (0.7-9.6)	5.6 (0-6.9)	1.2 (0-3.8)	1 (0-5)	1.8 (0.1-8.9)	0.1 (0-9.7)	0 (0-5.2)
Erythrobacteraceae	0.2176	0.386844444	0.6 (0-0.9)	0.2 (0-0.8)	0.3* (0-2.5)	0 (0-0.1)	0 (0-4.1)	0.15 (0-0.6)	0 (0-0)	0 (0-0.3)
Sphingomonadaceae	0.5944	2.797176471	6.3 (0.8-7.9)	1.55 (0.5-8.6)	4.4 (0-5.2)	1.1 (0-3.6)	0.4 (0-3.1)	1.65 (0.1-8.8)	0.1 (0-9.7)	0 (0-4.9)
Kaistobacter	0.047	0.054943662	0 (0-0)	0.2 (0-1.7)	0 (0-0.2)	0.1 (0-0.6)	0 (0-0.2)	0 (0-3.4)	0 (0-0)	0 (0-0.1)
Novosphingobium	0.9438	15.66708	0 (0-0.2)	0.1 (0-0.5)	0 (0-0.4)	0 (0-0.1)	0 (0-0.4)	0 (0-0.1)	0 (0-0)	0 (0-0)
Sphingomonas	0.0824	0.1068625	3.1 (0.8-5.5)	0.35 (0.1-4.4)	2.4 (0-4.3)	0.5 (0-2.1)	0 (0-2.4)	0.15 (0-1.9)	0.1 (0-5.9)	0 (0-4.7)
Betaproteobacteria	0.0003	0.0003	9.7 (1.1-56.8)	9.95 (2-39.5)	1* (0-6.1)	14.9 (2.1-97.9)	3.4* (0-10.2)	11.45 (0.6-82.3)	0.4 (0-42.3)	20.45 (0.1-95.3)
Burkholderiales	0.0003	0.0003	2.3 (0-55.9)	7.6 (1.1-35.7)	0.6* (0-5.8)	6.8 (0.9-97.9)	3 (0-9.7)	10.95 (0.6-82.3)	0.4 (0-23.3)	19.9 (0-95.3)
Alcaligenaceae	0.756	5.04	1.2 (0-20.3)	0.45 (0-2.2)	1 (0-4.8)	0.2 (0-2.9)	1.6 (0-6.4)	0.35 (0-4)	0 (0-0.2)	0.45 (0-11.5)
Sutterella	0.7701	4.565592857	0 (0-0)	0 (0-0.4)	0 (0-0.4)	0 (0-2)	0 (0-0)	0 (0-0)	0 (0-0.2)	0 (0-1.2)
Burkholderiaceae	0.1465	0.213090909	0 (0-1.3)	0.05 (0-0.9)	0 (0-1.4)	0.3 (0-1.6)	0 (0-0)	0 (0-0.2)	0 (0-0)	0 (0-0.4)
Lautropia	0.4748	1.642016667	0 (0-0.2)	0 (0-0.5)	0 (0-0.5)	0 (0-0.5)	0 (0-0)	0 (0-0.1)	0 (0-0)	0 (0-0.2)
Comamonadaceae	0.5783	2.3132	0.3 (0-0.5)	0.7 (0-2.7)	0.5 (0-0.6)	1 (0-3.8)	0.5 (0-9.7)	0.45 (0-3.3)	0.3 (0-23.3)	0 (0-2.5)
Azohydromonas	0.9527	19.768525	0 (0-0)	0 (0-0.4)	0.1 (0-0.3)	0 (0-0.3)	0 (0-0)	0 (0-1.5)	0 (0-0)	0 (0-0)
Diaphorobacter	0.0007	0.000708537	0.2 (0-0.2)	0 (0-0.7)	0* (0-0.2)	0 (0-0)	0 (0-1.1)	0 (0-0.1)	0.25 (0-22.3)	0 (0-1.2)
Oxalobacteraceae	0.0001	0.0001	1.8 (0-54.2)	6.25 (0.5-34.7)	0.1 (0-3.6)	3 (0-97.9)	0* (0-3)	9.15 (0.6-82.3)	0* (0-0)	19.9 (0-95.2)
Janthinobacterium	0.3419	0.859930303	0 (0-0.1)	0 (0-0.5)	0 (0-0.4)	0 (0-0)	0 (0-0)	0.05 (0-2.5)	0 (0-0)	0 (0-0.1)
Ralstonia	0.0001	0.0001	0 (0-45.6)	4.25 (0.2-29.8)	0* (0-0)	2 (0-84.2)	0* (0-0.1)	7 (0.1-68.3)	0* (0-0)	16.95 (0-83.8)
Neisseriales	0.1336	0.176823529	1.1 (0.9-7.1)	1.45 (0-6.7)	0.3 (0-3)	2.3 (0-14.3)	0 (0-0.6)	0.3 (0-1.2)	0 (0-19)	0.15 (0-2.9)
Neisseriaceae	0.1336	0.184275862	1.1 (0.9-7.1)	1.45 (0-6.7)	0.3 (0-3)	2.3 (0-14.3)	0 (0-0.6)	0.3 (0-1.2)	0 (0-19)	0.15 (0-2.9)
Conchiformibius	0.2485	0.479662791	1.1 (0.5-5.9)	0.4 (0-2.6)	0* (0-2.8)	0.6 (0-8.7)	0 (0-0.6)	0 (0-0.7)	0 (0-19)	0 (0-2.4)
Deltaproteobacteria	0.6694	4.3511	0.3 (0-4.1)	0.2 (0-4.2)	0.5 (0-1.7)	0.2 (0-1.6)	0 (0-1.1)	0.7 (0-1.4)	0 (0-0)	0 (0-0.2)
Desulfovibrionales	0.8822	7.9398	0 (0-0.1)	0 (0-0)	0 (0-0)	0 (0-0.3)	0 (0-0)	0 (0-0.1)	0 (0-0)	0 (0-0.2)
Desulfomicrobiaceae	0.8822	8.822	0 (0-0.1)	0 (0-0)	0 (0-0)	0 (0-0.3)	0 (0-0)	0 (0-0.1)	0 (0-0)	0 (0-0.2)
Myxococcales	0.9397	10.571625	0.1 (0-4)	0.1 (0-3.7)	0.4 (0-1.5)	0 (0-1.1)	0 (0-1.1)	0.5 (0-1.1)	0 (0-0)	0 (0-0.1)
Spirobacillales	0.0947	0.118375	0 (0-0)	0.05 (0-1)	0 (0-0.2)	0 (0-0.5)	0 (0-0)	0 (0-0.5)	0 (0-0)	0 (0-0)
Epsilonproteobacteria	0.1582	0.216484211	0 (0-0)	0 (0-0.2)	0 (0-0)	0 (0-0.9)	0 (0-0.3)	0 (0-0.2)	0 (0-0)	0 (0-3.3)
Campylobacterales	0.1582	0.22246875	0 (0-0)	0 (0-0.2)	0 (0-0)	0 (0-0.9)	0 (0-0.3)	0 (0-0.2)	0 (0-0)	0 (0-3.3)
Gammaproteobacteria	0.8068	6.992266667	14 (4.2-15)	6.85 (1.5-29.2)	6.6 (0.2-20.2)	11.2 (0.2-39)	12.1 (0-20.5)	6.9 (0.2-88.1)	10.25 (0.5-99.3)	38.5 (0.4-92.5)
Cardiobacteriales	0.4066	0.963	0 (0-0.1)	0 (0-0.2)	0 (0-0.1)	0.2 (0-0.6)	0 (0-0)	0 (0-0.9)	0 (0-1.1)	0 (0-0.3)
Enterobacteriales	0.5266	1.692642857	0.3 (0-7.9)	0.95 (0-5.3)	0.7 (0.2-5.3)	0.6 (0.1-4.4)	2.4 (0-4.6)	0.55 (0-10)	0 (0-0)	0 (0-0.1)
Enterobacteriaceae	0.5266	1.755333333	0.3 (0-7.9)	0.95 (0-5.3)	0.7 (0.2-5.3)	0.6 (0.1-4.4)	2.4 (0-4.6)	0.55 (0-10)	0 (0-0)	0 (0-0.1)
Erwinia	0.1616	0.239514286	0.2 (0-4.8)	0.2 (0-2.8)	0.7 (0-2.1)	0.1 (0-1)	0.5 (0-1.4)	0 (0-2.7)	0 (0-0)	0 (0-0.1)
Klebsiella	0.122	0.171627119	0 (0-0.1)	0 (0-0.4)	0 (0-0.1)	0 (0-0.2)	0 (0-0)	0 (0-0.5)	0 (0-0)	0 (0-0)
Trabulsiella	0.2354	0.434182222	0 (0-0.4)	0.05 (0-0.3)	0.2 (0-1.5)	0 (0-0.1)	0 (0-0.9)	0 (0-0.3)	0 (0-0)	0 (0-0)
Halomonas	0.0313	0.035106757	0.2 (0-0.5)	0 (0-1.1)	0 (0-0.2)	0 (0-0)	0 (0-1)	0 (0-0.6)	0 (0-0)	0 (0-0)
Pasteurellales	0.0128	0.013395349	0 (0-1.6)	0.0045 (0-0.262)	0.003 (0-0.006)	0.01 (0-0.132)	0 (0-0.035)	0.003 (0-0.03)	0 (0-0.003)	0.0005 (0-0.113)
Pasteurellaceae	0.0128	0.013653333	0 (0-1.6)	0.45 (0-26.2)	0.3 (0-0.6)	1 (0-13.2)	0 (0-3.5)	0.3 (0-3)	0 (0-0.3)	0.05 (0-11.3)
Pseudomonadales	0.4483	1.26084375	3.9 (2.8-14)	1.9 (0.1-8.4)	1.7 (0-4.8)	4 (0-28.5)	1.4 (0-3.7)	1.95 (0-84)	10.05 (0.5-97.8)	34.25 (0-92.1)
Moraxellaceae	0.1756	0.270153846	2 (1.8-12.9)	0.95 (0-8.3)	0.6 (0-1.7)	2.3 (0-28.2)	0.2 (0-1.9)	1.1 (0-83.6)	8.9 (0-97.8)	33.1 (0-92)
Acinetobacter	0.9282	11.0058	0.1 (0-0.3)	0 (0-0.5)	0 (0-0.1)	0 (0-0.3)	0.1 (0-0.3)	0.1 (0-2.6)	0 (0-0.1)	0 (0-0.5)
Enhydrobacter	0.0409	0.047148611	0 (0-0.6)	0.1 (0-2.7)	0 (0-0.2)	0 (0-1.1)	0 (0-0)	0 (0-1.3)	0 (0-0)	0 (0-0.4)
Moraxella	0.0515	0.061949275	0 (0-0.1)	0 (0-0.1)	0 (0-0)	0 (0-0.3)	0 (0-0)	0 (0-0.1)	0 (0-0)	0 (0-0.4)
Pseudomonadaceae	0.3545	0.859393939	1.1 (0.8-2.1)	0.5 (0-5.1)	1.1 (0-3)	0.4 (0-4.5)	1.1 (0-2.6)	0.4 (0-1.5)	0 (0-2.8)	0.05 (0-2.1)
Pseudomonas	0.9282	12.8401	0.7 (0-1.1)	0.25 (0-4.6)	0.1 (0-0.9)	0.1 (0-4.5)	1 (0-1.3)	0.2 (0-1.5)	0 (0-2.8)	0.05 (0-0.3)
Xanthomonadales	0.5903	2.9515	0.3 (0-1.2)	0.55 (0.1-14.2)	0.6 (0-18.3)	0.4 (0-15.8)	3.3 (0-14.6)	0.6 (0-2.8)	0 (0-0)	0 (0-1)
Xanthomonadaceae	0.5792	2.438736842	0.3 (0-1)	0.5 (0.1-14.1)	0.6 (0-18.3)	0.3 (0-15.8)	3.3 (0-14.6)	0.55 (0-2.8)	0 (0-0)	0 (0-1)
Luteimonas	0.6222	2.869033333	0 (0-0.3)	0 (0-0.4)	0.1 (0-2)	0 (0-1.1)	0 (0-0.8)	0 (0-0.3)	0 (0-0)	0 (0-0.1)
Lysobacter	0.8278	5.725616667	0 (0-0.1)	0.05 (0-1)	0 (0-0.3)	0 (0-0.1)	0 (0-0)	0 (0-1.1)	0 (0-0)	0 (0-0)
Stenotrophomonas	0.253	0.49997619	0.1 (0-0.2)	0 (0-0.6)	0 (0-0)	0 (0-0.8)	0 (0-0)	0 (0-0.2)	0 (0-0)	0 (0-0.5)
Spirochaetes	0.0162	0.017446154	0 (0-0.2)	0.1 (0-0.3)	0 (0-0)	0 (0-2.4)	0 (0-0)	0 (0-0.4)	0 (0-0)	0 (0-0.9)
Spirochaetes	0.0166	0.017983333	0 (0-0.2)	0.1 (0-0.3)	0 (0-0)	0 (0-2.3)	0 (0-0)	0 (0-0.4)	0 (0-0)	0 (0-0.9)
Spirochaetales	0.0166	0.017785714	0 (0-0.2)	0.1 (0-0.3)	0 (0-0)	0 (0-2.3)	0 (0-0)	0 (0-0.4)	0 (0-0)	0 (0-0.9)
Spirochaetaceae	0.0166	0.018191781	0 (0-0.2)	0.1 (0-0.3)	0 (0-0)	0 (0-2.3)	0 (0-0)	0 (0-0.4)	0 (0-0)	0 (0-0.9)
Treponema	0.0166	0.017440506	0 (0-0.2)	0.1 (0-0.3)	0 (0-0)	0 (0-2.3)	0 (0-0)	0 (0-0.4)	0 (0-0)	0 (0-0.9)
Tenericutes	0.9713	13.5982	2.4* (0.1-42)	0 (0-0.6)	0 (0-0.1)	0.1 (0-13.2)	0 (0-7)	0 (0-6.4)	0 (0-82.6)	0 (0-3)

Mollicutes	0.9713	12.6269	2.4* (0.1-42)	0 (0-0.6)	0 (0-0.1)	0.1 (0-13.2)	0 (0-7)	0 (0-6.4)	0 (0-82.6)	0 (0-3)
Acholeplasmatales	0.2209	0.342775862	0 (0-0.1)	0 (0-0.1)	0 (0-0)	0 (0-1.3)	0 (0-0)	0 (0-0.1)	0 (0-0)	0 (0-3)
Acholeplasmataceae	0.2209	0.420761905	0 (0-0.1)	0 (0-0.1)	0 (0-0)	0 (0-1.3)	0 (0-0)	0 (0-0.1)	0 (0-0)	0 (0-3)
Acholeplasma	0.2209	0.398580435	0 (0-0.1)	0 (0-0.1)	0 (0-0)	0 (0-1.3)	0 (0-0)	0 (0-0.1)	0 (0-0)	0 (0-3)
Mycoplasmatales	0.5807	2.61315	2.4 (0-42)	0 (0-0.6)	0 (0-0.1)	0 (0-12.7)	0 (0-7)	0 (0-6.4)	0 (0-82.6)	0 (0-0.2)
Mycoplasmataceae	0.5807	2.580888889	2.4 (0-42)	0 (0-0.6)	0 (0-0.1)	0 (0-12.7)	0 (0-7)	0 (0-6.4)	0 (0-82.6)	0 (0-0.2)
Mycoplasma	0.1769	0.277032075	2.4* (0-5.9)	0 (0-0.1)	0 (0-0.1)	0 (0-9.7)	0 (0-7)	0 (0-5.5)	0 (0-3.7)	0 (0-0.2)
Thermi	0.1819	0.282955556	0 (0-0.1)	0 (0-4.2)	0 (0-0.2)	0 (0-0.5)	0 (0-1.2)	0.1 (0-1)	0 (0-0)	0 (0-0.1)
Deinococci	0.1819	0.262744444	0 (0-0.1)	0 (0-4.2)	0 (0-0.2)	0 (0-0.5)	0 (0-1.2)	0.1 (0-1)	0 (0-0)	0 (0-0.1)
Deinococcales	0.1819	0.264048387	0 (0-0.1)	0 (0-4.2)	0 (0-0.2)	0 (0-0.5)	0 (0-1.2)	0.1 (0-1)	0 (0-0)	0 (0-0.1)
Deinococcaceae	0.1819	0.285333333	0 (0-0.1)	0 (0-4.2)	0 (0-0.2)	0 (0-0.5)	0 (0-1.2)	0.1 (0-1)	0 (0-0)	0 (0-0.1)
Deinococcus	0.1819	0.290340385	0 (0-0.1)	0 (0-4.2)	0 (0-0.2)	0 (0-0.5)	0 (0-1.2)	0.1 (0-1)	0 (0-0)	0 (0-0.1)
Verrucomicrobia	0.7142	2.4997	0 (0-0.3)	0 (0-0.3)	0 (0-0.2)	0 (0-0.1)	0 (0-0)	0 (0-0.4)	0 (0-0)	0 (0-0.1)

Taxa present in at least 50% of dogs in at least one of the skin sites.
* Significant differences between allergic and healthy dogs when compared to the same skin sites.
**q-values adjusted based on the Benjamini and Hochberg False discovery rate.
